# Y Chromosome Lineages in Men of West African Descent

**DOI:** 10.1371/journal.pone.0029687

**Published:** 2012-01-25

**Authors:** Jada Benn Torres, Menahem B. Doura, Shomarka O. Y. Keita, Rick A. Kittles

**Affiliations:** 1 Department of Anthropology, University of Notre Dame, Notre Dame, Indiana, United States of America; 2 Department of Pharmacology and Physiology, George Washington University, Washington, District of Columbia, United States of America; 3 National Human Genome Center, Howard University, Washington, District of Columbia, United States of America; 4 Department of Medicine, University of Illinois at Chicago, Chicago, Illinois, United States of America; 5 Division of Epidemiology and Biostatistics, University of Illinois at Chicago, Chicago, Illinois, United States of America; 6 Institute of Human Genetics, University of Illinois at Chicago, Chicago, Illinois, United States of America; Institut de Biologia Evolutiva - Universitat Pompeu Fabra, Spain

## Abstract

The early African experience in the Americas is marked by the transatlantic slave trade from ∼1619 to 1850 and the rise of the plantation system. The origins of enslaved Africans were largely dependent on European preferences as well as the availability of potential laborers within Africa. Rice production was a key industry of many colonial South Carolina low country plantations. Accordingly, rice plantations owners within South Carolina often requested enslaved Africans from the so-called “Grain Coast” of western Africa (Senegal to Sierra Leone). Studies on the African origins of the enslaved within other regions of the Americas have been limited. To address the issue of origins of people of African descent within the Americas and understand more about the genetic heterogeneity present within Africa and the African Diaspora, we typed Y chromosome specific markers in 1,319 men consisting of 508 west and central Africans (from 12 populations), 188 Caribbeans (from 2 islands), 532 African Americans (AAs from Washington, DC and Columbia, SC), and 91 European Americans. Principal component and admixture analyses provide support for significant Grain Coast ancestry among African American men in South Carolina. AA men from DC and the Caribbean showed a closer affinity to populations from the Bight of Biafra. Furthermore, 30–40% of the paternal lineages in African descent populations in the Americas are of European ancestry. Diverse west African ancestries and sex-biased gene flow from EAs has contributed greatly to the genetic heterogeneity of African populations throughout the Americas and has significant implications for gene mapping efforts in these populations.

## Introduction

The European colonization of the Americas used labor from west and west central Africa, initially in the U.S. as indentured servants and later enslaved. Although the exact number is unknown and highly contested, it is estimated by some historians that between 8 to 12 million Africans were brought to the Americas in the transatlantic slave trade. Of this total, the vast majority were sold to European colonies in Latin America, only 4.5% of the enslaved Africans were imported to the United States, 7.8% to Jamaica, and 0.03% to the US Virgin Islands [1], [2], [3].

Enslaved Africans came from or through major coastal regions that had been labeled by Europeans as the Grain Coast (consisting of Senegal, Gambia, Guinea, Sierra Leone and parts of Liberia), Windward Coast (Ivory Coast and Liberia), Gold Coast (Ghana west of the Volta River), Bight of Benin (between the Volta and Benin Rivers), Bight of Biafra (east of the Benin River to Gabon), Central Africa (Gabon, Congo, and Angola), and the southern coast of Africa (from the cape of Good Hope to Cape Delgado, including the island of Madagascar).

In the sixteenth through nineteenth centuries west and west Central Africa were home to a range of societies and cultures of varying social organization from so-called “stateless” (village focused) societies to kingdoms [4], [5], [6]. The Senegambian region, with a long history of technical expertise in rice agriculture and making indigo dye, included a number of ethnic groups [5], [6], and Muslim kingdoms under Mande [7], as well as Fulani rule such as Futa Toro, Futa Jallon, and Bundu [8]. Further east in Lower Guinea [5] were the Akan speaking peoples with likely cultural origins in the second century CE (common era) in local iron working and trading societies at Begho [9] within what is now Ghana. The Akan-speaking peoples were organized into kingdoms [5], most prominent among them being Ashanti in the south, known for its use of gold in artistic production. Further east were societies that may have been the descendants of the Nok culture dated to the last centuries BC [9]: these include kingdoms such as Benin, famous for its metal sculpture, Dahomey, and the Yoruba states [10]. Adjacent to the Yoruba the Ibo/Igbo peoples lived in southeastern Nigeria, site of the likely ninth century archaeological site of Igbo Ekwu with interesting locally done bronze sculpture, and numerous glass beads obtained in long distance trade [9]. West Central Africa was home to several societies (such as Loango, Ndongo, Luba, Kuba), and notably the Kingdom of the Kongo, which shared some common metaphysical beliefs between them, although the elite in the Kongo eventually accepted Christianity [4].

Historians report that the majority of enslaved Africans that were brought to the United States tended to be from Sierra Leone, Senegambia, and the Gold Coast, though Africans throughout the West African coast were also imported [1], [11], [12]. Within the British Caribbean, including Jamaica, a large proportion of enslaved Africans had origins from the Bight of Biafra. In the Dutch Caribbean, including what is now the US Virgin island of St. Thomas, many enslaved Africans were imported from the Bight of Benin [2]. Genetic data obtained from mitochondria and Y chromosome analyses support these findings for the British Caribbean [13].

The differences in origins of enslaved Africans are partially the result of preferences that European settlers had for different skill sets. Other factors such as availability and economic trends also influenced where enslaved Africans were obtained [2], [3].

Wax [12] reports that not only were the majority of Africans imported directly from Africa but also that Africans from the Gold and Windward coasts were among the most favored by European American colonists. Within the Caribbean, colonists apparently preferred Akan peoples over those from Angola [11]. Within South Carolina evidence indicates that Africans with skills in rice cultivation were in greatest demand. Several historians suggest that in South Carolina upwards of 40% of the enslaved originated from the “Grain coast” regions of Senegambia and Sierra Leone [14], [15], [16].

However, within South Carolina, as in the rest of the Americas, although the identities of African peoples were transformed, even lost, in the context of enslavement and forced acculturation they were not rendered totally invisible to historical research [8], [17] and cultural memory as evidenced by some Brazilians' and Cubans' abilities to speak Yoruba dialects.

Individuals of African descent within the Americas have varied African origins and did have interactions with non-Africans, namely Europeans and indigenous Americans. European ancestry entered this sociopolitical defined group due to a range of practices including voluntary concubinage, marriage, and forced relations. European males predominated in this exchange, but sometimes European females were also involved. These differences have likely resulted in different population genetic histories. There have been few comprehensive studies that attempt to explore the genetic genealogical origins of African descendant populations in the United States and the Caribbean [13], [18]. Those studies that do consider origins generally only consider the mitochondrial locus. Both Ely et al. [19] and Salas et al. [18], [20] for example examine the maternal genetic ancestries of African Americans. Their conclusions are largely congruent with the historical record that African Americans descend from west and west central African populations. Within South America, specifically Brazil, the genetic data support the same conclusion that African-Brazilians also have west and west central African origin [21], [22], [23], [24] as well as some from southeastern Africa.

In comparisons of genetic variation across the genome and across continental populations, the variation found outside of Africa by and large tends to be a subset of the variation observed within African populations [25], [26]. This is generally attributed to the African origin of our species [27], [28] and the serial founder effects as humans migrated from Africa. Relatively few studies have examined African genetic diversity [29]. Although some studies have specifically considered regional genetic diversity within west or central Africa [23], [30], [31], [32], [33], [34] they generally investigate the mitochondrial lineages. Less has been published about paternal genetic variation within west and central Africa.

In this study, we examine Y-chromosome genetic variation in African descendant populations. In addition, we search for genetic evidence of substantial Senegambian “Grain Coast” ancestry in African American males from South Carolina. Finally, we consider the paternal African origins of several African descendant populations throughout the Americas. In doing this we hope to not only provide a genetic perspective to compliment historical investigations into the issue of African geographical origins but also contribute to the understanding of the genetic structure of African American populations. Understanding the variation present in these populations has implicit ramifications on admixture mapping and association studies in this admixed politically defined ‘macro-ethnic’ group [35].

## Materials and Methods

### Population Samples

**Figure 1:**
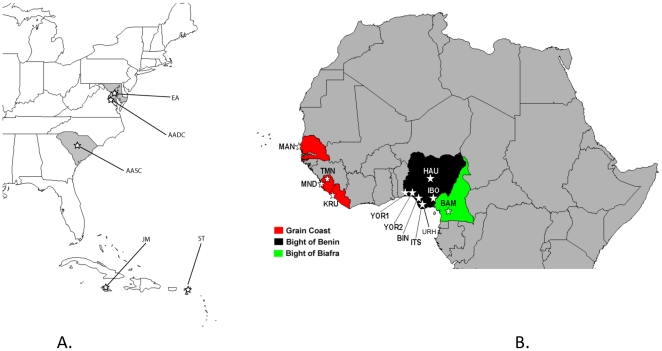
Maps showing location of (A) 5 populations in the Americas and (B) 12 West African populations sampled in the study.

Ethnic groups and sample size are given in Table 1 and a map of geographic locations is provided in Figure 1. DNA was extracted from whole blood in the lab using Purgene™ DNA extraction kit (Gentra Systems Inc., Minneapolis, MN). Blood was collected from unrelated men of west African descent enrolled in genetic studies from 1999–2002. Afro-Caribbean subjects were recruited from St. Thomas (N = 113) and Kingston, Jamaica (N = 75). African Americans were recruited from Washington, DC (N = 106) and Columbia, S.C. (N = 426). African American and Caribbean subjects were recruited for various cancer genetic studies [36], [37], [38], [39]. West African samples (N = 508), included 48 Bini from Edo State, Nigeria; 64 Ibo from Enugu State, Nigeria; 18 Hausa from Jos, Nigeria, 49 Yoruba from Ibadan, Nigeria; 61 Yoruba from Lagos, Nigeria; 28 Urhobo and 21 Itsekiri from the Warri Delta region of Nigeria; 34 Kru from Freetown, Liberia; 47 Mende and 34 Temne from Freetown, Sierra Leone [38]; 19 Mandinka from Dakar, Senegal; and 85 Bamileke from Yaounde, Cameroon [38], [40], [41], [42].

**Table 1:** Summary of Y chromosome diversity and frequency of YAP and M89 alleles.

Population/Location	Code	n	*k*	H	*h*	YAP ‘+’	M89 ‘T’	MPD
**African American**								
South Carolina	ASC	426	344	0.998±0.000	0.578±0.306	0.64	0.30	5.78±0.134
Washington, DC	ADC	106	96	0.998±0.001	0.548±0.294	0.59	0.28	5.48±0.258
Total	-	532	440	0.999±0.000	0.594±0.314	0.63	0.29	5.93±2.838
**Caribbean**								
Jamaica	JM	75	58	0.990±0.004	0.541±0.292	0.61	0.36	5.42±0.305
St. Thomas	ST	113	82	0.991±0.003	0.503±0.272	0.71	0.28	5.03±0.232
Total	-	188	140	0.995±0.001	0.519±0.279	0.67	0.32	5.19±2.522
**West African**								
Senegal	MAN	19	17	0.988±0.021	0.408±0.238	0.84	0.00	4.09±0.489
Sierra Leone	MND	47	43	0.996±0.005	0.451±0.250	0.96	0.04	4.51±0.330
Sierra Leone	TMN	34	30	0.991±0.010	0.371±0.213	1.00	0.00	3.71±0.330
Liberia	KRU	34	25	0.977±0.013	0.322±0.189	1.00	0.00	3.23±0.293
Nigeria	YO1	49	39	0.990±0.006	0.320±0.187	1.00	0.00	3.21±0.241
Nigeria	YO2	61	41	0.980±0.007	0.317±0.184	1.00	0.00	3.17±0.213
Nigeria	URH	28	20	0.963±0.021	0.307±0.183	1.00	0.00	3.07±0.311
Nigeria	ITS	21	20	0.995±0.016	0.411±0.238	0.95	0.00	4.12±0.466
Nigeria	BIN	48	39	0.989±0.007	0.327±0.190	0.98	0.00	3.27±0.247
Nigeria	HAU	18	17	0.993±0.021	0.526±0.298	0.50	0.39	5.26±0.629
Nigeria	IBO	64	51	0.987±0.006	0.372±0.211	0.88	0.00	3.72±0.238
Cameroon	BAM	85	45	0.976±0.005	0.317±0.183	0.94	0.00	3.17±0.180
Total	-	508	387	0.998±0.000	0.426±0.234	0.94	0.02	4.26±2.115
**European American**								
Washington, DC	EA	91	83	0.997±0.002	0.489±0.266	0.01	0.80	4.89±0.252

**Note**. n = number of Y-chromosomes, *k* = observed number of haplotypes, H = haplotype diversity, *h* = allelic diversity, MPD = mean pairwise differences of haplotypes.

In addition, 91 European American men from the Washington, DC area were included for comparisons [40]. This study was approved by the Institutional Review Board at Howard University. All samples were collected with written informed consent from each participant.

### Loci and Molecular Analysis

Eight Y chromosome specific short tandem repeats (YSTRs): DYS388, DYS389a, DYS389b, DYS390, DYS391, DYS392, DYS393, and DYS394 and one diallelic marker, DYS287 (Y Alu polymorphism) were typed in all samples. YAP is an ALU insertion on the non-recombining portion of the Y chromosome and is known to occur in high frequencies within African populations (haplogroup E) and rare in non-African populations [43]. Genotyping was completed using GeneScan on an ABI 377 DNA Sequencer. One Y-chromosome specific single nucleotide polymorphism (SNP), M89 C/T, was typed using Pyrosequencing methodology on the automated Pyrosequencing instrument, PSQ96. The ancestral M89 C allele is useful for distinguishing African paternal lineages which do not possess the YAP insertion (haplogroups A and B) from the non-African specific lineages (haplogroups C, D, F-T). While the C allele is at fixation within many African populations, the frequency of this allele varies considerably across the continent [44]. All primers, PCR conditions, and related methodology used in this study are available in Table S1.

### Statistical Analysis

To examine the Y chromosome diversity present in the African, Caribbean, and African American samples several diversity indices were estimated using Arlequin software [45]. The following indices were estimated for each population: the number of haplotypes (k), haplotype diversity (H), allelic diversity (h), and mean pairwise differences (MPD). Here Y haplotypes are defined by the combination of tested alleles at each locus. In addition, Y chromosome average variance (based on the Y specific microsatellites) was estimated for each population. R_ST_ values using a stepwise mutation model and an analysis of molecular variance (AMOVA) were estimated to examine genetic distances between populations as well as test for the presence of population structure within the sampled groups. These analyses were also performed using Arlequin software [45]. The genetic distances and the number of shared haplotypes were estimated between ethnic groups. The genetic distances were visualized using multidimensional scaling as implemented in the Statistica program.

Expansion times for the West African Y chromosome lineage groups were estimated using the equation, *t* = −*N_e_ ln*(1−*V*/*N_e_μ*) and a mutation rate (*μ*) for Y chromosome microsatellites of 4.26×10^−3^ (95% CI, 2.38×10^−3^–7.26×10^−3^) as suggested from a recent examination of Y chromosome microsatellite mutation rates [46]. The equation assumes a single-step mutation model for a haploid population and is robust for a population undergoing a strong bottleneck event followed by a rapid population expansion event [47]. Among west African samples, we estimated the average variance (*V*) in microsatellite repeat numbers for each Y chromosome group denoted by the YAP and M89 polymorphisms. The effective population size (*N*_e_) was assumed to be 1,000 and generation time of 25 years.

Finally, proportions of European paternal ancestry were estimated using the weighted least squares method [48] implemented in the ADMIX program (supplied by Dr. J.C. Long).

## Results

Eight YSTR loci, DYS287 (YAP), and M89 SNP were typed in 1,319 individuals from the 17 distinct populations found within the Americas, west and west central Africa in order to examine paternal lineages in men of African descent (see Table S2). Table 1 lists the diversity indices observed for each group. A total of 1,050 haplotypes were identified in all populations, of this 835 haplotypes were unique. Haplotype diversity measures ranged from 0.998 in both African American samples to 0.963 in the Urhobo. Likewise, allelic diversity measures ranged from 0.578 in the Columbia, SC sample to 0.307 in the Urhobo. The presence of the YAP insertion ranged from 100% in several West African groups, 50% in the Hausa to 1% in the European American population. In regards to the M89 marker, as expected, the majority of African populations in the study exhibited fixation of the C allele and the majority of the European American population carried the T allele. Within the Americas however, moderate levels of the T allele, ranging from 28–36% were observed with the populations. MPD values ranged from 3.07 in the Urhobo sample to 5.78 in the Columbia, SC sample. Considering the standard deviation, the average MPD for the African Americans and African Caribbean populations is actually higher than the average MPD for the African and European populations. Average variance in microsatellite repeat numbers for the YSTRs ranged from 0.337 in the Kru to 1.05 in the SC African Americans (data not shown). Like the MPD estimates, the average group variances were much higher in the Americas than in either the west African or European populations.

Variance in microsatellite repeat length (*V*) can be used to estimate *t*, the expansion times of the two African specific Y chromosome clusters in the west African populations. Our variance estimates were 0.44 for the YAP+ M98C cluster, and 1.01 for the YAP− M98C cluster, corresponding to age estimates of 2,706 and 6,796 years ago respectively using an effective population size (N_e_) of 1,000 individuals. The estimates and 95% confidence intervals are denoted in table 2. If N_e_ was between 750–2,000 individuals, the expansion time estimates would be between 2,633–2,758 and 6,336–7,159 for the YAP+ M98C and YAP− M98C clusters respectively.

**Table 2:** Expansion Times of West African Y Chromosome Groups.

Y group	Variance in allele size	Time in years	95% CI
YAP+ M89C	0.44	2,706	1,552–5,072
YAP− M89C	1.01	6,796	3,761–13,880

Several separate AMOVA analyses were conducted to examine the distribution of variation within and among groups (Table 3). The first analysis included all 17 populations grouped into three ethno-geographic groups. Group one consisted of all west and west Central African populations, group two consisted of African Americans and Caribbean samples, and group three consisted of the European American samples. The genetic variance due to differences among groups and differences among populations within groups was 14.7% and 14.5% respectively. Thus most of the genetic variance (70.8%) was due to differences within populations (Table 3).

**Table 3:** Partitions of Y chromosome molecular variance.

Sample		# of Pops.	# of Groups	Φ_ST_	P Value	%V	Φ_CT_	P Value	%V	Φ_SC_	P Value	%V
**Geographic Region:**												
Overall		17	3	0.29	0.000	70.8	0.147	0.015	14.7	0.169	0.000	14.5
West African regions		12	3	0.32	0.000	67.9	0.230	0.000	23.0	0.118	0.000	9.1
	Grain Coast	4	1	0.06	0.000							
	Bight of Benin	6	1	0.07	0.000							
	Bight of Biafra	2	1	0.29	0.000							
Americas		4	2	0.17	0.000	83.4	0.092	0.000	9.2	0.081	0.000	7.4
	Eastern U.S.	2	1	0.1	0.000							
	Caribbean	2	1	0	0.301							

**Φ_ST_ = Within populations; Φ_CT_ = Among groups; Φ_SC_ = Among populations within groups; %V = Percent of the variance.**

The next series of AMOVA analyses examined the distribution of genetic variance among the populations of African descent. One analysis within this series only included west and west central African populations. Each of the 12 west and west central African populations was first grouped according to three geographic regions, Grain Coast, Bight of Benin, and the Bight of Biafra. The majority of the variation, 67.9%, was within the WA populations while the least amount of variation, 9.1%, was observed between the populations within regions. In the next analysis, each of the west and west central African populations were considered separately. The populations within the Bight of Biafra have the highest R_ST_ value, at 0.294 while the populations from the Grain Coast have the lowest R_ST_ value, at 0.067. Additional analyses considered samples from the Americas. In these analyses the African American and Caribbean population were made into two groups, the Eastern United States versus the Caribbean. Most the variation, 83.4%, can be attributed to inter-populational differences in the Americas. Two additional AMOVAs were competed that only included the African American populations or only the Caribbean populations. The US populations had higher R_ST_ values than that observed in the Caribbean population and the inter-populational variation within the Caribbean was insignificant.

**Figure 2:**
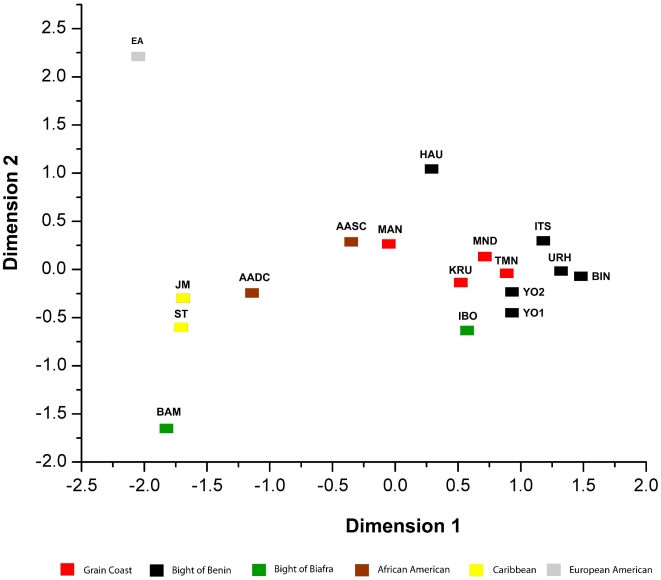
Plot of the first two principal components of a Y chromosome genetic distance matrix estimated for 17 populations.

Genetic distances were calculated between all populations (Table 4) and then analyzed with multiple dimensional scaling (MDS) (Figure 2). The data fit the reduced dimensions well as the stress value was low at 5.7% for a two dimensional ordinate plot (R^2^ = 0.982). Most of the African populations form a large cluster. Although included in this cluster, both the Hausa and one African American population, South Carolina, are on the peripheries of this group. The west central African Bamileke, represent the only African population that is not part of the aforementioned cluster. Instead, the Bamileke fell into a second cluster formed with the Washington DC African American and the Caribbean populations. As expected, the final population considered, European Americans, fell outside of either the African and African American cluster.

**Table 4:** Matrix of shared Y chromosome haplotypes and R_ST_ genetic distances.

	AASC	AADC	JM	ST	MAN	MND	TMN	KRU	YO1	YO2	URH	ITS	BIN	HAU	IBO	BAM	EA
**AASC**		2	2	4	4	11	7	11	16	19	6	6	17	6	16	3	7
**AADC**	0.116		8	8	0	0	0	0	0	0	0	0	0	0	2	8	0
**JM**	0.156	0.01		18	0	0	0	0	0	0	0	0	0	0	0	9	2
**ST**	0.194	0.025	−0.002		0	0	0	0	0	1	0	1	0	0	2	17	1
**MAN**	0.086	0.193	0.252	0.271		2	0	3	3	3	1	1	2	2	1	0	0
**MND**	0.096	0.241	0.337	0.352	0.119		4	4	1	5	3	1	3	3	4	0	0
**TMN**	0.104	0.276	0.353	0.361	0.161	0.064		2	1	3	1	1	2	0	0	0	0
**KRU**	0.096	0.223	0.315	0.323	0.142	0.11	0.13		7	7	3	2	4	4	3	0	0
**YO1**	0.137	0.292	0.375	0.374	0.208	0.161	0.077	0.045		9	4	1	11	4	5	0	0
**YO2**	0.119	0.29	0.371	0.373	0.176	0.137	0.041	0.044	−0.009		7	3	10	5	9	0	0
**URH**	0.172	0.326	0.413	0.415	0.226	0.065	0.045	0.235	0.161	0.137		1	6	2	1	0	0
**ITS**	0.145	0.307	0.393	0.406	0.266	0.059	0.047	0.241	0.191	0.154	0.025		5	1	1	0	0
**BIN**	0.187	0.375	0.465	0.463	0.286	0.104	0.043	0.21	0.132	0.104	0.024	0.025		4	5	0	0
**HAU**	0.03	0.242	0.317	0.364	0.156	0.094	0.161	0.186	0.242	0.204	0.231	0.158	0.248		4	0	1
**IBO**	0.12	0.238	0.325	0.323	0.176	0.148	0.099	0.032	0.025	0.024	0.182	0.188	0.153	0.242		0	0
**BAM**	0.27	0.127	0.143	0.091	0.4	0.441	0.457	0.442	0.458	0.454	0.496	0.504	0.543	0.54	0.383		0
**EA**	0.233	0.33	0.34	0.4	0.482	0.521	0.56	0.535	0.591	0.574	0.621	0.577	0.636	0.357	0.567	0.613	

**Note.** The number of shared Y haplotypes are above the diagonal. Values below the diagonal are RST genetic distances. Underlined values are significant (P>0.001).

The amounts of paternal genetic contributions made by Europeans to the African American populations included in this study were similar and varied between 30–40% (Table 5). One of the Caribbean populations, Jamaica, had the highest amount of European admixture at 41.1%. The African American population from South Carolina had the least amount of European admixture, only 32.3%.

**Table 5:** Estimates of European paternal ancestry (%) in African descent populations in the Americas.

Population	% European	95% CI
South Carolina	32.3	24.7–39.9
Washington, DC	36.6	18.8–54.4
St. Thomas, VI	33.7	13.0–54.4
Jamaica	41.1	20.8–61.4

## Discussion

Like demonstrated in previous studies, the frequency of the YAP insertion within the African or African derived populations is generally in higher frequency than in European derived populations [43], [49]. Interestingly, the frequency of the YAP insertion observed in one African population, the Hausa is lower than the percentages in the African American and Caribbean populations. Furthermore, according to the MDS plot (figure 2) the Hausa appear somewhat distinct from the other African populations. Hausa were the only Afro-Asiatic speakers included in this study, all other groups were Niger-Congo speakers. As mentioned above, the YAP insertion and the M89C allele are informative regarding African ancestry. The low frequency of the Y chromosome ALU insertion among Hausa speakers may indicate that the Hausa recently experienced gene flow from non-African populations, have ancestors who were involved in a back migration from west Asia [44], [50], or alternatively represent a genetically distinct African population, though these notions all warrant further investigation. In addition, the YAP and M89 markers are also indicative of the Y chromosome haplogroup [51]. The YAP+/M89C haplotype falls in to haplogroup E, which is in the highest frequency in Africa. YAP−/M89C haplotype may fall into several haplogroups, A, B, or C. Both haplogroups A and B are exclusive to the African continent while haplogroup C is found in varying frequency in Asia, Oceania, and the Americas. The final haplotype observed the current sample, YAP−/M89T is found in all other Y chromosome haplogroups outside of Africa [51]. In regard to the current sample, this illustrates that the vast majority of African Americans and African Caribbeans, about 70%, fall into an African Y chromosome haplogroup.

We argue that our time estimate of 2,706 years ago (95% CI; 1,552–5,072) for the expansion of Y group YAP+ M89C reflects the expansion of haplogroup E. Interestingly, this is concurrent with the time of the Bantu linguistic expansion. While the only Bantu speakers included in this study are the Bamileke, it is likely that this time estimate may represent another cultural expansion or interactions of populations across west and west central Africa.

Across the different diversity indices we explored (allelic and haplotypic) there is a general trend of greater diversity in the African American and African Caribbean populations relative to the European or west African populations. The high levels of variation observed in the Americas are likely a result of some combination of sample size and admixture. Within the Americas, the African American populations tend to exhibit slightly more haplotypic diversity than the African Caribbean populations. This trend is reversed when considering the frequency of the YAP insertion and the M89 T allele where the Caribbean populations have a higher frequency of both the YAP insertion and the M89 T allele. The difference in the diversity indices is most likely a function of the difference in sample size, while the difference in the frequencies of the YAP insertion and M89 allele are indicative of varying levels of admixture across the different populations.

The AMOVA analyses indicate that there is population structure within the west and west central African populations as well as the populations of African descent within the Americas. Several recent studies using autosomal DNA markers however have not detected significant population structure among west African populations [52], [53], [54]. The current study uses YSTRs and comes from individuals spread across west and west central Africa. A comparable study by Cruciani and colleagues considered 77 biallelic markers from the non-recombining region of the Y chromosome in several west and west central African populations [44]. In the Cruciani et al. study, population substructure was present within Sub-Saharan populations combined as well as in smaller geographical regions. The distribution of genetic variation within those sampled populations was similar to what was observed in this study. In another study that also considered Y chromosome markers, Wood et al. [55], found comparable levels of population substructure in 16 west and west central African populations. Considering the previous work, the current study is concordant in that population substructure is present within the Y chromosome among African and African derived populations.

In addition to the substructure observed in African populations, population structure was also detected within African descent populations in the Americas. The AA population rather than the Caribbean population were more influential on the structure seen within the Americas. When analyzed as separate geographic regions, the Caribbean populations had a small and insignificant R_ST_ value, whereas the AAs had a larger and significant measure. Evidence suggesting population stratification within AAs but not in Caribbeans was also revealed in the genetic distances (Table 4).

The differences in genetic distances may be the result of a smaller Caribbean sample size or overall homogeneity on the islands. Other studies that considered Caribbean populations have found evidence of population stratification within some but not all island populations [36]. In particular, those populations that have historically been isolated tend to lack population substructure. In regards to the African Americans, a study by Kayser et al [56], examined genetic diversity for nine Y- STRs, and suggested that no population stratification was observed among ten African American communities across the United States. The difference in the population substructure observed in the current study and Kayser et al's study do not appear to be the result of a difference in statistical analysis but instead in the populations sampled. In the Kayser et al. study, most of the African American populations had genetic distances that were effectively zero between each of the African American population [56].

Visualization of the genetic distances in the MDS plots illustrates a strong geographical relationship between the African populations. Within the mega cluster of African populations, there is a geographical distribution of the populations. Groups from the Grain Coast generally fall together, as do groups from the Bight of Benin. One African American population, those from South Carolina, cluster with the African populations. Notably, the South Carolina population falls nearest to the Grain Coast populations. Ethnohistorical records indicate a relationship between African Americans within this region of the United States and West Africans from Senegal, Gambia, and Sierra Leone. Based on such records it has been suggested that many African Americans within South Carolina originate from the Grain Coast region of West Africa. Furthermore, Africans from this region were sought-after and imported to the Americas for their knowledge of rice cultivation [8], [15], [17]. The current study is the first to test this hypothesis using genetic data. The other African derived groups from the Americas form a separate cluster and are closest to one outlying African group from the Bight of Biafra. Given that Caribbean slave census records collected in the 19th century indicate that many individuals were from the Bight of Biafra, this result appears consistent with historical data [57].

Admixture analyses indicate that similar levels, between 30–40%, of genetic contributions from Europeans were made to African derived communities throughout the Americas. This is in agreement with prior estimates based on Y-chromosomes data made for African derived populations in North America and the Caribbean [13], [58]. The sample from South Carolina was least admixed which contrasts with the Jamaican sample that was the most admixed African derived population sampled from the Americas. Moreover, the Jamaican admixture estimate of 41.1%, fell outside of the confidence interval for South Carolina, suggesting that the admixture in South Carolina is significantly different than the Jamaican estimate. Though sample size may have a role in this difference it does not seem as likely given that the admixture estimates of the other African American and Caribbean samples are very similar to the estimate from the South Carolinian population. As the analyses based on genetic distances and the admixture estimate indicates, the South Carolinian population is somewhat distinct from the Washington, DC AA and Caribbean populations.

Within a historical context, both population isolation and admixture may have had a considerable impact on the population history of the South Carolinian population. South Carolina was one of three focal points in the United States for slave labor during colonial times [59]. By the mid 1700's, Africans outnumbered Europeans in a two to one ratio in the region [60]. Additionally, enslaved Africans built labor camps within the ‘frontier zones’ of South Carolina in relative isolation from European planters [61]. Although the slave codes prohibited mixing between the two groups, there existed an assymetrical power relationship in which many European planters exploited. In some locations within South Carolina however, physical and/or social isolation may have effectively limited the opportunities for gene flow between African and Europeans as well as other African American groups within these South Carolinian populations. Furthermore, both the current study as well as a previous study found comparatively low levels of European genetic contribution to South Carolinian populations, specifically the Gullah, relative to other US African American populations [62].

The population histories of African American and African Caribbean populations are a result of political, social, and environmental experiences beginning with the post-Colombian colonization of the Americas. These communities like other segments within the African Diaspora, exhibit varying levels of genetic heterogeneity, population substructure, and admixture. Although there may have been some preferences for distinct west Africans by slave traders and colonial purchasers, enslaved Africans came from all sections of the west African coast [12]. As such, African and African-derived communities are ideal populations to include in genetic studies due to the characteristically high levels of heterogeneity found within these groups. Consequently, the genetic variation and population structure found within African American and African Caribbean populations has significant implications in association and gene mapping studies. As such, analytical approaches must correct for varied ancestries in order to avoid spurious associations. In addition, the complex relationship between genetic variation and population history as exhibited in African-derived communities is not only useful in efforts to unravel causes of human disease, but is informative about past events and relationships. As demonstrated here, genetic data support continuity between these communities and the African continent. Combined with ethno-historic data, this research is a clear example of how genetic applications can complement our knowledge of the past.

## Supporting Information

Table S1 **Y chromosome primers and PCR conditions.**
(PDF)

Table S2 **Y chromosome haplotypes observed.**
(PDF)
