# Supplementary material for: Y Chromosome Lineages in Men of West African Descent
Source: PLoS One. 2012 Jan 25;7(1):e29687. doi: 10.1371/journal.pone.0029687 (PMC3266241; doi:10.1371/journal.pone.0029687)
Supplement: Table S1 — Y chromosome primers and PCR conditions. (PDF) [file pone.0029687.s001.pdf]

PCR Analysis

Eight YSTRs (DYS388, DYS389a, DYS389b, DYS390, DYS391, DYS392, DYS393, and DYS394) and one diallelic marker (YAP) were typed using florescent-labeled primers listed in the table below. PCR reactions were performed in two multiplex reactions. Multiplex reaction one contained primers for DYS389, DYS390, DYS391, DYS393, and DYS394. Multiplex reaction two contained primers for DYS388, DYS392, and YAP. Both Multiplex PCRs were performed in a 25μl volume with 2mM MgCl2, 40ng template DNA, 10 pmol each primer, and .6 U of AmpliTaq Gold DNA Polymerase. The cycling conditions for both multiplexes were as follows: initial incubation at 95°C for 12 min followed by 35 cycles of 94°C for 1 min, 55°C for 1 min, 72°C for 2 min, with a final extension of 72°C for 10 min. PCR products were then pooled and run on ABI 377 DNA Sequencer or ABI 3100 Genetic Analyzer with 500 ROX size standard. One SNP (M89) was also typed using Pyrosequencing methodology. Forward and reverse primers for M89 amplification were 5'-BIOTIN-AGCTGCTCAGGTACACACAGAGT–3'and 5'-CCCACAGAAGGATGCTGC – 3'respectively. PCR amplification was performed in a 50μl reaction containing 40 ng of genomic DNA, 20 pmol of each primer, 1x PCR buffer II (Perkin-Elmer), 2 mM MgCl2, 0.2 mM dNTP, and .6 U of AmpliTaq gold DNA Polymerase. Cycling conditions consisted of an initial incubation at 95°C for 12 min, followed by 50 cycles of 95°C for 30 s, 54°C for 20 s, and 72°C for 30 s, with a final extension of 72°C for 10 min. Snp Genotyping was done on an automated pyrosequencing instrument (PSQ96) with the following sequencing primer: 5'-AAGGTTATGTACAAAAATCT–3'.

Locus	Type	Primer	Primer Sequence	Conditions
DYS388	tri	DYS388F	GTGAGTTAGCCGTTTAGCGA	triplex
		DYS388R	CAGATCGCAACCACTGCG	
DYS389a	tetra	DYS389F	CCAACTCTCATCTGTATTATCTATG	5 plex
		DYS389R	TCTTATCTCCACCCACCAGA	
DYS389b	tetra	DYS389F	CCAACTCTCATCTGTATTATCTATG	5 plex
		DYS389R	TCTTATCTCCACCCACCAGA	
DYS390	tetra	DYS390F	TATATTTTACACTTTTGGGCC	5 plex
		DYS390R	TGACAGTAAAATGAACACATTGC	
DYS391	tetra	DYS391F	CTATTCATTCAATCATACACCCA	5 plex
		DYS391R	GATTCTTTGTGGTGGGTCTG	
DYS392	tri	DYS392F	TCATTAATCTAGCTTTTAAAAACAA	triplex
		DYS392R	AGACCCAGTTGATGCAATGT	
DYS393	tetra	DYS393F	GTGGTCTTCTACTTGTGTCAATAC	5 plex
		DYS393R	AACTCAAGTCCAAAAAATGAGG	
DYS394	tetra	DYS394F	GACTACTGAGTTTCTGCAATAGTG	5 plex
		DYS394R	AGCACTGCACCTGGAAATAG	
YAP	Alu ins	YAPF	AGGACTAGCAATAGCAGGGGAAGA	triplex
		YAPR	ACTGCTAAAAGGGGATGGAT	
