# Supplementary material for: Y Chromosome Lineages in Men of West African Descent
Source: PLoS One. 2012 Jan 25;7(1):e29687. doi: 10.1371/journal.pone.0029687 (PMC3266241; doi:10.1371/journal.pone.0029687)
Supplement: Table S2 — Y chromosome haplotypes observed. (PDF) [file pone.0029687.s002.pdf]

**APPENDIX: Y chromosome haplotypes observed in populations**

| | Y chromosome haplotypes | | | | | | | | | | Populations | | | | | | | | | | | | | | | | | |
|---|---|---|---|---|---|---|---|---|---|---|---|---|---|---|---|---|---|---|---|---|---|---|---|---|---|---|---|---|
| **Haplotype** | **DYS388** | **DYS389A** | **DYS389B** | **DYS390** | **DYS391** | **DYS392** | **DYS393** | **DYS394** | **YAP** | **M89** | **SC** | **DC** | **CM** | **BN** | **EA** | **IBO** | **HAU** | **YOR1** | **ITS** | **JM** | **KRU** | **MAN** | **MND** | **ST** | **TMN** | **URH** | **YOR2** | **Total** |
| H001 | 12 | 15 | 32 | 25 | 11 | 12 | 13 | 15 | + | C | 1 | 0 | 0 | 0 | 0 | 0 | 0 | 0 | 0 | 0 | 0 | 0 | 0 | 0 | 0 | 0 | 0 | 1 |
| H002 | 12 | 13 | 28 | 21 | 12 | 10 | 13 | 14 | + | C | 1 | 0 | 0 | 0 | 0 | 0 | 0 | 0 | 0 | 0 | 0 | 0 | 0 | 0 | 0 | 0 | 0 | 1 |
| H003 | 12 | 14 | 31 | 24 | 11 | 12 | 13 | 15 | + | C | 1 | 0 | 0 | 0 | 0 | 0 | 0 | 0 | 0 | 0 | 0 | 0 | 0 | 0 | 0 | 0 | 0 | 1 |
| H004 | 12 | 14 | 31 | 24 | 11 | 10 | 13 | 15 | + | C | 1 | 0 | 0 | 0 | 0 | 0 | 0 | 0 | 0 | 0 | 0 | 0 | 0 | 0 | 0 | 0 | 0 | 1 |
| H005 | 12 | 13 | 29 | 26 | 12 | 10 | 13 | 14 | + | C | 1 | 0 | 0 | 0 | 0 | 0 | 0 | 0 | 0 | 0 | 0 | 0 | 0 | 0 | 0 | 0 | 0 | 1 |
| H006 | 12 | 13 | 30 | 20 | 11 | 10 | 15 | 15 | + | C | 1 | 0 | 0 | 0 | 0 | 0 | 0 | 0 | 0 | 0 | 0 | 0 | 0 | 0 | 0 | 0 | 0 | 1 |
| H007 | 12 | 13 | 31 | 21 | 11 | 10 | 15 | 17 | + | C | 1 | 0 | 0 | 0 | 0 | 0 | 0 | 0 | 0 | 0 | 0 | 0 | 0 | 0 | 0 | 0 | 0 | 1 |
| H008 | 12 | 13 | 30 | 25 | 12 | 10 | 13 | 14 | + | C | 1 | 0 | 0 | 0 | 0 | 0 | 0 | 0 | 0 | 0 | 0 | 0 | 0 | 0 | 0 | 0 | 0 | 1 |
| H009 | 12 | 13 | 30 | 22 | 11 | 12 | 14 | 17 | - | T | 1 | 0 | 0 | 0 | 0 | 0 | 0 | 0 | 0 | 0 | 0 | 0 | 0 | 0 | 0 | 0 | 0 | 1 |
| H010 | 12 | 14 | 30 | 23 | 12 | 12 | 13 | 13 | - | T | 1 | 0 | 0 | 0 | 0 | 0 | 0 | 0 | 0 | 0 | 0 | 0 | 0 | 0 | 0 | 0 | 0 | 1 |
| H011 | 12 | 14 | 31 | 22 | 11 | 12 | 13 | 16 | + | C | 1 | 0 | 0 | 1 | 0 | 0 | 0 | 0 | 0 | 0 | 0 | 0 | 0 | 0 | 0 | 0 | 0 | 2 |
| H012 | 12 | 15 | 30 | 24 | 11 | 12 | 13 | 14 | - | T | 1 | 0 | 0 | 0 | 0 | 0 | 0 | 0 | 0 | 0 | 0 | 0 | 0 | 0 | 0 | 0 | 0 | 1 |
| H013 | 15 | 14 | 31 | 21 | 11 | 10 | 13 | 13 | - | T | 1 | 0 | 0 | 0 | 0 | 0 | 0 | 0 | 0 | 0 | 0 | 0 | 0 | 0 | 0 | 0 | 0 | 1 |
| H014 | 12 | 15 | 32 | 22 | 11 | 10 | 15 | 17 | - | T | 1 | 0 | 0 | 0 | 0 | 0 | 0 | 0 | 0 | 0 | 0 | 0 | 0 | 0 | 0 | 0 | 0 | 1 |
| H015 | 12 | 15 | 31 | 22 | 12 | 11 | 14 | 13 | - | T | 1 | 0 | 0 | 0 | 0 | 0 | 0 | 0 | 0 | 0 | 0 | 0 | 0 | 0 | 0 | 0 | 0 | 1 |
| H016 | 14 | 12 | 28 | 23 | 11 | 10 | 13 | 15 | + | C | 1 | 0 | 0 | 0 | 0 | 0 | 0 | 0 | 0 | 0 | 0 | 0 | 0 | 0 | 0 | 0 | 0 | 1 |
| H017 | 12 | 13 | 30 | 23 | 11 | 12 | 14 | 14 | + | C | 1 | 0 | 0 | 0 | 0 | 0 | 0 | 0 | 0 | 0 | 0 | 0 | 0 | 0 | 0 | 0 | 0 | 1 |
| H018 | 12 | 14 | 33 | 21 | 11 | 10 | 11 | 15 | + | C | 1 | 0 | 0 | 0 | 0 | 0 | 0 | 0 | 0 | 0 | 0 | 0 | 0 | 0 | 0 | 0 | 0 | 1 |
| H019 | 16 | 13 | 29 | 24 | 11 | 12 | 12 | 14 | - | T | 1 | 0 | 0 | 0 | 0 | 0 | 0 | 0 | 0 | 0 | 0 | 0 | 0 | 0 | 0 | 0 | 0 | 1 |
| H020 | 12 | 14 | 29 | 22 | 11 | 10 | 13 | 15 | + | C | 1 | 0 | 0 | 0 | 0 | 0 | 0 | 0 | 0 | 0 | 0 | 0 | 0 | 0 | 0 | 0 | 0 | 1 |
| H021 | 12 | 13 | 29 | 25 | 11 | 12 | 13 | 14 | - | T | 1 | 0 | 0 | 0 | 0 | 0 | 0 | 0 | 0 | 0 | 0 | 0 | 1 | 0 | 0 | 0 | 0 | 2 |
| H022 | 12 | 13 | 31 | 21 | 10 | 10 | 14 | 15 | + | C | 1 | 0 | 2 | 0 | 0 | 0 | 0 | 0 | 0 | 0 | 0 | 0 | 0 | 0 | 0 | 0 | 0 | 3 |
| H023 | 12 | 13 | 29 | 25 | 12 | 11 | 12 | 13 | - | T | 1 | 0 | 0 | 0 | 0 | 0 | 0 | 0 | 0 | 0 | 0 | 0 | 0 | 0 | 0 | 0 | 0 | 1 |
| H024 | 13 | 11 | 28 | 23 | 11 | 12 | 13 | 15 | + | C | 1 | 0 | 0 | 0 | 0 | 0 | 0 | 0 | 0 | 0 | 0 | 0 | 0 | 0 | 0 | 0 | 0 | 1 |
| H025 | 12 | 14 | 32 | 22 | 11 | 12 | 13 | 15 | + | C | 1 | 0 | 0 | 0 | 0 | 0 | 0 | 0 | 0 | 0 | 0 | 0 | 0 | 0 | 0 | 0 | 0 | 1 |
| H026 | 12 | 15 | 32 | 21 | 11 | 10 | 15 | 13 | + | C | 1 | 0 | 0 | 0 | 0 | 0 | 0 | 0 | 0 | 0 | 0 | 0 | 0 | 0 | 0 | 0 | 0 | 1 |
| H027 | 12 | 12 | 28 | 26 | 11 | 10 | 13 | 14 | + | C | 1 | 0 | 0 | 0 | 0 | 0 | 0 | 0 | 0 | 0 | 0 | 0 | 0 | 0 | 0 | 0 | 0 | 1 |
| H028 | 12 | 14 | 32 | 21 | 12 | 12 | 13 | 14 | - | T | 1 | 0 | 0 | 0 | 0 | 0 | 0 | 0 | 0 | 0 | 0 | 0 | 0 | 0 | 0 | 0 | 0 | 1 |
| H029 | 12 | 13 | 28 | 27 | 11 | 12 | 14 | 14 | + | C | 1 | 0 | 0 | 0 | 0 | 0 | 0 | 0 | 0 | 0 | 0 | 0 | 0 | 0 | 0 | 0 | 0 | 1 |
| H030 | 12 | 14 | 32 | 21 | 11 | 10 | 12 | 14 | + | C | 1 | 0 | 0 | 0 | 0 | 0 | 0 | 0 | 0 | 0 | 0 | 0 | 0 | 0 | 0 | 0 | 0 | 1 |
| H031 | 12 | 14 | 31 | 21 | 11 | 12 | 14 | 15 | + | C | 6 | 0 | 0 | 0 | 0 | 0 | 0 | 0 | 0 | 0 | 0 | 0 | 2 | 0 | 3 | 0 | 0 | 11 |
| H032 | 12 | 14 | 31 | 21 | 11 | 10 | 14 | 17 | + | C | 1 | 0 | 0 | 0 | 0 | 0 | 0 | 0 | 0 | 0 | 0 | 0 | 0 | 0 | 0 | 0 | 0 | 1 |
| H033 | 12 | 13 | 29 | 22 | 12 | 10 | 13 | 14 | + | C | 1 | 0 | 0 | 0 | 0 | 0 | 0 | 0 | 0 | 0 | 0 | 0 | 0 | 0 | 0 | 0 | 0 | 1 |
| H034 | 12 | 14 | 32 | 22 | 11 | 12 | 13 | 14 | + | C | 1 | 0 | 0 | 0 | 0 | 0 | 0 | 0 | 0 | 0 | 0 | 0 | 0 | 0 | 0 | 0 | 0 | 1 |
| H035 | 12 | 15 | 31 | 25 | 11 | 12 | 13 | 13 | - | T | 1 | 0 | 0 | 0 | 0 | 0 | 0 | 0 | 0 | 0 | 0 | 0 | 0 | 0 | 0 | 0 | 0 | 1 |
| H036 | 12 | 13 | 30 | 21 | 12 | 10 | 14 | 15 | + | C | 1 | 0 | 0 | 0 | 0 | 0 | 0 | 0 | 0 | 0 | 0 | 0 | 0 | 0 | 0 | 0 | 0 | 1 |
| H037 | 13 | 15 | 32 | 22 | 11 | 10 | 14 | 17 | + | C | 1 | 0 | 0 | 0 | 0 | 0 | 0 | 0 | 0 | 0 | 0 | 0 | 0 | 0 | 0 | 0 | 0 | 1 |
| H038 | 12 | 13 | 30 | 22 | 11 | 10 | 15 | 14 | + | C | 1 | 0 | 0 | 0 | 0 | 0 | 0 | 0 | 0 | 0 | 0 | 0 | 0 | 0 | 0 | 0 | 0 | 1 |
| H039 | 12 | 14 | 29 | 22 | 11 | 12 | 13 | 15 | + | C | 1 | 0 | 0 | 0 | 0 | 0 | 0 | 0 | 0 | 0 | 0 | 0 | 0 | 0 | 0 | 0 | 0 | 1 |
| H040 | 12 | 14 | 32 | 21 | 11 | 12 | 14 | 15 | + | C | 5 | 0 | 0 | 0 | 0 | 0 | 0 | 0 | 0 | 0 | 0 | 0 | 0 | 0 | 1 | 0 | 0 | 6 |
| H041 | 12 | 14 | 30 | 24 | 12 | 12 | 13 | 14 | - | T | 1 | 0 | 0 | 0 | 0 | 0 | 0 | 0 | 0 | 0 | 0 | 0 | 0 | 0 | 0 | 0 | 0 | 1 |
| H042 | 12 | 12 | 29 | 21 | 11 | 10 | 13 | 15 | + | C | 2 | 0 | 0 | 0 | 0 | 0 | 0 | 0 | 0 | 0 | 0 | 0 | 0 | 0 | 0 | 0 | 0 | 2 |
| H043 | 12 | 13 | 29 | 21 | 12 | 12 | 12 | 13 | - | T | 1 | 0 | 0 | 0 | 0 | 0 | 0 | 0 | 0 | 0 | 0 | 0 | 0 | 0 | 0 | 0 | 0 | 1 |
| H044 | 12 | 14 | 30 | 22 | 11 | 10 | 14 | 16 | + | C | 1 | 0 | 0 | 0 | 0 | 0 | 0 | 0 | 0 | 0 | 0 | 0 | 0 | 0 | 0 | 0 | 0 | 1 |
| H045 | 12 | 13 | 30 | 22 | 11 | 10 | 14 | 14 | + | C | 1 | 0 | 0 | 0 | 0 | 0 | 0 | 0 | 0 | 0 | 0 | 0 | 0 | 0 | 0 | 0 | 0 | 1 |
| H046 | 12 | 12 | 31 | 25 | 12 | 12 | 13 | 12 | - | T | 1 | 0 | 0 | 0 | 0 | 0 | 0 | 0 | 0 | 0 | 0 | 0 | 0 | 0 | 0 | 0 | 0 | 1 |
| H047 | 13 | 15 | 31 | 25 | 11 | 10 | 13 | 15 | - | T | 1 | 0 | 0 | 0 | 0 | 0 | 0 | 0 | 0 | 0 | 0 | 0 | 0 | 0 | 0 | 0 | 0 | 1 |
| H048 | 12 | 14 | 30 | 25 | 12 | 12 | 13 | 13 | - | T | 1 | 0 | 0 | 0 | 0 | 0 | 0 | 0 | 0 | 0 | 0 | 0 | 0 | 0 | 0 | 0 | 0 | 1 |
| H049 | 12 | 13 | 29 | 25 | 11 | 12 | 13 | 13 | - | T | 1 | 0 | 0 | 0 | 0 | 0 | 0 | 0 | 0 | 0 | 0 | 0 | 0 | 0 | 0 | 0 | 0 | 1 |
| H050 | 12 | 13 | 30 | 21 | 11 | 10 | 13 | 16 | + | C | 1 | 0 | 0 | 0 | 0 | 0 | 0 | 0 | 0 | 0 | 0 | 0 | 0 | 1 | 0 | 0 | 0 | 2 |
| H051 | 12 | 15 | 30 | 24 | 11 | 10 | 13 | 15 | - | T | 1 | 0 | 0 | 0 | 0 | 0 | 0 | 0 | 0 | 0 | 0 | 0 | 0 | 0 | 0 | 0 | 0 | 1 |
| H052 | 10 | 13 | 28 | 22 | 12 | 12 | 13 | 15 | - | T | 1 | 0 | 0 | 0 | 0 | 0 | 0 | 0 | 0 | 0 | 0 | 0 | 0 | 0 | 0 | 0 | 0 | 1 |
| H053 | 12 | 13 | 28 | 25 | 11 | 13 | 13 | 15 | - | T | 1 | 0 | 0 | 0 | 0 | 0 | 0 | 0 | 0 | 0 | 0 | 0 | 0 | 0 | 0 | 0 | 0 | 1 |
| H054 | 12 | 14 | 30 | 25 | 11 | 12 | 13 | 13 | - | T | 1 | 0 | 0 | 0 | 0 | 0 | 0 | 0 | 0 | 0 | 0 | 0 | 0 | 0 | 0 | 0 | 0 | 1 |
| H055 | 12 | 15 | 32 | 22 | 11 | 10 | 16 | 16 | + | C | 1 | 0 | 0 | 0 | 0 | 0 | 0 | 0 | 0 | 0 | 0 | 0 | 0 | 0 | 0 | 0 | 0 | 1 |

| | | | | | | | | | | | | | | | | | | | | | | | | | | | | |
|---|---|---|---|---|---|---|---|---|---|---|---|---|---|---|---|---|---|---|---|---|---|---|---|---|---|---|---|---|
| H056 | 12 | 14 | 32 | 25 | 11 | 12 | 13 | 13 | - | T | 1 | 0 | 0 | 0 | 0 | 0 | 0 | 0 | 0 | 0 | 0 | 0 | 0 | 0 | 0 | 0 | 0 | 1 |
| H057 | 12 | 14 | 31 | 24 | 11 | 12 | 13 | 13 | - | T | 1 | 0 | 0 | 0 | 0 | 0 | 0 | 0 | 0 | 0 | 0 | 0 | 0 | 0 | 0 | 0 | 0 | 1 |
| H058 | 12 | 14 | 31 | 22 | 11 | 10 | 13 | 15 | + | C | 1 | 0 | 0 | 0 | 0 | 0 | 0 | 0 | 0 | 0 | 0 | 0 | 0 | 0 | 0 | 0 | 0 | 1 |
| H059 | 12 | 14 | 30 | 25 | 12 | 10 | 14 | 13 | - | T | 1 | 0 | 0 | 0 | 0 | 0 | 0 | 0 | 0 | 0 | 0 | 0 | 0 | 0 | 0 | 0 | 0 | 1 |
| H060 | 12 | 13 | 30 | 21 | 12 | 10 | 14 | 14 | + | C | 1 | 0 | 0 | 0 | 0 | 0 | 0 | 0 | 0 | 0 | 0 | 0 | 0 | 0 | 0 | 0 | 0 | 1 |
| H061 | 12 | 13 | 29 | 25 | 11 | 12 | 12 | 13 | - | T | 1 | 0 | 0 | 0 | 0 | 0 | 0 | 0 | 0 | 0 | 0 | 0 | 0 | 1 | 0 | 0 | 0 | 2 |
| H062 | 12 | 13 | 31 | 21 | 12 | 10 | 12 | 14 | + | C | 1 | 0 | 0 | 0 | 0 | 0 | 0 | 0 | 0 | 0 | 0 | 0 | 0 | 0 | 0 | 0 | 0 | 1 |
| H063 | 12 | 14 | 30 | 24 | 12 | 12 | 12 | 13 | - | T | 1 | 0 | 0 | 0 | 0 | 0 | 0 | 0 | 0 | 0 | 0 | 0 | 0 | 0 | 0 | 0 | 0 | 1 |
| H064 | 12 | 15 | 30 | 25 | 11 | 12 | 14 | 15 | - | C | 1 | 0 | 0 | 0 | 0 | 0 | 0 | 0 | 0 | 0 | 0 | 0 | 0 | 0 | 0 | 0 | 0 | 1 |
| H065 | 12 | 14 | 30 | 23 | 11 | 10 | 13 | 16 | + | C | 1 | 0 | 0 | 0 | 0 | 0 | 0 | 0 | 0 | 0 | 0 | 0 | 0 | 0 | 0 | 0 | 0 | 1 |
| H066 | 12 | 14 | 32 | 22 | 11 | 10 | 13 | 14 | + | C | 1 | 0 | 0 | 0 | 0 | 0 | 0 | 0 | 0 | 0 | 0 | 0 | 0 | 0 | 0 | 0 | 0 | 1 |
| H067 | 12 | 13 | 31 | 22 | 11 | 10 | 12 | 14 | + | C | 1 | 0 | 0 | 0 | 0 | 0 | 0 | 0 | 0 | 0 | 0 | 0 | 0 | 0 | 0 | 0 | 0 | 1 |
| H068 | 12 | 15 | 32 | 22 | 11 | 10 | 15 | 16 | + | C | 1 | 0 | 0 | 0 | 0 | 0 | 0 | 0 | 0 | 0 | 0 | 0 | 0 | 0 | 0 | 0 | 0 | 1 |
| H069 | 12 | 14 | 31 | 22 | 11 | 10 | 15 | 17 | + | C | 1 | 0 | 0 | 0 | 0 | 0 | 0 | 0 | 0 | 0 | 0 | 0 | 0 | 0 | 0 | 0 | 0 | 1 |
| H070 | 12 | 14 | 32 | 22 | 11 | 12 | 13 | 16 | + | C | 1 | 0 | 0 | 0 | 0 | 0 | 0 | 0 | 0 | 0 | 0 | 0 | 0 | 0 | 0 | 0 | 0 | 1 |
| H071 | 13 | 15 | 31 | 23 | 11 | 10 | 13 | 16 | + | C | 1 | 0 | 0 | 0 | 0 | 0 | 0 | 0 | 0 | 0 | 0 | 0 | 0 | 0 | 0 | 0 | 0 | 1 |
| H072 | 12 | 13 | 30 | 21 | 11 | 12 | 13 | 14 | - | T | 1 | 0 | 0 | 0 | 0 | 0 | 0 | 0 | 0 | 0 | 0 | 0 | 0 | 0 | 0 | 0 | 0 | 1 |
| H073 | 12 | 15 | 30 | 21 | 12 | 10 | 13 | 13 | + | C | 1 | 0 | 0 | 0 | 0 | 0 | 0 | 0 | 0 | 0 | 0 | 0 | 0 | 0 | 0 | 0 | 0 | 1 |
| H074 | 12 | 14 | 30 | 21 | 11 | 12 | 14 | 13 | - | T | 1 | 0 | 0 | 0 | 0 | 0 | 0 | 0 | 0 | 0 | 0 | 0 | 0 | 0 | 0 | 0 | 0 | 1 |
| H075 | 12 | 14 | 30 | 25 | 12 | 10 | 13 | 16 | - | C | 1 | 0 | 0 | 0 | 0 | 0 | 0 | 0 | 0 | 0 | 0 | 0 | 0 | 0 | 0 | 0 | 0 | 1 |
| H076 | 12 | 14 | 32 | 22 | 11 | 10 | 12 | 15 | + | C | 1 | 0 | 0 | 0 | 0 | 0 | 0 | 0 | 0 | 0 | 0 | 0 | 0 | 0 | 0 | 0 | 0 | 1 |
| H077 | 12 | 14 | 31 | 21 | 11 | 12 | 13 | 17 | + | C | 3 | 0 | 0 | 0 | 0 | 0 | 0 | 1 | 0 | 0 | 0 | 0 | 1 | 0 | 1 | 0 | 2 | 8 |
| H078 | 12 | 14 | 31 | 25 | 12 | 12 | 12 | 15 | - | T | 1 | 0 | 0 | 0 | 0 | 0 | 0 | 0 | 0 | 0 | 0 | 0 | 0 | 0 | 0 | 0 | 0 | 1 |
| H079 | 12 | 14 | 33 | 21 | 12 | 12 | 12 | 13 | + | C | 1 | 0 | 0 | 0 | 0 | 0 | 0 | 0 | 0 | 0 | 0 | 0 | 0 | 0 | 0 | 0 | 0 | 1 |
| H080 | 12 | 13 | 30 | 21 | 11 | 12 | 13 | 17 | + | C | 4 | 0 | 0 | 0 | 0 | 1 | 0 | 2 | 0 | 0 | 3 | 0 | 0 | 0 | 0 | 0 | 5 | 15 |
| H081 | 14 | 12 | 28 | 22 | 11 | 12 | 12 | 14 | - | T | 2 | 0 | 0 | 0 | 0 | 0 | 0 | 0 | 0 | 0 | 0 | 0 | 0 | 0 | 0 | 0 | 0 | 2 |
| H082 | 12 | 12 | 28 | 25 | 12 | 12 | 12 | 14 | + | C | 1 | 0 | 0 | 0 | 0 | 0 | 0 | 0 | 0 | 0 | 0 | 0 | 0 | 0 | 0 | 0 | 0 | 1 |
| H083 | 12 | 13 | 30 | 21 | 11 | 10 | 13 | 17 | + | C | 2 | 0 | 0 | 0 | 0 | 0 | 0 | 0 | 0 | 0 | 0 | 0 | 0 | 0 | 0 | 0 | 0 | 2 |
| H084 | 16 | 13 | 29 | 24 | 11 | 12 | 12 | 16 | - | T | 1 | 0 | 0 | 0 | 0 | 0 | 0 | 0 | 0 | 0 | 0 | 0 | 0 | 0 | 0 | 0 | 0 | 1 |
| H085 | 13 | 13 | 31 | 21 | 11 | 10 | 12 | 16 | + | C | 1 | 0 | 0 | 0 | 0 | 0 | 0 | 0 | 0 | 0 | 0 | 0 | 0 | 0 | 0 | 0 | 0 | 1 |
| H086 | 12 | 14 | 32 | 21 | 11 | 12 | 12 | 15 | + | C | 4 | 0 | 0 | 0 | 0 | 0 | 0 | 0 | 0 | 0 | 0 | 0 | 1 | 0 | 0 | 0 | 1 | 6 |
| H087 | 13 | 13 | 30 | 21 | 11 | 10 | 13 | 15 | + | C | 1 | 0 | 0 | 0 | 0 | 0 | 0 | 0 | 0 | 0 | 0 | 0 | 0 | 0 | 0 | 0 | 0 | 1 |
| H088 | 12 | 13 | 30 | 21 | 11 | 12 | 14 | 17 | + | C | 5 | 0 | 0 | 1 | 0 | 1 | 1 | 1 | 0 | 0 | 0 | 0 | 0 | 0 | 0 | 0 | 1 | 10 |
| H089 | 12 | 13 | 31 | 21 | 11 | 12 | 13 | 16 | + | C | 2 | 0 | 0 | 0 | 0 | 0 | 0 | 0 | 0 | 0 | 0 | 1 | 0 | 0 | 0 | 0 | 1 | 4 |
| H090 | 12 | 13 | 30 | 21 | 11 | 12 | 14 | 15 | + | C | 2 | 0 | 0 | 0 | 0 | 0 | 0 | 0 | 1 | 0 | 0 | 0 | 0 | 0 | 0 | 0 | 3 | 6 |
| H091 | 13 | 13 | 30 | 22 | 11 | 12 | 12 | 15 | + | C | 1 | 0 | 0 | 0 | 0 | 0 | 0 | 0 | 0 | 0 | 0 | 0 | 0 | 0 | 0 | 0 | 0 | 1 |
| H092 | 16 | 13 | 29 | 23 | 10 | 12 | 11 | 15 | - | T | 1 | 0 | 0 | 0 | 0 | 0 | 0 | 0 | 0 | 0 | 0 | 0 | 0 | 0 | 0 | 0 | 0 | 1 |
| H093 | 13 | 14 | 30 | 24 | 11 | 13 | 14 | 15 | - | T | 1 | 0 | 0 | 0 | 0 | 0 | 0 | 0 | 0 | 0 | 0 | 0 | 0 | 0 | 0 | 0 | 0 | 1 |
| H094 | 10 | 14 | 32 | 25 | 11 | 12 | 12 | 15 | - | T | 1 | 0 | 0 | 0 | 0 | 0 | 0 | 0 | 0 | 0 | 0 | 0 | 0 | 0 | 0 | 0 | 0 | 1 |
| H095 | 12 | 13 | 30 | 21 | 11 | 11 | 13 | 17 | + | C | 1 | 0 | 0 | 0 | 0 | 1 | 0 | 0 | 0 | 0 | 0 | 0 | 0 | 0 | 0 | 0 | 0 | 2 |
| H096 | 12 | 13 | 31 | 21 | 11 | 12 | 12 | 15 | + | C | 7 | 0 | 0 | 2 | 0 | 1 | 1 | 2 | 0 | 0 | 4 | 0 | 0 | 0 | 0 | 0 | 3 | 20 |
| H097 | 12 | 13 | 30 | 21 | 11 | 12 | 12 | 15 | + | C | 4 | 0 | 0 | 0 | 0 | 0 | 0 | 3 | 0 | 0 | 2 | 0 | 0 | 0 | 0 | 0 | 1 | 10 |
| H098 | 13 | 12 | 28 | 21 | 11 | 12 | 12 | 14 | - | T | 1 | 0 | 0 | 0 | 0 | 0 | 0 | 0 | 0 | 0 | 0 | 0 | 0 | 0 | 0 | 0 | 0 | 1 |
| H099 | 12 | 13 | 30 | 24 | 12 | 14 | 12 | 14 | - | T | 2 | 0 | 0 | 0 | 0 | 0 | 0 | 0 | 0 | 0 | 0 | 0 | 0 | 0 | 0 | 0 | 0 | 2 |
| H100 | 12 | 13 | 31 | 21 | 11 | 12 | 12 | 14 | + | C | 1 | 0 | 0 | 0 | 0 | 1 | 0 | 0 | 0 | 0 | 0 | 0 | 0 | 0 | 0 | 0 | 0 | 2 |
| H101 | 12 | 13 | 32 | 21 | 11 | 11 | 12 | 14 | + | C | 1 | 0 | 0 | 0 | 0 | 0 | 0 | 0 | 0 | 0 | 0 | 0 | 0 | 0 | 0 | 0 | 0 | 1 |
| H102 | 10 | 14 | 32 | 25 | 10 | 11 | 12 | 15 | - | T | 1 | 0 | 0 | 0 | 0 | 0 | 0 | 0 | 0 | 0 | 0 | 0 | 0 | 0 | 0 | 0 | 0 | 1 |
| H103 | 12 | 12 | 30 | 21 | 11 | 11 | 14 | 15 | + | C | 1 | 0 | 0 | 0 | 0 | 0 | 0 | 0 | 0 | 0 | 0 | 0 | 0 | 0 | 0 | 0 | 0 | 1 |
| H104 | 12 | 13 | 29 | 21 | 11 | 12 | 13 | 16 | + | C | 1 | 0 | 0 | 0 | 0 | 0 | 0 | 0 | 0 | 0 | 0 | 0 | 0 | 0 | 0 | 0 | 0 | 1 |
| H105 | 13 | 14 | 30 | 25 | 12 | 12 | 12 | 14 | - | C | 1 | 0 | 0 | 0 | 0 | 0 | 0 | 0 | 0 | 0 | 0 | 0 | 0 | 0 | 0 | 0 | 0 | 1 |
| H106 | 12 | 13 | 30 | 21 | 11 | 14 | 12 | 15 | + | C | 1 | 0 | 0 | 0 | 0 | 0 | 0 | 0 | 0 | 0 | 0 | 0 | 0 | 0 | 0 | 0 | 0 | 1 |
| H107 | 12 | 13 | 30 | 21 | 11 | 10 | 14 | 16 | + | C | 1 | 0 | 1 | 0 | 0 | 0 | 0 | 0 | 0 | 0 | 0 | 0 | 0 | 1 | 0 | 0 | 0 | 3 |
| H108 | 12 | 12 | 28 | 23 | 11 | 12 | 12 | 14 | - | T | 1 | 0 | 0 | 0 | 0 | 0 | 0 | 0 | 0 | 0 | 0 | 0 | 0 | 0 | 0 | 0 | 0 | 1 |
| H109 | 13 | 14 | 32 | 21 | 11 | 10 | 12 | 15 | + | C | 1 | 0 | 0 | 0 | 0 | 0 | 0 | 0 | 0 | 0 | 0 | 0 | 0 | 0 | 0 | 0 | 0 | 1 |
| H110 | 12 | 13 | 29 | 25 | 11 | 10 | 12 | 14 | - | T | 1 | 0 | 0 | 0 | 0 | 0 | 0 | 0 | 0 | 0 | 0 | 0 | 0 | 0 | 0 | 0 | 0 | 1 |
| H111 | 12 | 13 | 29 | 25 | 12 | 12 | 12 | 14 | - | C | 1 | 0 | 0 | 0 | 0 | 0 | 0 | 0 | 0 | 0 | 0 | 0 | 0 | 0 | 0 | 0 | 0 | 1 |
| H112 | 14 | 12 | 28 | 23 | 11 | 12 | 12 | 14 | - | T | 2 | 0 | 0 | 0 | 0 | 0 | 0 | 0 | 0 | 0 | 0 | 0 | 0 | 0 | 0 | 0 | 0 | 2 |
| H113 | 12 | 13 | 29 | 25 | 11 | 14 | 12 | 14 | - | T | 1 | 0 | 0 | 0 | 1 | 0 | 0 | 0 | 0 | 0 | 0 | 0 | 0 | 0 | 0 | 0 | 0 | 2 |
| H114 | 12 | 13 | 29 | 25 | 12 | 14 | 12 | 14 | - | T | 5 | 0 | 0 | 0 | 0 | 0 | 0 | 0 | 0 | 0 | 0 | 0 | 0 | 0 | 0 | 0 | 0 | 5 |

| | | | | | | | | | | | | | | | | | | | | | | | | | | | | |
|---|---|---|---|---|---|---|---|---|---|---|---|---|---|---|---|---|---|---|---|---|---|---|---|---|---|---|---|---|
| H115 | 12 | 13 | 28 | 24 | 12 | 14 | 12 | 14 | - | T | 1 | 0 | 0 | 0 | 0 | 0 | 0 | 0 | 0 | 0 | 0 | 0 | 0 | 0 | 0 | 0 | 0 | 1 |
| H116 | 12 | 14 | 31 | 24 | 11 | 12 | 12 | 13 | + | C | 1 | 0 | 0 | 0 | 0 | 0 | 0 | 0 | 0 | 0 | 0 | 0 | 0 | 0 | 0 | 0 | 0 | 1 |
| H117 | 12 | 13 | 30 | 23 | 11 | 13 | 13 | 15 | - | T | 1 | 0 | 0 | 0 | 0 | 0 | 0 | 0 | 0 | 0 | 0 | 0 | 0 | 0 | 0 | 0 | 0 | 1 |
| H118 | 13 | 13 | 29 | 24 | 11 | 13 | 13 | 14 | - | T | 1 | 0 | 0 | 0 | 0 | 0 | 0 | 0 | 0 | 0 | 0 | 0 | 0 | 0 | 0 | 0 | 0 | 1 |
| H119 | 12 | 14 | 31 | 21 | 11 | 12 | 13 | 16 | + | C | 3 | 0 | 0 | 1 | 0 | 1 | 0 | 0 | 1 | 0 | 0 | 0 | 0 | 0 | 0 | 0 | 1 | 7 |
| H120 | 12 | 13 | 30 | 21 | 11 | 12 | 14 | 16 | + | C | 5 | 0 | 0 | 1 | 0 | 0 | 0 | 1 | 0 | 0 | 0 | 0 | 0 | 0 | 0 | 0 | 5 | 12 |
| H121 | 12 | 13 | 30 | 22 | 12 | 10 | 14 | 15 | + | C | 1 | 0 | 1 | 0 | 0 | 0 | 0 | 0 | 0 | 0 | 0 | 0 | 0 | 0 | 0 | 0 | 0 | 2 |
| H122 | 13 | 13 | 32 | 21 | 11 | 10 | 12 | 15 | + | C | 1 | 0 | 0 | 0 | 0 | 0 | 0 | 0 | 0 | 0 | 0 | 0 | 0 | 0 | 0 | 0 | 0 | 1 |
| H123 | 13 | 13 | 30 | 21 | 11 | 12 | 12 | 15 | + | C | 1 | 0 | 0 | 0 | 0 | 0 | 0 | 0 | 0 | 0 | 0 | 0 | 0 | 0 | 0 | 0 | 0 | 1 |
| H124 | 12 | 13 | 30 | 24 | 12 | 12 | 12 | 14 | - | T | 1 | 0 | 0 | 0 | 1 | 0 | 0 | 0 | 0 | 0 | 0 | 0 | 0 | 0 | 0 | 0 | 0 | 2 |
| H125 | 12 | 13 | 30 | 21 | 11 | 12 | 13 | 16 | + | C | 4 | 0 | 0 | 1 | 0 | 0 | 1 | 1 | 0 | 0 | 2 | 0 | 0 | 0 | 0 | 2 | 3 | 14 |
| H126 | 13 | 13 | 30 | 21 | 11 | 12 | 14 | 15 | + | C | 1 | 0 | 0 | 0 | 0 | 0 | 0 | 0 | 0 | 0 | 0 | 0 | 0 | 0 | 0 | 0 | 0 | 1 |
| H127 | 12 | 12 | 29 | 21 | 11 | 12 | 13 | 16 | + | C | 2 | 0 | 0 | 0 | 0 | 1 | 0 | 0 | 0 | 0 | 0 | 0 | 0 | 0 | 0 | 0 | 0 | 3 |
| H128 | 12 | 14 | 30 | 21 | 11 | 12 | 13 | 15 | + | C | 3 | 0 | 0 | 0 | 0 | 0 | 0 | 0 | 0 | 0 | 0 | 0 | 0 | 0 | 1 | 0 | 0 | 4 |
| H129 | 12 | 13 | 31 | 24 | 12 | 14 | 12 | 14 | - | T | 1 | 0 | 0 | 0 | 0 | 0 | 0 | 0 | 0 | 0 | 0 | 0 | 0 | 0 | 0 | 0 | 0 | 1 |
| H130 | 12 | 13 | 29 | 24 | 11 | 15 | 12 | 14 | - | T | 1 | 0 | 0 | 0 | 0 | 0 | 0 | 0 | 0 | 0 | 0 | 0 | 0 | 0 | 0 | 0 | 0 | 1 |
| H131 | 12 | 13 | 31 | 21 | 11 | 12 | 14 | 15 | + | C | 3 | 0 | 0 | 0 | 0 | 0 | 0 | 0 | 0 | 0 | 1 | 0 | 0 | 0 | 0 | 1 | 0 | 5 |
| H132 | 12 | 14 | 30 | 20 | 11 | 12 | 13 | 15 | + | C | 1 | 0 | 0 | 0 | 0 | 0 | 0 | 0 | 0 | 0 | 0 | 0 | 0 | 0 | 0 | 0 | 0 | 1 |
| H133 | 12 | 13 | 29 | 24 | 14 | 14 | 12 | 14 | - | T | 1 | 0 | 0 | 0 | 0 | 0 | 0 | 0 | 0 | 0 | 0 | 0 | 0 | 0 | 0 | 0 | 0 | 1 |
| H134 | 13 | 15 | 32 | 24 | 11 | 14 | 11 | 15 | - | C | 1 | 0 | 0 | 0 | 0 | 0 | 0 | 0 | 0 | 0 | 0 | 0 | 0 | 0 | 0 | 0 | 0 | 1 |
| H135 | 12 | 13 | 29 | 24 | 11 | 13 | 12 | 15 | - | T | 1 | 0 | 0 | 0 | 0 | 0 | 0 | 0 | 0 | 0 | 0 | 0 | 0 | 0 | 0 | 0 | 0 | 1 |
| H136 | 12 | 14 | 30 | 25 | 11 | 12 | 12 | 14 | - | T | 1 | 0 | 0 | 0 | 0 | 0 | 0 | 0 | 0 | 0 | 0 | 0 | 0 | 0 | 0 | 0 | 0 | 1 |
| H137 | 12 | 14 | 31 | 21 | 12 | 12 | 13 | 15 | + | C | 1 | 0 | 0 | 0 | 0 | 0 | 0 | 0 | 0 | 0 | 0 | 0 | 0 | 0 | 0 | 0 | 0 | 1 |
| H138 | 15 | 14 | 30 | 23 | 11 | 12 | 12 | 15 | - | T | 1 | 0 | 0 | 0 | 0 | 0 | 0 | 0 | 0 | 0 | 0 | 0 | 0 | 0 | 0 | 0 | 0 | 1 |
| H139 | 12 | 14 | 31 | 24 | 11 | 12 | 12 | 14 | - | T | 1 | 0 | 0 | 0 | 1 | 0 | 0 | 0 | 0 | 1 | 0 | 0 | 0 | 0 | 0 | 0 | 0 | 3 |
| H140 | 13 | 14 | 30 | 22 | 12 | 12 | 12 | 16 | - | C | 1 | 0 | 0 | 0 | 0 | 0 | 0 | 0 | 0 | 0 | 0 | 0 | 0 | 0 | 0 | 0 | 0 | 1 |
| H141 | 12 | 13 | 29 | 21 | 11 | 12 | 13 | 15 | + | C | 1 | 0 | 0 | 0 | 0 | 0 | 0 | 0 | 0 | 0 | 1 | 0 | 0 | 0 | 0 | 0 | 0 | 2 |
| H142 | 12 | 13 | 30 | 21 | 11 | 12 | 13 | 15 | + | C | 7 | 0 | 0 | 0 | 0 | 1 | 1 | 1 | 0 | 0 | 2 | 2 | 1 | 0 | 0 | 0 | 2 | 17 |
| H143 | 12 | 14 | 31 | 24 | 11 | 14 | 12 | 15 | - | T | 1 | 0 | 0 | 0 | 0 | 0 | 0 | 0 | 0 | 0 | 0 | 0 | 0 | 0 | 0 | 0 | 0 | 1 |
| H144 | 12 | 14 | 31 | 21 | 11 | 12 | 12 | 15 | + | C | 1 | 0 | 0 | 1 | 0 | 0 | 0 | 2 | 0 | 0 | 1 | 1 | 0 | 0 | 0 | 0 | 0 | 6 |
| H145 | 12 | 13 | 30 | 23 | 11 | 12 | 12 | 15 | - | T | 1 | 0 | 0 | 0 | 1 | 0 | 0 | 0 | 0 | 0 | 0 | 0 | 0 | 0 | 0 | 0 | 0 | 2 |
| H146 | 14 | 12 | 29 | 22 | 11 | 12 | 13 | 15 | - | T | 1 | 0 | 0 | 0 | 0 | 0 | 0 | 0 | 0 | 0 | 0 | 0 | 0 | 0 | 0 | 0 | 0 | 1 |
| H147 | 12 | 14 | 30 | 23 | 12 | 14 | 13 | 14 | - | C | 1 | 0 | 0 | 0 | 0 | 0 | 0 | 0 | 0 | 0 | 0 | 0 | 0 | 0 | 0 | 0 | 0 | 1 |
| H148 | 12 | 14 | 31 | 24 | 11 | 14 | 13 | 14 | - | T | 1 | 0 | 0 | 0 | 0 | 0 | 0 | 0 | 0 | 0 | 0 | 0 | 0 | 0 | 0 | 0 | 0 | 1 |
| H149 | 10 | 12 | 29 | 22 | 12 | 12 | 14 | 14 | - | T | 1 | 0 | 0 | 0 | 0 | 0 | 0 | 0 | 0 | 0 | 0 | 0 | 0 | 0 | 0 | 0 | 0 | 1 |
| H150 | 12 | 13 | 31 | 21 | 11 | 12 | 13 | 15 | + | C | 4 | 0 | 0 | 1 | 0 | 0 | 0 | 0 | 1 | 0 | 1 | 0 | 0 | 0 | 1 | 0 | 1 | 9 |
| H151 | 12 | 14 | 30 | 25 | 12 | 13 | 13 | 14 | - | C | 1 | 0 | 0 | 0 | 0 | 0 | 0 | 0 | 0 | 0 | 0 | 0 | 0 | 0 | 0 | 0 | 0 | 1 |
| H152 | 12 | 15 | 32 | 21 | 11 | 12 | 15 | 17 | - | T | 1 | 0 | 0 | 0 | 0 | 0 | 0 | 0 | 0 | 0 | 0 | 0 | 0 | 0 | 0 | 0 | 0 | 1 |
| H153 | 12 | 13 | 29 | 21 | 10 | 12 | 13 | 14 | - | C | 1 | 0 | 0 | 0 | 0 | 0 | 0 | 0 | 0 | 0 | 0 | 0 | 0 | 0 | 0 | 0 | 0 | 1 |
| H154 | 12 | 13 | 30 | 21 | 11 | 12 | 14 | 15 | - | T | 1 | 0 | 0 | 0 | 0 | 0 | 0 | 0 | 0 | 0 | 0 | 0 | 0 | 0 | 0 | 0 | 0 | 1 |
| H155 | 13 | 15 | 31 | 24 | 12 | 12 | 12 | 13 | + | C | 1 | 0 | 0 | 0 | 0 | 0 | 0 | 0 | 0 | 0 | 0 | 0 | 0 | 0 | 0 | 0 | 0 | 1 |
| H156 | 12 | 13 | 29 | 21 | 11 | 12 | 14 | 15 | + | C | 1 | 0 | 0 | 0 | 0 | 1 | 0 | 0 | 0 | 0 | 0 | 0 | 0 | 0 | 0 | 0 | 0 | 2 |
| H157 | 13 | 11 | 27 | 24 | 11 | 14 | 13 | 14 | - | C | 1 | 0 | 0 | 0 | 0 | 0 | 0 | 0 | 0 | 0 | 0 | 0 | 0 | 0 | 0 | 0 | 0 | 1 |
| H158 | 12 | 12 | 29 | 22 | 11 | 12 | 14 | 17 | + | C | 1 | 0 | 0 | 0 | 0 | 0 | 0 | 0 | 0 | 0 | 0 | 0 | 0 | 0 | 0 | 0 | 0 | 1 |
| H159 | 12 | 12 | 29 | 21 | 11 | 12 | 14 | 15 | + | C | 1 | 0 | 0 | 0 | 0 | 0 | 0 | 0 | 0 | 0 | 0 | 0 | 0 | 0 | 2 | 0 | 0 | 3 |
| H160 | 13 | 14 | 29 | 23 | 11 | 12 | 12 | 15 | - | T | 1 | 0 | 0 | 0 | 0 | 0 | 0 | 0 | 0 | 0 | 0 | 0 | 0 | 0 | 0 | 0 | 0 | 1 |
| H161 | 12 | 12 | 28 | 24 | 12 | 12 | 12 | 14 | + | C | 1 | 0 | 0 | 0 | 0 | 0 | 0 | 0 | 0 | 0 | 0 | 0 | 0 | 0 | 0 | 0 | 0 | 1 |
| H162 | 14 | 13 | 30 | 21 | 11 | 12 | 14 | 15 | + | C | 1 | 0 | 0 | 0 | 0 | 0 | 0 | 0 | 0 | 0 | 0 | 0 | 0 | 0 | 0 | 0 | 0 | 1 |
| H163 | 13 | 13 | 30 | 25 | 10 | 13 | 13 | 13 | + | C | 1 | 0 | 0 | 0 | 0 | 0 | 0 | 0 | 0 | 0 | 0 | 0 | 0 | 0 | 0 | 0 | 0 | 1 |
| H164 | 12 | 14 | 31 | 21 | 11 | 11 | 13 | 15 | + | C | 2 | 0 | 0 | 0 | 0 | 1 | 0 | 0 | 0 | 0 | 0 | 0 | 1 | 0 | 0 | 0 | 0 | 4 |
| H165 | 12 | 15 | 31 | 21 | 11 | 12 | 14 | 16 | + | C | 1 | 0 | 0 | 0 | 0 | 0 | 0 | 0 | 0 | 0 | 0 | 0 | 0 | 0 | 0 | 0 | 0 | 1 |
| H166 | 13 | 12 | 29 | 22 | 12 | 12 | 13 | 15 | + | C | 1 | 0 | 0 | 0 | 0 | 0 | 0 | 0 | 0 | 0 | 0 | 0 | 0 | 0 | 0 | 0 | 0 | 1 |
| H167 | 12 | 14 | 31 | 21 | 11 | 12 | 14 | 17 | + | C | 2 | 0 | 0 | 1 | 0 | 0 | 0 | 0 | 0 | 0 | 0 | 0 | 0 | 0 | 0 | 2 | 1 | 6 |
| H168 | 12 | 12 | 31 | 21 | 11 | 12 | 14 | 15 | + | C | 2 | 0 | 0 | 0 | 0 | 0 | 0 | 0 | 0 | 0 | 0 | 0 | 0 | 0 | 0 | 0 | 0 | 2 |
| H169 | 12 | 13 | 32 | 21 | 11 | 12 | 13 | 15 | + | C | 2 | 0 | 0 | 2 | 0 | 0 | 0 | 1 | 0 | 0 | 0 | 0 | 0 | 0 | 0 | 1 | 0 | 6 |
| H170 | 12 | 12 | 30 | 22 | 12 | 12 | 13 | 15 | + | C | 1 | 0 | 0 | 0 | 0 | 0 | 0 | 0 | 0 | 0 | 0 | 0 | 0 | 0 | 0 | 0 | 0 | 1 |
| H171 | 12 | 14 | 31 | 21 | 11 | 12 | 13 | 15 | + | C | 2 | 0 | 0 | 0 | 0 | 0 | 1 | 0 | 1 | 0 | 1 | 1 | 2 | 0 | 0 | 0 | 0 | 8 |
| H172 | 12 | 13 | 31 | 21 | 11 | 12 | 14 | 16 | + | C | 1 | 0 | 0 | 0 | 0 | 0 | 0 | 1 | 0 | 0 | 0 | 0 | 0 | 0 | 0 | 0 | 0 | 2 |
| H173 | 12 | 15 | 31 | 21 | 11 | 12 | 14 | 15 | + | C | 1 | 0 | 0 | 0 | 0 | 0 | 0 | 0 | 0 | 0 | 0 | 0 | 0 | 0 | 0 | 0 | 0 | 1 |

| | | | | | | | | | | | | | | | | | | | | | | | | | | | | |
|---|---|---|---|---|---|---|---|---|---|---|---|---|---|---|---|---|---|---|---|---|---|---|---|---|---|---|---|---|
| H174 | 12 | 15 | 33 | 21 | 11 | 12 | 13 | 15 | + | C | 2 | 0 | 0 | 0 | 0 | 0 | 0 | 0 | 0 | 0 | 0 | 0 | 0 | 0 | 0 | 0 | 0 | 2 |
| H175 | 12 | 15 | 30 | 21 | 11 | 12 | 12 | 16 | + | C | 1 | 0 | 0 | 0 | 0 | 0 | 0 | 0 | 0 | 0 | 0 | 0 | 0 | 0 | 0 | 0 | 0 | 1 |
| H176 | 12 | 14 | 32 | 22 | 10 | 12 | 14 | 16 | + | C | 1 | 0 | 0 | 0 | 0 | 0 | 0 | 0 | 0 | 0 | 0 | 0 | 0 | 0 | 0 | 0 | 0 | 1 |
| H177 | 12 | 14 | 30 | 25 | 12 | 14 | 13 | 14 | - | T | 2 | 0 | 0 | 0 | 0 | 0 | 0 | 0 | 0 | 0 | 0 | 0 | 0 | 0 | 0 | 0 | 0 | 2 |
| H178 | 12 | 13 | 30 | 20 | 11 | 12 | 15 | 17 | + | C | 1 | 0 | 0 | 0 | 0 | 0 | 0 | 0 | 0 | 0 | 0 | 0 | 0 | 0 | 0 | 0 | 0 | 1 |
| H179 | 12 | 13 | 28 | 24 | 11 | 14 | 13 | 14 | - | T | 1 | 0 | 0 | 0 | 0 | 0 | 0 | 0 | 0 | 0 | 0 | 0 | 0 | 0 | 0 | 0 | 0 | 1 |
| H180 | 14 | 13 | 29 | 24 | 11 | 11 | 13 | 14 | - | T | 1 | 0 | 0 | 0 | 0 | 0 | 0 | 0 | 0 | 0 | 0 | 0 | 0 | 0 | 0 | 0 | 0 | 1 |
| H181 | 12 | 14 | 32 | 21 | 11 | 12 | 14 | 16 | + | C | 2 | 0 | 0 | 0 | 0 | 0 | 0 | 0 | 0 | 0 | 0 | 0 | 1 | 0 | 0 | 0 | 0 | 3 |
| H182 | 12 | 13 | 29 | 21 | 11 | 12 | 15 | 16 | + | C | 1 | 0 | 0 | 0 | 0 | 0 | 0 | 0 | 0 | 0 | 0 | 0 | 0 | 0 | 0 | 0 | 0 | 1 |
| H183 | 11 | 14 | 30 | 21 | 11 | 12 | 14 | 15 | - | C | 1 | 0 | 0 | 0 | 0 | 0 | 0 | 0 | 0 | 0 | 0 | 0 | 0 | 0 | 0 | 0 | 0 | 1 |
| H184 | 11 | 14 | 31 | 21 | 11 | 12 | 13 | 17 | + | C | 1 | 0 | 0 | 0 | 0 | 0 | 0 | 0 | 0 | 0 | 0 | 0 | 0 | 0 | 0 | 0 | 0 | 1 |
| H185 | 11 | 13 | 30 | 21 | 10 | 12 | 13 | 15 | + | C | 1 | 0 | 0 | 0 | 0 | 0 | 0 | 0 | 0 | 0 | 0 | 0 | 0 | 0 | 0 | 0 | 0 | 1 |
| H186 | 11 | 13 | 29 | 21 | 11 | 12 | 13 | 16 | + | C | 1 | 0 | 0 | 0 | 0 | 0 | 0 | 0 | 0 | 0 | 0 | 0 | 0 | 0 | 0 | 0 | 0 | 1 |
| H187 | 11 | 13 | 29 | 24 | 12 | 13 | 12 | 14 | - | T | 1 | 0 | 0 | 0 | 0 | 0 | 0 | 0 | 0 | 0 | 0 | 0 | 0 | 0 | 0 | 0 | 0 | 1 |
| H188 | 11 | 13 | 29 | 23 | 11 | 13 | 12 | 14 | - | T | 1 | 0 | 0 | 0 | 0 | 0 | 0 | 0 | 0 | 0 | 0 | 0 | 0 | 0 | 0 | 0 | 0 | 1 |
| H189 | 11 | 12 | 29 | 22 | 11 | 10 | 12 | 15 | + | C | 1 | 0 | 0 | 0 | 0 | 0 | 0 | 0 | 0 | 0 | 0 | 0 | 0 | 0 | 0 | 0 | 0 | 1 |
| H190 | 11 | 13 | 29 | 24 | 11 | 13 | 12 | 14 | - | T | 2 | 0 | 0 | 0 | 0 | 0 | 0 | 0 | 0 | 0 | 0 | 0 | 0 | 0 | 0 | 0 | 0 | 2 |
| H191 | 11 | 13 | 29 | 24 | 12 | 12 | 12 | 14 | - | T | 1 | 0 | 0 | 0 | 0 | 0 | 0 | 0 | 0 | 0 | 0 | 0 | 0 | 0 | 0 | 0 | 0 | 1 |
| H192 | 12 | 13 | 31 | 21 | 10 | 10 | 12 | 16 | + | C | 1 | 0 | 0 | 0 | 0 | 0 | 0 | 0 | 0 | 0 | 0 | 0 | 0 | 0 | 0 | 0 | 0 | 1 |
| H193 | 13 | 12 | 28 | 23 | 11 | 10 | 12 | 15 | - | T | 1 | 0 | 0 | 0 | 0 | 0 | 0 | 0 | 0 | 0 | 0 | 0 | 0 | 0 | 0 | 0 | 0 | 1 |
| H194 | 11 | 13 | 29 | 23 | 12 | 13 | 12 | 14 | - | T | 1 | 0 | 0 | 0 | 0 | 0 | 0 | 0 | 0 | 0 | 0 | 0 | 0 | 0 | 0 | 0 | 0 | 1 |
| H195 | 11 | 13 | 29 | 23 | 12 | 14 | 12 | 14 | - | T | 1 | 0 | 0 | 0 | 0 | 0 | 0 | 0 | 0 | 0 | 0 | 0 | 0 | 0 | 0 | 0 | 0 | 1 |
| H196 | 14 | 13 | 30 | 23 | 11 | 12 | 11 | 14 | - | T | 1 | 0 | 0 | 0 | 0 | 0 | 0 | 0 | 0 | 0 | 0 | 0 | 0 | 0 | 0 | 0 | 0 | 1 |
| H197 | 12 | 14 | 30 | 23 | 10 | 10 | 11 | 14 | - | T | 1 | 0 | 0 | 0 | 0 | 0 | 0 | 0 | 0 | 0 | 0 | 0 | 0 | 0 | 0 | 0 | 0 | 1 |
| H198 | 11 | 13 | 30 | 21 | 10 | 10 | 12 | 17 | + | C | 1 | 0 | 0 | 0 | 0 | 0 | 0 | 0 | 0 | 0 | 0 | 0 | 0 | 0 | 0 | 0 | 0 | 1 |
| H199 | 11 | 13 | 30 | 22 | 11 | 12 | 12 | 14 | - | T | 1 | 0 | 0 | 0 | 0 | 0 | 0 | 0 | 0 | 0 | 0 | 0 | 0 | 0 | 0 | 0 | 0 | 1 |
| H200 | 11 | 13 | 30 | 24 | 10 | 12 | 12 | 14 | - | T | 1 | 0 | 0 | 0 | 0 | 0 | 0 | 0 | 0 | 0 | 0 | 0 | 0 | 0 | 0 | 0 | 0 | 1 |
| H201 | 12 | 12 | 27 | 22 | 10 | 11 | 13 | 15 | - | C | 1 | 0 | 0 | 0 | 0 | 0 | 0 | 0 | 0 | 0 | 0 | 0 | 0 | 0 | 0 | 0 | 0 | 1 |
| H202 | 12 | 13 | 30 | 21 | 12 | 12 | 14 | 16 | + | C | 1 | 0 | 0 | 0 | 0 | 0 | 0 | 0 | 0 | 0 | 0 | 0 | 0 | 0 | 0 | 0 | 0 | 1 |
| H203 | 11 | 12 | 29 | 21 | 11 | 12 | 14 | 15 | + | C | 1 | 0 | 0 | 0 | 0 | 0 | 0 | 0 | 0 | 0 | 0 | 0 | 0 | 0 | 0 | 0 | 0 | 1 |
| H204 | 11 | 13 | 31 | 21 | 10 | 10 | 12 | 15 | + | C | 1 | 0 | 0 | 0 | 0 | 0 | 0 | 0 | 0 | 0 | 0 | 0 | 0 | 0 | 0 | 0 | 0 | 1 |
| H205 | 11 | 14 | 32 | 21 | 11 | 10 | 13 | 15 | + | C | 1 | 0 | 0 | 0 | 0 | 0 | 0 | 0 | 0 | 0 | 0 | 0 | 0 | 0 | 0 | 0 | 0 | 1 |
| H206 | 11 | 13 | 29 | 24 | 12 | 14 | 12 | 14 | - | T | 1 | 0 | 0 | 0 | 0 | 0 | 0 | 0 | 0 | 0 | 0 | 0 | 0 | 0 | 0 | 0 | 0 | 1 |
| H207 | 12 | 14 | 32 | 23 | 12 | 13 | 14 | 15 | - | T | 1 | 0 | 0 | 0 | 0 | 0 | 0 | 0 | 0 | 0 | 0 | 0 | 0 | 0 | 0 | 0 | 0 | 1 |
| H208 | 11 | 13 | 30 | 21 | 12 | 12 | 12 | 15 | + | C | 1 | 0 | 0 | 0 | 0 | 0 | 0 | 0 | 0 | 0 | 0 | 0 | 0 | 0 | 0 | 0 | 0 | 1 |
| H209 | 11 | 11 | 28 | 21 | 11 | 10 | 12 | 15 | + | C | 1 | 0 | 0 | 0 | 0 | 0 | 0 | 0 | 0 | 0 | 0 | 0 | 0 | 0 | 0 | 0 | 0 | 1 |
| H210 | 11 | 12 | 29 | 22 | 10 | 11 | 12 | 17 | + | C | 1 | 0 | 0 | 0 | 0 | 0 | 0 | 0 | 0 | 0 | 0 | 0 | 0 | 0 | 0 | 0 | 0 | 1 |
| H211 | 11 | 13 | 30 | 20 | 11 | 11 | 13 | 15 | + | C | 1 | 0 | 0 | 0 | 0 | 0 | 0 | 0 | 0 | 0 | 0 | 0 | 0 | 0 | 0 | 0 | 0 | 1 |
| H212 | 11 | 13 | 30 | 23 | 10 | 10 | 12 | 13 | - | T | 1 | 0 | 0 | 0 | 0 | 0 | 0 | 0 | 0 | 0 | 0 | 0 | 0 | 0 | 0 | 0 | 0 | 1 |
| H213 | 12 | 12 | 29 | 21 | 11 | 10 | 14 | 16 | + | C | 1 | 0 | 0 | 0 | 0 | 0 | 0 | 0 | 0 | 0 | 0 | 0 | 0 | 0 | 0 | 0 | 0 | 1 |
| H214 | 11 | 13 | 31 | 21 | 11 | 11 | 12 | 15 | + | C | 1 | 0 | 0 | 0 | 0 | 0 | 0 | 0 | 0 | 0 | 0 | 0 | 0 | 0 | 0 | 0 | 0 | 1 |
| H215 | 11 | 13 | 32 | 21 | 11 | 12 | 12 | 15 | + | C | 1 | 0 | 0 | 0 | 0 | 0 | 0 | 0 | 0 | 0 | 0 | 0 | 0 | 0 | 0 | 0 | 0 | 1 |
| H216 | 12 | 14 | 32 | 21 | 10 | 10 | 13 | 15 | + | C | 1 | 0 | 0 | 0 | 0 | 0 | 0 | 0 | 0 | 1 | 0 | 0 | 0 | 2 | 0 | 0 | 0 | 4 |
| H217 | 12 | 12 | 28 | 25 | 12 | 15 | 12 | 14 | - | T | 1 | 0 | 0 | 0 | 0 | 0 | 0 | 0 | 0 | 0 | 0 | 0 | 0 | 0 | 0 | 0 | 0 | 1 |
| H218 | 13 | 13 | 30 | 23 | 11 | 11 | 14 | 15 | - | T | 1 | 0 | 0 | 0 | 0 | 0 | 0 | 0 | 0 | 0 | 0 | 0 | 0 | 0 | 0 | 0 | 0 | 1 |
| H219 | 12 | 13 | 30 | 21 | 11 | 12 | 14 | 14 | + | C | 1 | 0 | 0 | 0 | 0 | 0 | 0 | 0 | 0 | 0 | 0 | 0 | 0 | 0 | 0 | 0 | 0 | 1 |
| H220 | 12 | 13 | 30 | 21 | 12 | 12 | 13 | 17 | + | C | 1 | 0 | 0 | 0 | 0 | 0 | 0 | 0 | 0 | 0 | 0 | 0 | 0 | 0 | 0 | 0 | 0 | 1 |
| H221 | 12 | 13 | 30 | 21 | 11 | 13 | 13 | 16 | + | C | 1 | 0 | 0 | 0 | 0 | 0 | 0 | 0 | 0 | 0 | 0 | 0 | 0 | 0 | 0 | 0 | 1 | 2 |
| H222 | 12 | 13 | 31 | 21 | 10 | 10 | 12 | 15 | - | C | 1 | 0 | 0 | 0 | 0 | 0 | 0 | 0 | 0 | 0 | 0 | 0 | 0 | 0 | 0 | 0 | 0 | 1 |
| H223 | 12 | 13 | 31 | 24 | 11 | 10 | 12 | 13 | + | C | 1 | 0 | 0 | 0 | 0 | 0 | 0 | 0 | 0 | 0 | 0 | 0 | 0 | 0 | 0 | 0 | 0 | 1 |
| H224 | 12 | 14 | 30 | 24 | 12 | 14 | 12 | 14 | - | T | 1 | 0 | 0 | 0 | 0 | 0 | 0 | 0 | 0 | 0 | 0 | 0 | 0 | 0 | 0 | 0 | 0 | 1 |
| H225 | 13 | 14 | 30 | 24 | 12 | 14 | 12 | 14 | - | T | 1 | 0 | 0 | 0 | 0 | 0 | 0 | 0 | 0 | 0 | 0 | 0 | 0 | 0 | 0 | 0 | 0 | 1 |
| H226 | 12 | 13 | 30 | 25 | 12 | 12 | 13 | 17 | - | T | 1 | 0 | 0 | 0 | 0 | 0 | 0 | 0 | 0 | 0 | 0 | 0 | 0 | 0 | 0 | 0 | 0 | 1 |
| H227 | 12 | 14 | 31 | 21 | 12 | 12 | 14 | 17 | + | C | 1 | 0 | 0 | 2 | 0 | 0 | 0 | 0 | 0 | 0 | 0 | 0 | 0 | 0 | 0 | 0 | 0 | 3 |
| H228 | 12 | 13 | 29 | 23 | 12 | 14 | 12 | 14 | - | T | 1 | 0 | 0 | 0 | 1 | 0 | 0 | 0 | 0 | 0 | 0 | 0 | 0 | 0 | 0 | 0 | 0 | 2 |
| H229 | 12 | 13 | 28 | 24 | 12 | 13 | 12 | 14 | - | T | 1 | 0 | 0 | 0 | 0 | 0 | 0 | 0 | 0 | 0 | 0 | 0 | 0 | 0 | 0 | 0 | 0 | 1 |
| H230 | 12 | 13 | 31 | 21 | 12 | 12 | 15 | 15 | + | C | 1 | 0 | 0 | 0 | 0 | 0 | 0 | 0 | 0 | 0 | 0 | 0 | 0 | 0 | 0 | 0 | 0 | 1 |
| H231 | 12 | 12 | 29 | 21 | 11 | 12 | 12 | 15 | + | C | 1 | 0 | 0 | 0 | 0 | 0 | 0 | 0 | 0 | 0 | 0 | 0 | 0 | 0 | 0 | 0 | 0 | 1 |
| H232 | 12 | 12 | 28 | 25 | 12 | 12 | 13 | 14 | + | C | 1 | 0 | 0 | 0 | 0 | 0 | 0 | 0 | 0 | 0 | 0 | 0 | 0 | 0 | 0 | 0 | 0 | 1 |

| | | | | | | | | | | | | | | | | | | | | | | | | | | | | |
|---|---|---|---|---|---|---|---|---|---|---|---|---|---|---|---|---|---|---|---|---|---|---|---|---|---|---|---|---|
| H233 | 12 | 13 | 27 | 21 | 10 | 12 | 13 | 17 | + | C | 1 | 0 | 0 | 0 | 0 | 0 | 0 | 0 | 0 | 0 | 0 | 0 | 0 | 0 | 0 | 0 | 0 | 1 |
| H234 | 12 | 14 | 31 | 21 | 11 | 13 | 12 | 16 | + | C | 1 | 0 | 0 | 0 | 0 | 0 | 0 | 0 | 0 | 0 | 0 | 0 | 0 | 0 | 0 | 0 | 0 | 1 |
| H235 | 12 | 14 | 30 | 21 | 11 | 12 | 13 | 16 | + | C | 1 | 0 | 0 | 0 | 0 | 0 | 0 | 0 | 0 | 0 | 0 | 0 | 0 | 0 | 0 | 0 | 0 | 1 |
| H236 | 12 | 12 | 31 | 21 | 11 | 12 | 12 | 16 | + | C | 1 | 0 | 0 | 0 | 0 | 0 | 0 | 0 | 0 | 0 | 0 | 0 | 0 | 0 | 0 | 0 | 0 | 1 |
| H237 | 12 | 13 | 29 | 24 | 12 | 14 | 12 | 14 | - | T | 2 | 0 | 0 | 0 | 1 | 0 | 0 | 0 | 0 | 0 | 0 | 0 | 0 | 0 | 0 | 0 | 0 | 3 |
| H238 | 12 | 13 | 30 | 21 | 12 | 12 | 12 | 15 | + | C | 1 | 0 | 0 | 0 | 0 | 0 | 0 | 0 | 0 | 0 | 0 | 0 | 0 | 0 | 0 | 0 | 0 | 1 |
| H239 | 10 | 14 | 31 | 25 | 11 | 12 | 13 | 16 | - | C | 1 | 0 | 0 | 0 | 0 | 0 | 0 | 0 | 0 | 0 | 0 | 0 | 0 | 0 | 0 | 0 | 0 | 1 |
| H240 | 10 | 13 | 32 | 23 | 12 | 11 | 12 | 15 | - | C | 1 | 0 | 0 | 0 | 0 | 0 | 0 | 0 | 0 | 0 | 0 | 0 | 0 | 0 | 0 | 0 | 0 | 1 |
| H241 | 12 | 14 | 30 | 21 | 11 | 12 | 13 | 17 | + | C | 1 | 0 | 0 | 1 | 0 | 0 | 0 | 0 | 0 | 0 | 0 | 0 | 0 | 0 | 0 | 0 | 0 | 2 |
| H242 | 12 | 13 | 31 | 21 | 12 | 12 | 13 | 15 | + | C | 2 | 0 | 0 | 1 | 0 | 0 | 0 | 1 | 0 | 0 | 0 | 0 | 0 | 0 | 0 | 0 | 0 | 4 |
| H243 | 12 | 13 | 30 | 24 | 11 | 14 | 11 | 14 | - | T | 1 | 0 | 0 | 0 | 0 | 0 | 0 | 0 | 0 | 0 | 0 | 0 | 0 | 0 | 0 | 0 | 0 | 1 |
| H244 | 12 | 13 | 30 | 21 | 11 | 12 | 12 | 17 | + | C | 1 | 0 | 0 | 0 | 0 | 6 | 0 | 0 | 0 | 0 | 0 | 0 | 0 | 0 | 0 | 0 | 0 | 7 |
| H245 | 12 | 13 | 29 | 24 | 11 | 14 | 13 | 14 | - | T | 1 | 0 | 0 | 0 | 0 | 0 | 0 | 0 | 0 | 0 | 0 | 0 | 0 | 0 | 0 | 0 | 0 | 1 |
| H246 | 12 | 14 | 31 | 21 | 11 | 12 | 14 | 16 | + | C | 3 | 0 | 0 | 1 | 0 | 0 | 0 | 1 | 1 | 0 | 0 | 0 | 0 | 0 | 0 | 0 | 0 | 6 |
| H247 | 12 | 14 | 32 | 21 | 11 | 12 | 13 | 16 | + | C | 1 | 0 | 0 | 0 | 0 | 0 | 0 | 0 | 0 | 0 | 0 | 0 | 0 | 0 | 1 | 0 | 0 | 2 |
| H248 | 10 | 12 | 29 | 22 | 11 | 12 | 13 | 14 | - | C | 1 | 0 | 0 | 0 | 0 | 0 | 0 | 0 | 0 | 0 | 0 | 0 | 0 | 0 | 0 | 0 | 0 | 1 |
| H249 | 14 | 14 | 29 | 22 | 11 | 12 | 13 | 14 | - | T | 1 | 0 | 0 | 0 | 0 | 0 | 0 | 0 | 0 | 0 | 0 | 0 | 0 | 0 | 0 | 0 | 0 | 1 |
| H250 | 13 | 14 | 31 | 22 | 11 | 13 | 13 | 17 | + | C | 1 | 0 | 0 | 0 | 0 | 0 | 0 | 0 | 0 | 0 | 0 | 0 | 0 | 0 | 0 | 0 | 0 | 1 |
| H251 | 12 | 15 | 32 | 21 | 11 | 12 | 12 | 15 | + | C | 1 | 0 | 0 | 0 | 0 | 0 | 0 | 0 | 0 | 0 | 0 | 0 | 0 | 0 | 0 | 0 | 0 | 1 |
| H252 | 12 | 12 | 28 | 25 | 12 | 14 | 13 | 15 | - | T | 1 | 0 | 0 | 0 | 0 | 0 | 0 | 0 | 0 | 0 | 0 | 0 | 0 | 0 | 0 | 0 | 0 | 1 |
| H253 | 12 | 14 | 31 | 20 | 11 | 12 | 14 | 14 | - | C | 1 | 0 | 0 | 0 | 0 | 0 | 0 | 0 | 0 | 0 | 0 | 0 | 0 | 0 | 0 | 0 | 0 | 1 |
| H254 | 10 | 12 | 29 | 22 | 12 | 12 | 13 | 14 | - | C | 1 | 0 | 0 | 0 | 0 | 0 | 0 | 0 | 0 | 0 | 0 | 0 | 0 | 0 | 0 | 0 | 0 | 1 |
| H255 | 13 | 13 | 29 | 21 | 11 | 12 | 13 | 16 | + | C | 1 | 0 | 0 | 0 | 0 | 0 | 0 | 0 | 0 | 0 | 0 | 0 | 0 | 0 | 0 | 0 | 0 | 1 |
| H256 | 12 | 14 | 31 | 20 | 12 | 12 | 12 | 15 | - | C | 1 | 0 | 0 | 0 | 0 | 0 | 0 | 0 | 0 | 0 | 0 | 0 | 0 | 0 | 0 | 0 | 0 | 1 |
| H257 | 12 | 14 | 32 | 21 | 11 | 13 | 13 | 15 | + | C | 1 | 0 | 0 | 0 | 0 | 0 | 0 | 0 | 0 | 0 | 0 | 0 | 0 | 0 | 0 | 0 | 0 | 1 |
| H258 | 14 | 12 | 28 | 22 | 11 | 12 | 11 | 14 | - | T | 1 | 0 | 0 | 0 | 0 | 0 | 0 | 0 | 0 | 0 | 0 | 0 | 0 | 0 | 0 | 0 | 0 | 1 |
| H259 | 10 | 14 | 32 | 24 | 12 | 12 | 13 | 15 | - | C | 1 | 0 | 0 | 0 | 0 | 0 | 0 | 0 | 0 | 0 | 0 | 0 | 0 | 0 | 0 | 0 | 0 | 1 |
| H260 | 12 | 14 | 30 | 24 | 11 | 14 | 12 | 14 | - | T | 1 | 0 | 0 | 0 | 0 | 0 | 0 | 0 | 0 | 0 | 0 | 0 | 0 | 0 | 0 | 0 | 0 | 1 |
| H261 | 12 | 13 | 27 | 21 | 11 | 12 | 14 | 16 | + | C | 1 | 0 | 0 | 0 | 0 | 0 | 0 | 0 | 0 | 0 | 0 | 0 | 0 | 0 | 0 | 0 | 0 | 1 |
| H262 | 12 | 13 | 30 | 25 | 12 | 14 | 12 | 14 | - | T | 1 | 0 | 0 | 0 | 0 | 0 | 0 | 0 | 0 | 0 | 0 | 0 | 0 | 0 | 0 | 0 | 0 | 1 |
| H263 | 12 | 14 | 30 | 21 | 12 | 12 | 14 | 17 | + | C | 1 | 0 | 0 | 1 | 0 | 0 | 0 | 0 | 0 | 0 | 0 | 0 | 0 | 0 | 0 | 0 | 0 | 2 |
| H264 | 12 | 14 | 31 | 21 | 12 | 12 | 14 | 15 | + | C | 1 | 0 | 0 | 0 | 0 | 0 | 0 | 0 | 0 | 0 | 0 | 0 | 0 | 0 | 0 | 0 | 0 | 1 |
| H265 | 13 | 13 | 31 | 22 | 12 | 12 | 13 | 17 | + | C | 1 | 0 | 0 | 0 | 0 | 0 | 0 | 0 | 0 | 0 | 0 | 0 | 0 | 0 | 0 | 0 | 0 | 1 |
| H266 | 12 | 13 | 32 | 21 | 11 | 12 | 13 | 17 | + | C | 1 | 0 | 0 | 0 | 0 | 0 | 0 | 0 | 0 | 0 | 0 | 0 | 0 | 0 | 0 | 0 | 0 | 1 |
| H267 | 12 | 14 | 31 | 21 | 12 | 12 | 14 | 16 | + | C | 1 | 0 | 0 | 0 | 0 | 0 | 0 | 0 | 0 | 0 | 0 | 0 | 0 | 0 | 0 | 0 | 0 | 1 |
| H268 | 12 | 13 | 31 | 21 | 11 | 11 | 12 | 15 | + | C | 1 | 1 | 0 | 0 | 0 | 3 | 0 | 0 | 0 | 0 | 0 | 0 | 0 | 0 | 0 | 0 | 0 | 5 |
| H269 | 12 | 14 | 32 | 21 | 11 | 12 | 12 | 16 | + | C | 1 | 0 | 0 | 0 | 0 | 0 | 0 | 0 | 0 | 0 | 0 | 0 | 0 | 0 | 0 | 0 | 1 | 2 |
| H270 | 12 | 13 | 30 | 21 | 11 | 12 | 15 | 17 | + | C | 1 | 0 | 0 | 1 | 0 | 0 | 0 | 0 | 1 | 0 | 0 | 0 | 0 | 0 | 0 | 0 | 0 | 3 |
| H271 | 12 | 13 | 29 | 24 | 11 | 14 | 13 | 13 | - | T | 1 | 0 | 0 | 0 | 0 | 0 | 0 | 0 | 0 | 0 | 0 | 0 | 0 | 0 | 0 | 0 | 0 | 1 |
| H272 | 12 | 13 | 31 | 22 | 10 | 12 | 13 | 13 | + | C | 1 | 0 | 0 | 0 | 0 | 0 | 0 | 0 | 0 | 0 | 0 | 0 | 0 | 0 | 0 | 0 | 0 | 1 |
| H273 | 12 | 13 | 31 | 22 | 10 | 12 | 13 | 16 | + | C | 1 | 0 | 0 | 0 | 0 | 0 | 0 | 0 | 0 | 0 | 0 | 0 | 0 | 0 | 0 | 0 | 0 | 1 |
| H274 | 12 | 14 | 29 | 25 | 12 | 13 | 13 | 14 | - | T | 1 | 0 | 0 | 0 | 0 | 0 | 0 | 0 | 0 | 0 | 0 | 0 | 0 | 0 | 0 | 0 | 0 | 1 |
| H275 | 12 | 12 | 30 | 22 | 10 | 12 | 12 | 13 | + | C | 1 | 0 | 0 | 0 | 0 | 0 | 0 | 0 | 0 | 0 | 0 | 0 | 0 | 0 | 0 | 0 | 0 | 1 |
| H276 | 12 | 14 | 32 | 21 | 10 | 11 | 12 | 15 | + | C | 1 | 1 | 0 | 0 | 0 | 0 | 0 | 0 | 0 | 0 | 0 | 0 | 0 | 0 | 0 | 0 | 0 | 2 |
| H277 | 12 | 14 | 30 | 25 | 11 | 14 | 13 | 14 | - | T | 1 | 0 | 0 | 0 | 0 | 0 | 0 | 0 | 0 | 0 | 0 | 0 | 0 | 0 | 0 | 0 | 0 | 1 |
| H278 | 14 | 12 | 28 | 21 | 11 | 12 | 11 | 14 | - | T | 1 | 0 | 0 | 0 | 0 | 0 | 0 | 0 | 0 | 0 | 0 | 0 | 0 | 0 | 0 | 0 | 0 | 1 |
| H279 | 10 | 13 | 31 | 26 | 11 | 11 | 12 | 15 | - | T | 1 | 0 | 0 | 0 | 0 | 0 | 0 | 0 | 0 | 0 | 0 | 0 | 0 | 0 | 0 | 0 | 0 | 1 |
| H280 | 12 | 13 | 31 | 21 | 11 | 11 | 14 | 17 | + | C | 1 | 0 | 0 | 0 | 0 | 2 | 0 | 0 | 0 | 0 | 0 | 0 | 0 | 0 | 0 | 0 | 0 | 3 |
| H281 | 12 | 13 | 32 | 21 | 11 | 11 | 12 | 15 | + | C | 1 | 0 | 0 | 0 | 0 | 0 | 0 | 0 | 0 | 0 | 0 | 0 | 0 | 0 | 0 | 0 | 0 | 1 |
| H282 | 12 | 13 | 29 | 25 | 11 | 12 | 12 | 16 | - | T | 1 | 0 | 0 | 0 | 0 | 0 | 0 | 0 | 0 | 0 | 0 | 0 | 0 | 0 | 0 | 0 | 0 | 1 |
| H283 | 12 | 13 | 31 | 22 | 11 | 12 | 13 | 15 | + | C | 1 | 0 | 0 | 0 | 0 | 0 | 0 | 0 | 0 | 0 | 0 | 0 | 0 | 0 | 0 | 0 | 0 | 1 |
| H284 | 12 | 12 | 29 | 21 | 11 | 12 | 13 | 15 | + | C | 1 | 0 | 0 | 0 | 0 | 0 | 0 | 1 | 0 | 0 | 0 | 0 | 0 | 0 | 0 | 0 | 0 | 2 |
| H285 | 13 | 13 | 30 | 21 | 11 | 12 | 13 | 16 | + | C | 1 | 0 | 0 | 0 | 0 | 0 | 0 | 0 | 0 | 0 | 0 | 0 | 0 | 0 | 0 | 0 | 0 | 1 |
| H286 | 12 | 13 | 30 | 21 | 11 | 11 | 12 | 15 | + | C | 1 | 0 | 0 | 0 | 0 | 0 | 0 | 0 | 0 | 0 | 0 | 0 | 0 | 0 | 0 | 0 | 0 | 1 |
| H287 | 12 | 14 | 30 | 24 | 12 | 13 | 12 | 14 | - | T | 1 | 0 | 0 | 0 | 0 | 0 | 0 | 0 | 0 | 0 | 0 | 0 | 0 | 0 | 0 | 0 | 0 | 1 |
| H288 | 12 | 13 | 30 | 21 | 11 | 12 | 11 | 15 | + | C | 1 | 0 | 0 | 0 | 0 | 0 | 0 | 0 | 0 | 0 | 0 | 0 | 0 | 0 | 0 | 0 | 0 | 1 |
| H289 | 12 | 14 | 30 | 23 | 11 | 13 | 12 | 14 | - | T | 1 | 0 | 0 | 0 | 1 | 0 | 0 | 0 | 0 | 0 | 0 | 0 | 0 | 0 | 0 | 0 | 0 | 2 |
| H290 | 12 | 13 | 30 | 21 | 11 | 11 | 13 | 16 | + | C | 1 | 0 | 0 | 0 | 0 | 0 | 0 | 0 | 0 | 0 | 0 | 0 | 0 | 0 | 0 | 0 | 0 | 1 |
| H291 | 10 | 13 | 32 | 24 | 11 | 12 | 12 | 15 | - | T | 1 | 0 | 0 | 0 | 0 | 0 | 0 | 0 | 0 | 0 | 0 | 0 | 0 | 0 | 0 | 0 | 0 | 1 |

| | | | | | | | | | | | | | | | | | | | | | | | | | | | | |
|---|---|---|---|---|---|---|---|---|---|---|---|---|---|---|---|---|---|---|---|---|---|---|---|---|---|---|---|---|
| H292 | 15 | 13 | 31 | 23 | 11 | 12 | 11 | 14 | - | T | 1 | 0 | 0 | 0 | 0 | 0 | 0 | 0 | 0 | 0 | 0 | 0 | 0 | 0 | 0 | 0 | 0 | 1 |
| H293 | 12 | 13 | 32 | 21 | 10 | 12 | 13 | 14 | + | C | 1 | 0 | 0 | 0 | 0 | 0 | 0 | 0 | 0 | 0 | 0 | 0 | 0 | 0 | 0 | 0 | 0 | 1 |
| H294 | 12 | 14 | 29 | 24 | 12 | 13 | 12 | 14 | - | T | 1 | 0 | 0 | 0 | 0 | 0 | 0 | 0 | 0 | 0 | 0 | 0 | 0 | 0 | 0 | 0 | 0 | 1 |
| H295 | 12 | 12 | 28 | 25 | 11 | 12 | 12 | 14 | + | C | 1 | 0 | 0 | 0 | 0 | 0 | 0 | 0 | 0 | 0 | 0 | 0 | 0 | 0 | 0 | 0 | 0 | 1 |
| H296 | 13 | 13 | 29 | 22 | 11 | 11 | 13 | 16 | - | C | 1 | 0 | 0 | 0 | 0 | 0 | 0 | 0 | 0 | 0 | 0 | 0 | 0 | 0 | 0 | 0 | 0 | 1 |
| H297 | 15 | 14 | 30 | 21 | 11 | 12 | 12 | 16 | - | T | 1 | 0 | 0 | 0 | 0 | 0 | 0 | 0 | 0 | 0 | 0 | 0 | 0 | 0 | 0 | 0 | 0 | 1 |
| H298 | 12 | 13 | 29 | 21 | 11 | 13 | 14 | 15 | - | T | 1 | 0 | 0 | 0 | 0 | 0 | 0 | 0 | 0 | 0 | 0 | 0 | 0 | 0 | 0 | 0 | 0 | 1 |
| H299 | 12 | 15 | 32 | 21 | 11 | 12 | 13 | 16 | + | C | 1 | 0 | 0 | 0 | 0 | 0 | 0 | 0 | 0 | 0 | 0 | 0 | 1 | 0 | 0 | 0 | 0 | 2 |
| H300 | 12 | 13 | 29 | 22 | 12 | 14 | 12 | 14 | - | T | 1 | 0 | 0 | 0 | 0 | 0 | 0 | 0 | 0 | 0 | 0 | 0 | 0 | 0 | 0 | 0 | 0 | 1 |
| H301 | 12 | 13 | 32 | 21 | 11 | 12 | 12 | 15 | + | C | 1 | 0 | 0 | 0 | 0 | 0 | 0 | 0 | 0 | 0 | 2 | 0 | 0 | 0 | 0 | 1 | 1 | 5 |
| H302 | 10 | 13 | 32 | 24 | 12 | 12 | 13 | 15 | - | C | 1 | 0 | 0 | 0 | 0 | 0 | 0 | 0 | 0 | 0 | 0 | 0 | 0 | 0 | 0 | 0 | 0 | 1 |
| H303 | 12 | 13 | 30 | 21 | 12 | 12 | 13 | 16 | + | C | 1 | 0 | 0 | 0 | 0 | 0 | 0 | 0 | 0 | 0 | 0 | 0 | 0 | 0 | 0 | 0 | 1 | 2 |
| H304 | 13 | 15 | 32 | 22 | 11 | 12 | 13 | 16 | + | C | 1 | 0 | 0 | 0 | 0 | 0 | 0 | 0 | 0 | 0 | 0 | 0 | 0 | 0 | 0 | 0 | 0 | 1 |
| H305 | 12 | 14 | 32 | 21 | 12 | 12 | 14 | 15 | + | C | 1 | 0 | 0 | 0 | 0 | 0 | 0 | 0 | 0 | 0 | 0 | 0 | 0 | 0 | 0 | 0 | 0 | 1 |
| H306 | 12 | 14 | 32 | 21 | 11 | 12 | 13 | 15 | + | C | 5 | 0 | 0 | 1 | 0 | 1 | 1 | 0 | 0 | 0 | 0 | 0 | 2 | 0 | 0 | 4 | 2 | 16 |
| H307 | 12 | 13 | 29 | 23 | 12 | 13 | 13 | 14 | - | T | 1 | 0 | 0 | 0 | 0 | 0 | 0 | 0 | 0 | 0 | 0 | 0 | 0 | 0 | 0 | 0 | 0 | 1 |
| H308 | 13 | 12 | 29 | 22 | 11 | 12 | 13 | 15 | + | C | 1 | 0 | 0 | 0 | 0 | 0 | 0 | 0 | 0 | 0 | 0 | 0 | 0 | 0 | 0 | 0 | 0 | 1 |
| H309 | 12 | 15 | 32 | 21 | 11 | 11 | 13 | 15 | + | C | 2 | 0 | 0 | 0 | 0 | 0 | 0 | 0 | 0 | 0 | 0 | 0 | 0 | 0 | 0 | 0 | 0 | 2 |
| H310 | 12 | 12 | 29 | 24 | 12 | 14 | 12 | 14 | - | T | 1 | 0 | 0 | 0 | 0 | 0 | 0 | 0 | 0 | 0 | 0 | 0 | 0 | 0 | 0 | 0 | 0 | 1 |
| H311 | 12 | 14 | 30 | 22 | 11 | 12 | 12 | 15 | + | C | 1 | 0 | 0 | 0 | 0 | 0 | 0 | 0 | 0 | 0 | 0 | 0 | 0 | 0 | 0 | 0 | 0 | 1 |
| H312 | 12 | 14 | 31 | 24 | 11 | 12 | 11 | 13 | + | C | 1 | 0 | 0 | 0 | 0 | 0 | 0 | 0 | 0 | 0 | 0 | 0 | 0 | 0 | 0 | 0 | 0 | 1 |
| H313 | 14 | 13 | 30 | 22 | 11 | 12 | 12 | 14 | - | T | 1 | 0 | 0 | 0 | 0 | 0 | 0 | 0 | 0 | 0 | 0 | 0 | 0 | 0 | 0 | 0 | 0 | 1 |
| H314 | 12 | 13 | 30 | 21 | 12 | 12 | 13 | 15 | + | C | 1 | 0 | 0 | 0 | 0 | 1 | 0 | 0 | 0 | 0 | 0 | 0 | 0 | 0 | 0 | 0 | 0 | 2 |
| H315 | 12 | 13 | 29 | 21 | 11 | 12 | 14 | 17 | + | C | 1 | 0 | 0 | 0 | 0 | 0 | 0 | 0 | 0 | 0 | 0 | 0 | 0 | 0 | 0 | 0 | 0 | 1 |
| H316 | 12 | 14 | 30 | 21 | 11 | 13 | 15 | 16 | + | C | 1 | 0 | 0 | 0 | 0 | 0 | 0 | 0 | 0 | 0 | 0 | 0 | 0 | 0 | 0 | 0 | 0 | 1 |
| H317 | 12 | 14 | 31 | 21 | 11 | 12 | 12 | 16 | + | C | 1 | 0 | 0 | 0 | 0 | 0 | 0 | 1 | 0 | 0 | 0 | 0 | 0 | 0 | 0 | 0 | 0 | 2 |
| H318 | 12 | 14 | 31 | 25 | 11 | 12 | 13 | 14 | - | T | 1 | 0 | 0 | 0 | 0 | 0 | 0 | 0 | 0 | 0 | 0 | 0 | 0 | 0 | 0 | 0 | 0 | 1 |
| H319 | 12 | 14 | 33 | 21 | 11 | 13 | 12 | 16 | + | C | 1 | 0 | 0 | 0 | 0 | 0 | 0 | 0 | 0 | 0 | 0 | 0 | 0 | 0 | 0 | 0 | 0 | 1 |
| H320 | 12 | 14 | 32 | 21 | 12 | 12 | 13 | 15 | + | C | 1 | 0 | 0 | 0 | 0 | 0 | 0 | 0 | 0 | 0 | 0 | 0 | 0 | 0 | 0 | 0 | 0 | 1 |
| H321 | 13 | 14 | 30 | 21 | 11 | 12 | 15 | 17 | + | C | 1 | 0 | 0 | 0 | 0 | 0 | 0 | 0 | 0 | 0 | 0 | 0 | 0 | 0 | 0 | 0 | 0 | 1 |
| H322 | 12 | 12 | 28 | 24 | 11 | 14 | 12 | 15 | - | T | 1 | 0 | 0 | 0 | 0 | 0 | 0 | 0 | 0 | 0 | 0 | 0 | 0 | 0 | 0 | 0 | 0 | 1 |
| H323 | 12 | 14 | 31 | 22 | 11 | 12 | 15 | 17 | + | C | 1 | 0 | 0 | 0 | 0 | 0 | 0 | 0 | 0 | 0 | 0 | 0 | 0 | 0 | 0 | 0 | 0 | 1 |
| H324 | 12 | 14 | 29 | 25 | 12 | 14 | 13 | 14 | - | T | 1 | 0 | 0 | 0 | 0 | 0 | 0 | 0 | 0 | 0 | 0 | 0 | 0 | 0 | 0 | 0 | 0 | 1 |
| H325 | 12 | 13 | 30 | 21 | 12 | 12 | 14 | 17 | + | C | 1 | 0 | 0 | 0 | 0 | 0 | 0 | 0 | 0 | 0 | 0 | 0 | 0 | 0 | 0 | 0 | 0 | 1 |
| H326 | 12 | 15 | 31 | 25 | 12 | 14 | 13 | 14 | - | T | 1 | 0 | 0 | 0 | 0 | 0 | 0 | 0 | 0 | 0 | 0 | 0 | 0 | 0 | 0 | 0 | 0 | 1 |
| H327 | 12 | 15 | 32 | 22 | 11 | 12 | 14 | 17 | + | C | 1 | 0 | 0 | 0 | 0 | 0 | 0 | 0 | 0 | 0 | 0 | 0 | 0 | 0 | 0 | 0 | 0 | 1 |
| H328 | 12 | 13 | 29 | 25 | 11 | 12 | 12 | 14 | + | C | 1 | 0 | 0 | 0 | 0 | 0 | 0 | 0 | 0 | 0 | 0 | 0 | 0 | 0 | 0 | 0 | 0 | 1 |
| H329 | 12 | 14 | 30 | 21 | 11 | 12 | 15 | 17 | + | C | 1 | 0 | 0 | 0 | 0 | 1 | 0 | 0 | 0 | 0 | 0 | 0 | 0 | 0 | 0 | 0 | 0 | 2 |
| H330 | 12 | 13 | 30 | 21 | 11 | 13 | 15 | 14 | + | C | 1 | 0 | 0 | 0 | 0 | 0 | 0 | 0 | 0 | 0 | 0 | 0 | 0 | 0 | 0 | 0 | 0 | 1 |
| H331 | 12 | 14 | 32 | 21 | 12 | 13 | 12 | 16 | + | C | 1 | 0 | 0 | 0 | 0 | 0 | 0 | 0 | 0 | 0 | 0 | 0 | 0 | 0 | 0 | 0 | 0 | 1 |
| H332 | 12 | 13 | 30 | 22 | 12 | 14 | 12 | 14 | - | T | 1 | 0 | 0 | 0 | 0 | 0 | 0 | 0 | 0 | 0 | 0 | 0 | 0 | 0 | 0 | 0 | 0 | 1 |
| H333 | 12 | 12 | 30 | 21 | 11 | 12 | 13 | 16 | + | C | 1 | 0 | 0 | 0 | 0 | 0 | 0 | 1 | 0 | 0 | 0 | 0 | 0 | 0 | 0 | 0 | 0 | 2 |
| H334 | 12 | 13 | 29 | 27 | 12 | 12 | 13 | 15 | - | T | 1 | 0 | 0 | 0 | 0 | 0 | 0 | 0 | 0 | 0 | 0 | 0 | 0 | 0 | 0 | 0 | 0 | 1 |
| H335 | 12 | 14 | 30 | 21 | 12 | 12 | 13 | 15 | - | C | 1 | 0 | 0 | 0 | 0 | 0 | 0 | 0 | 0 | 0 | 0 | 0 | 0 | 0 | 0 | 0 | 0 | 1 |
| H336 | 12 | 13 | 29 | 25 | 11 | 12 | 14 | 15 | - | T | 1 | 0 | 0 | 0 | 0 | 0 | 0 | 0 | 0 | 0 | 0 | 0 | 0 | 0 | 0 | 0 | 0 | 1 |
| H337 | 12 | 14 | 30 | 21 | 11 | 13 | 14 | 15 | + | C | 1 | 0 | 0 | 0 | 0 | 0 | 0 | 0 | 0 | 0 | 0 | 0 | 0 | 0 | 0 | 0 | 0 | 1 |
| H338 | 12 | 13 | 29 | 23 | 12 | 12 | 13 | 15 | - | T | 1 | 0 | 0 | 0 | 0 | 0 | 0 | 0 | 0 | 0 | 0 | 0 | 0 | 0 | 0 | 0 | 0 | 1 |
| H339 | 12 | 14 | 30 | 23 | 11 | 12 | 12 | 15 | - | C | 1 | 0 | 0 | 0 | 0 | 0 | 0 | 0 | 0 | 0 | 0 | 0 | 0 | 0 | 0 | 0 | 0 | 1 |
| H340 | 12 | 14 | 32 | 20 | 12 | 13 | 14 | 15 | + | C | 1 | 0 | 0 | 0 | 0 | 0 | 0 | 0 | 0 | 0 | 0 | 0 | 0 | 0 | 0 | 0 | 0 | 1 |
| H341 | 12 | 14 | 31 | 21 | 11 | 12 | 15 | 15 | + | C | 1 | 0 | 0 | 0 | 0 | 0 | 0 | 0 | 0 | 0 | 0 | 0 | 1 | 0 | 0 | 0 | 0 | 2 |
| H342 | 12 | 12 | 29 | 22 | 11 | 12 | 13 | 15 | + | C | 1 | 0 | 0 | 0 | 0 | 0 | 0 | 0 | 0 | 0 | 0 | 0 | 0 | 0 | 0 | 0 | 0 | 1 |
| H343 | 12 | 14 | 30 | 22 | 12 | 13 | 14 | 15 | + | C | 1 | 0 | 0 | 0 | 0 | 0 | 0 | 0 | 0 | 0 | 0 | 0 | 0 | 0 | 0 | 0 | 0 | 1 |
| H344 | 12 | 14 | 29 | 25 | 12 | 13 | 13 | 15 | - | C | 1 | 0 | 0 | 0 | 0 | 0 | 0 | 0 | 0 | 0 | 0 | 0 | 0 | 0 | 0 | 0 | 0 | 1 |
| H345 | 12 | 14 | 30 | 22 | 10 | 11 | 12 | 15 | + | C | 0 | 1 | 0 | 0 | 0 | 0 | 0 | 0 | 0 | 0 | 0 | 0 | 0 | 0 | 0 | 0 | 0 | 1 |
| H346 | 12 | 13 | 28 | 24 | 11 | 11 | 12 | 14 | + | C | 0 | 1 | 0 | 0 | 0 | 0 | 0 | 0 | 0 | 0 | 0 | 0 | 0 | 0 | 0 | 0 | 0 | 1 |
| H347 | 12 | 14 | 31 | 21 | 12 | 11 | 12 | 14 | + | C | 0 | 1 | 0 | 0 | 0 | 0 | 0 | 0 | 0 | 0 | 0 | 0 | 0 | 0 | 0 | 0 | 0 | 1 |
| H348 | 12 | 13 | 30 | 21 | 10 | 10 | 13 | 14 | + | C | 0 | 3 | 3 | 0 | 0 | 0 | 0 | 0 | 0 | 1 | 0 | 0 | 0 | 1 | 0 | 0 | 0 | 8 |
| H349 | 12 | 13 | 29 | 21 | 10 | 10 | 14 | 16 | + | C | 0 | 1 | 0 | 0 | 0 | 0 | 0 | 0 | 0 | 0 | 0 | 0 | 0 | 0 | 0 | 0 | 0 | 1 |
| H350 | 12 | 14 | 31 | 21 | 10 | 10 | 14 | 16 | + | C | 0 | 1 | 1 | 0 | 0 | 0 | 0 | 0 | 0 | 0 | 0 | 0 | 0 | 0 | 0 | 0 | 0 | 2 |

| | | | | | | | | | | | | | | | | | | | | | | | | | | | | |
|---|---|---|---|---|---|---|---|---|---|---|---|---|---|---|---|---|---|---|---|---|---|---|---|---|---|---|---|---|
| H351 | 12 | 13 | 30 | 21 | 11 | 10 | 12 | 14 | + | C | 0 | 2 | 0 | 0 | 0 | 0 | 0 | 0 | 0 | 1 | 0 | 0 | 0 | 0 | 0 | 0 | 0 | 3 |
| H352 | 13 | 14 | 30 | 22 | 10 | 11 | 12 | 15 | + | C | 0 | 1 | 0 | 0 | 0 | 0 | 0 | 0 | 0 | 0 | 0 | 0 | 0 | 0 | 0 | 0 | 0 | 1 |
| H353 | 12 | 14 | 29 | 25 | 11 | 13 | 12 | 14 | - | T | 0 | 1 | 0 | 0 | 0 | 0 | 0 | 0 | 0 | 0 | 0 | 0 | 0 | 0 | 0 | 0 | 0 | 1 |
| H354 | 14 | 15 | 30 | 24 | 11 | 13 | 12 | 14 | - | T | 0 | 1 | 0 | 0 | 0 | 0 | 0 | 0 | 0 | 0 | 0 | 0 | 0 | 0 | 0 | 0 | 0 | 1 |
| H355 | 12 | 13 | 30 | 21 | 10 | 10 | 14 | 15 | + | C | 0 | 2 | 1 | 0 | 0 | 0 | 0 | 0 | 0 | 0 | 0 | 0 | 0 | 1 | 0 | 0 | 0 | 4 |
| H356 | 12 | 13 | 29 | 22 | 10 | 10 | 12 | 15 | + | C | 0 | 1 | 0 | 0 | 0 | 0 | 0 | 0 | 0 | 0 | 0 | 0 | 0 | 0 | 0 | 0 | 0 | 1 |
| H357 | 12 | 13 | 30 | 21 | 10 | 10 | 13 | 16 | + | C | 0 | 1 | 5 | 0 | 0 | 0 | 0 | 0 | 0 | 3 | 0 | 0 | 0 | 4 | 0 | 0 | 0 | 13 |
| H358 | 13 | 12 | 30 | 21 | 10 | 10 | 14 | 15 | + | C | 0 | 1 | 0 | 0 | 0 | 0 | 0 | 0 | 0 | 0 | 0 | 0 | 0 | 0 | 0 | 0 | 0 | 1 |
| H359 | 12 | 14 | 31 | 21 | 11 | 11 | 12 | 15 | + | C | 0 | 2 | 0 | 0 | 0 | 0 | 0 | 0 | 0 | 0 | 0 | 0 | 0 | 0 | 0 | 0 | 0 | 2 |
| H360 | 12 | 13 | 32 | 21 | 10 | 11 | 12 | 15 | + | C | 0 | 1 | 0 | 0 | 0 | 0 | 0 | 0 | 0 | 0 | 0 | 0 | 0 | 0 | 0 | 0 | 0 | 1 |
| H361 | 12 | 14 | 31 | 21 | 10 | 11 | 12 | 16 | + | C | 0 | 1 | 0 | 0 | 0 | 0 | 0 | 0 | 0 | 0 | 0 | 0 | 0 | 0 | 0 | 0 | 0 | 1 |
| H362 | 11 | 14 | 29 | 21 | 10 | 11 | 13 | 16 | - | C | 0 | 1 | 0 | 0 | 0 | 0 | 0 | 0 | 0 | 0 | 0 | 0 | 0 | 0 | 0 | 0 | 0 | 1 |
| H363 | 12 | 14 | 30 | 21 | 10 | 11 | 13 | 15 | + | C | 0 | 1 | 0 | 0 | 0 | 0 | 0 | 0 | 0 | 0 | 0 | 0 | 0 | 0 | 0 | 0 | 0 | 1 |
| H364 | 12 | 14 | 32 | 21 | 11 | 11 | 11 | 15 | + | C | 0 | 1 | 0 | 0 | 0 | 0 | 0 | 0 | 0 | 0 | 0 | 0 | 0 | 0 | 0 | 0 | 0 | 1 |
| H365 | 12 | 13 | 29 | 24 | 10 | 13 | 12 | 14 | - | C | 0 | 1 | 0 | 0 | 0 | 0 | 0 | 0 | 0 | 0 | 0 | 0 | 0 | 0 | 0 | 0 | 0 | 1 |
| H366 | 12 | 14 | 32 | 21 | 10 | 11 | 13 | 16 | - | T | 0 | 1 | 0 | 0 | 0 | 0 | 0 | 0 | 0 | 0 | 0 | 0 | 0 | 0 | 0 | 0 | 0 | 1 |
| H367 | 12 | 15 | 31 | 24 | 11 | 13 | 12 | 14 | - | C | 0 | 1 | 0 | 0 | 0 | 0 | 0 | 0 | 0 | 0 | 0 | 0 | 0 | 0 | 0 | 0 | 0 | 1 |
| H368 | 12 | 14 | 30 | 21 | 10 | 11 | 12 | 17 | - | T | 0 | 1 | 0 | 0 | 0 | 0 | 0 | 0 | 0 | 0 | 0 | 0 | 0 | 0 | 0 | 0 | 0 | 1 |
| H369 | 12 | 14 | 30 | 21 | 11 | 11 | 13 | 16 | + | C | 0 | 1 | 0 | 0 | 0 | 0 | 0 | 0 | 0 | 0 | 0 | 0 | 0 | 0 | 0 | 0 | 0 | 1 |
| H370 | 12 | 14 | 31 | 21 | 10 | 11 | 12 | 15 | - | T | 0 | 1 | 0 | 0 | 0 | 0 | 0 | 0 | 0 | 0 | 0 | 0 | 0 | 0 | 0 | 0 | 0 | 1 |
| H371 | 12 | 13 | 31 | 21 | 10 | 11 | 12 | 14 | + | C | 0 | 1 | 0 | 0 | 0 | 0 | 0 | 0 | 0 | 0 | 0 | 0 | 0 | 0 | 0 | 0 | 0 | 1 |
| H372 | 12 | 14 | 30 | 23 | 10 | 13 | 12 | 14 | - | C | 0 | 1 | 0 | 0 | 0 | 0 | 0 | 0 | 0 | 0 | 0 | 0 | 0 | 0 | 0 | 0 | 0 | 1 |
| H373 | 12 | 15 | 31 | 21 | 10 | 11 | 12 | 15 | + | C | 0 | 1 | 0 | 0 | 0 | 0 | 0 | 0 | 0 | 0 | 0 | 0 | 0 | 0 | 0 | 0 | 0 | 1 |
| H374 | 12 | 15 | 32 | 21 | 10 | 11 | 12 | 16 | - | T | 0 | 1 | 0 | 0 | 0 | 0 | 0 | 0 | 0 | 0 | 0 | 0 | 0 | 0 | 0 | 0 | 0 | 1 |
| H375 | 12 | 14 | 30 | 24 | 11 | 13 | 12 | 14 | - | C | 0 | 1 | 0 | 0 | 0 | 0 | 0 | 0 | 0 | 0 | 0 | 0 | 0 | 0 | 0 | 0 | 0 | 1 |
| H376 | 12 | 14 | 31 | 21 | 10 | 11 | 13 | 15 | + | C | 0 | 1 | 0 | 0 | 0 | 0 | 0 | 0 | 0 | 0 | 0 | 0 | 0 | 0 | 0 | 0 | 0 | 1 |
| H377 | 12 | 14 | 30 | 24 | 11 | 13 | 12 | 14 | - | T | 0 | 1 | 0 | 0 | 0 | 0 | 0 | 0 | 0 | 0 | 0 | 0 | 0 | 0 | 0 | 0 | 0 | 1 |
| H378 | 12 | 14 | 30 | 21 | 10 | 11 | 12 | 17 | + | C | 0 | 1 | 0 | 0 | 0 | 0 | 0 | 0 | 0 | 0 | 0 | 0 | 0 | 0 | 0 | 0 | 0 | 1 |
| H379 | 12 | 13 | 31 | 21 | 10 | 11 | 13 | 17 | - | T | 0 | 1 | 0 | 0 | 0 | 0 | 0 | 0 | 0 | 0 | 0 | 0 | 0 | 0 | 0 | 0 | 0 | 1 |
| H380 | 16 | 13 | 28 | 22 | 10 | 11 | 12 | 14 | - | C | 0 | 1 | 0 | 0 | 0 | 0 | 0 | 0 | 0 | 0 | 0 | 0 | 0 | 0 | 0 | 0 | 0 | 1 |
| H381 | 12 | 14 | 29 | 24 | 11 | 13 | 12 | 14 | - | C | 0 | 1 | 0 | 0 | 0 | 0 | 0 | 0 | 0 | 0 | 0 | 0 | 0 | 0 | 0 | 0 | 0 | 1 |
| H382 | 9 | 13 | 32 | 23 | 10 | 11 | 13 | 15 | - | T | 0 | 1 | 0 | 0 | 0 | 0 | 0 | 0 | 0 | 0 | 0 | 0 | 0 | 0 | 0 | 0 | 0 | 1 |
| H383 | 12 | 14 | 31 | 21 | 10 | 11 | 12 | 15 | + | C | 0 | 1 | 0 | 0 | 0 | 0 | 0 | 0 | 0 | 0 | 0 | 0 | 0 | 0 | 0 | 0 | 0 | 1 |
| H384 | 9 | 13 | 31 | 25 | 11 | 10 | 12 | 14 | - | C | 0 | 1 | 0 | 0 | 0 | 0 | 0 | 0 | 0 | 0 | 0 | 0 | 0 | 0 | 0 | 0 | 0 | 1 |
| H385 | 14 | 13 | 29 | 25 | 11 | 12 | 11 | 13 | - | C | 0 | 1 | 0 | 0 | 0 | 0 | 0 | 0 | 0 | 0 | 0 | 0 | 0 | 0 | 0 | 0 | 0 | 1 |
| H386 | 12 | 13 | 29 | 24 | 11 | 12 | 12 | 13 | - | T | 0 | 1 | 0 | 0 | 0 | 0 | 0 | 0 | 0 | 5 | 0 | 0 | 0 | 3 | 0 | 0 | 0 | 9 |
| H387 | 12 | 12 | 29 | 21 | 10 | 10 | 14 | 16 | - | T | 0 | 1 | 0 | 0 | 0 | 0 | 0 | 0 | 0 | 0 | 0 | 0 | 0 | 0 | 0 | 0 | 0 | 1 |
| H388 | 9 | 13 | 31 | 24 | 10 | 10 | 12 | 15 | - | T | 0 | 1 | 0 | 0 | 0 | 0 | 0 | 0 | 0 | 0 | 0 | 0 | 0 | 0 | 0 | 0 | 0 | 1 |
| H389 | 12 | 13 | 29 | 24 | 11 | 12 | 12 | 13 | - | C | 0 | 1 | 0 | 0 | 0 | 0 | 0 | 0 | 0 | 0 | 0 | 0 | 0 | 0 | 0 | 0 | 0 | 1 |
| H390 | 12 | 13 | 31 | 21 | 10 | 10 | 13 | 15 | + | C | 0 | 1 | 1 | 0 | 0 | 0 | 0 | 0 | 0 | 0 | 0 | 0 | 0 | 3 | 0 | 0 | 0 | 5 |
| H391 | 12 | 13 | 32 | 21 | 10 | 10 | 14 | 16 | - | T | 0 | 1 | 0 | 0 | 0 | 0 | 0 | 0 | 0 | 0 | 0 | 0 | 0 | 0 | 0 | 0 | 0 | 1 |
| H392 | 13 | 13 | 30 | 21 | 10 | 10 | 12 | 14 | + | C | 0 | 1 | 3 | 0 | 0 | 0 | 0 | 0 | 0 | 0 | 0 | 0 | 0 | 0 | 0 | 0 | 0 | 4 |
| H393 | 12 | 13 | 30 | 21 | 11 | 10 | 12 | 15 | + | C | 0 | 1 | 0 | 0 | 0 | 0 | 0 | 0 | 0 | 0 | 0 | 0 | 0 | 0 | 0 | 0 | 0 | 1 |
| H394 | 12 | 14 | 32 | 21 | 10 | 10 | 13 | 14 | + | C | 0 | 1 | 0 | 0 | 0 | 0 | 0 | 0 | 0 | 0 | 0 | 0 | 0 | 0 | 0 | 0 | 0 | 1 |
| H395 | 12 | 12 | 28 | 26 | 11 | 10 | 12 | 13 | + | C | 0 | 1 | 0 | 0 | 0 | 0 | 0 | 0 | 0 | 0 | 0 | 0 | 0 | 0 | 0 | 0 | 0 | 1 |
| H396 | 12 | 13 | 30 | 22 | 10 | 10 | 12 | 14 | + | C | 0 | 1 | 0 | 0 | 0 | 0 | 0 | 0 | 0 | 0 | 0 | 0 | 0 | 0 | 0 | 0 | 0 | 1 |
| H397 | 12 | 13 | 31 | 21 | 10 | 10 | 12 | 14 | + | C | 0 | 3 | 7 | 0 | 0 | 0 | 0 | 0 | 0 | 2 | 0 | 0 | 0 | 5 | 0 | 0 | 0 | 17 |
| H398 | 12 | 12 | 28 | 22 | 10 | 10 | 11 | 12 | - | T | 0 | 1 | 0 | 0 | 0 | 0 | 0 | 0 | 0 | 0 | 0 | 0 | 0 | 0 | 0 | 0 | 0 | 1 |
| H399 | 12 | 13 | 29 | 25 | 11 | 10 | 12 | 13 | + | C | 0 | 1 | 0 | 0 | 0 | 0 | 0 | 0 | 0 | 0 | 0 | 0 | 0 | 0 | 0 | 0 | 0 | 1 |
| H400 | 12 | 13 | 31 | 21 | 10 | 10 | 14 | 16 | + | C | 0 | 2 | 0 | 0 | 0 | 0 | 0 | 0 | 0 | 0 | 0 | 0 | 0 | 0 | 0 | 0 | 0 | 2 |
| H401 | 12 | 13 | 31 | 22 | 10 | 10 | 13 | 14 | + | C | 0 | 1 | 0 | 0 | 0 | 0 | 0 | 0 | 0 | 1 | 0 | 0 | 0 | 0 | 0 | 0 | 0 | 2 |
| H402 | 12 | 13 | 29 | 24 | 10 | 10 | 12 | 13 | + | C | 0 | 1 | 0 | 0 | 0 | 0 | 0 | 0 | 0 | 0 | 0 | 0 | 0 | 0 | 0 | 0 | 0 | 1 |
| H403 | 12 | 13 | 29 | 23 | 11 | 12 | 12 | 13 | - | C | 0 | 1 | 0 | 0 | 0 | 0 | 0 | 0 | 0 | 0 | 0 | 0 | 0 | 0 | 0 | 0 | 0 | 1 |
| H404 | 12 | 13 | 30 | 21 | 10 | 10 | 12 | 15 | + | C | 0 | 1 | 3 | 0 | 0 | 0 | 0 | 0 | 0 | 2 | 0 | 0 | 0 | 2 | 0 | 0 | 0 | 8 |
| H405 | 12 | 13 | 30 | 25 | 10 | 12 | 12 | 13 | - | C | 0 | 1 | 0 | 0 | 0 | 0 | 0 | 0 | 0 | 0 | 0 | 0 | 0 | 0 | 0 | 0 | 0 | 1 |
| H406 | 12 | 13 | 31 | 24 | 10 | 10 | 12 | 12 | - | T | 0 | 1 | 0 | 0 | 0 | 0 | 0 | 0 | 0 | 0 | 0 | 0 | 0 | 0 | 0 | 0 | 0 | 1 |
| H407 | 12 | 13 | 30 | 21 | 10 | 10 | 13 | 14 | - | T | 0 | 1 | 0 | 0 | 0 | 0 | 0 | 0 | 0 | 0 | 0 | 0 | 0 | 0 | 0 | 0 | 0 | 1 |
| H408 | 12 | 13 | 30 | 24 | 10 | 10 | 12 | 14 | - | C | 0 | 1 | 0 | 0 | 0 | 0 | 0 | 0 | 0 | 0 | 0 | 0 | 0 | 0 | 0 | 0 | 0 | 1 |
| H409 | 12 | 12 | 29 | 21 | 10 | 10 | 13 | 15 | - | C | 0 | 1 | 0 | 0 | 0 | 0 | 0 | 0 | 0 | 0 | 0 | 0 | 0 | 0 | 0 | 0 | 0 | 1 |

| | | | | | | | | | | | | | | | | | | | | | | | | | | | | |
|---|---|---|---|---|---|---|---|---|---|---|---|---|---|---|---|---|---|---|---|---|---|---|---|---|---|---|---|---|
| H410 | 12 | 13 | 28 | 25 | 10 | 14 | 12 | 14 | - | T | 0 | 1 | 0 | 0 | 0 | 0 | 0 | 0 | 0 | 0 | 0 | 0 | 0 | 0 | 0 | 0 | 0 | 1 |
| H411 | 12 | 15 | 32 | 21 | 10 | 11 | 12 | 16 | + | C | 0 | 1 | 0 | 0 | 0 | 0 | 0 | 0 | 0 | 0 | 0 | 0 | 0 | 0 | 0 | 0 | 0 | 1 |
| H412 | 12 | 14 | 30 | 21 | 10 | 11 | 14 | 16 | + | C | 0 | 1 | 0 | 0 | 0 | 0 | 0 | 0 | 0 | 0 | 0 | 0 | 0 | 0 | 0 | 0 | 0 | 1 |
| H413 | 12 | 14 | 31 | 21 | 10 | 11 | 12 | 17 | + | C | 0 | 1 | 0 | 0 | 0 | 0 | 0 | 0 | 0 | 0 | 0 | 0 | 0 | 0 | 0 | 0 | 0 | 1 |
| H414 | 12 | 14 | 31 | 21 | 10 | 12 | 12 | 15 | + | C | 0 | 1 | 0 | 0 | 0 | 0 | 0 | 0 | 0 | 0 | 0 | 0 | 0 | 0 | 0 | 0 | 0 | 1 |
| H415 | 12 | 14 | 29 | 26 | 10 | 12 | 13 | 14 | + | C | 0 | 1 | 0 | 0 | 0 | 0 | 0 | 0 | 0 | 0 | 0 | 0 | 0 | 0 | 0 | 0 | 0 | 1 |
| H416 | 13 | 13 | 29 | 22 | 10 | 11 | 13 | 15 | - | T | 0 | 1 | 0 | 0 | 0 | 0 | 0 | 0 | 0 | 0 | 0 | 0 | 0 | 0 | 0 | 0 | 0 | 1 |
| H417 | 12 | 14 | 31 | 21 | 10 | 11 | 14 | 16 | + | C | 0 | 1 | 0 | 0 | 0 | 0 | 0 | 0 | 0 | 0 | 0 | 0 | 0 | 0 | 0 | 0 | 0 | 1 |
| H418 | 12 | 14 | 32 | 21 | 10 | 11 | 14 | 15 | + | C | 0 | 2 | 0 | 0 | 0 | 0 | 0 | 0 | 0 | 0 | 0 | 0 | 0 | 0 | 0 | 0 | 0 | 2 |
| H419 | 14 | 13 | 29 | 22 | 11 | 11 | 12 | 14 | - | T | 0 | 1 | 0 | 0 | 0 | 0 | 0 | 0 | 0 | 0 | 0 | 0 | 0 | 0 | 0 | 0 | 0 | 1 |
| H420 | 12 | 14 | 30 | 21 | 11 | 11 | 12 | 15 | + | C | 0 | 1 | 0 | 0 | 0 | 0 | 0 | 0 | 0 | 0 | 0 | 0 | 0 | 0 | 0 | 0 | 0 | 1 |
| H421 | 12 | 14 | 29 | 24 | 11 | 14 | 12 | 14 | - | T | 0 | 1 | 0 | 0 | 0 | 0 | 0 | 0 | 0 | 0 | 0 | 0 | 0 | 0 | 0 | 0 | 0 | 1 |
| H422 | 12 | 13 | 30 | 21 | 11 | 11 | 12 | 14 | + | C | 0 | 1 | 0 | 0 | 0 | 0 | 0 | 0 | 0 | 0 | 0 | 0 | 0 | 0 | 0 | 0 | 0 | 1 |
| H423 | 12 | 13 | 32 | 21 | 10 | 11 | 12 | 14 | + | C | 0 | 2 | 0 | 0 | 0 | 0 | 0 | 0 | 0 | 0 | 0 | 0 | 0 | 0 | 0 | 0 | 0 | 2 |
| H424 | 13 | 11 | 28 | 22 | 10 | 10 | 12 | 14 | + | C | 0 | 1 | 0 | 0 | 0 | 0 | 0 | 0 | 0 | 0 | 0 | 0 | 0 | 0 | 0 | 0 | 0 | 1 |
| H425 | 12 | 13 | 30 | 21 | 10 | 11 | 12 | 16 | + | C | 0 | 1 | 0 | 0 | 0 | 1 | 0 | 0 | 0 | 0 | 0 | 0 | 0 | 0 | 0 | 0 | 0 | 2 |
| H426 | 12 | 14 | 31 | 20 | 10 | 11 | 12 | 14 | + | C | 0 | 1 | 0 | 0 | 0 | 0 | 0 | 0 | 0 | 0 | 0 | 0 | 0 | 0 | 0 | 0 | 0 | 1 |
| H427 | 12 | 13 | 30 | 24 | 10 | 10 | 12 | 14 | - | T | 0 | 1 | 0 | 0 | 0 | 0 | 0 | 0 | 0 | 0 | 0 | 0 | 0 | 0 | 0 | 0 | 0 | 1 |
| H428 | 12 | 14 | 30 | 24 | 11 | 11 | 11 | 13 | - | T | 0 | 1 | 0 | 0 | 0 | 0 | 0 | 0 | 0 | 0 | 0 | 0 | 0 | 0 | 0 | 0 | 0 | 1 |
| H429 | 13 | 13 | 31 | 23 | 10 | 10 | 13 | 13 | - | T | 0 | 1 | 0 | 0 | 0 | 0 | 0 | 0 | 0 | 0 | 0 | 0 | 0 | 0 | 0 | 0 | 0 | 1 |
| H430 | 14 | 13 | 29 | 22 | 10 | 10 | 12 | 13 | - | T | 0 | 1 | 0 | 0 | 0 | 0 | 0 | 0 | 0 | 0 | 0 | 0 | 0 | 0 | 0 | 0 | 0 | 1 |
| H431 | 12 | 13 | 31 | 21 | 10 | 11 | 13 | 15 | + | C | 0 | 1 | 0 | 0 | 0 | 0 | 0 | 0 | 0 | 0 | 0 | 0 | 0 | 0 | 0 | 0 | 0 | 1 |
| H432 | 12 | 15 | 31 | 24 | 11 | 11 | 12 | 13 | - | T | 0 | 1 | 0 | 0 | 0 | 0 | 0 | 0 | 0 | 0 | 0 | 0 | 0 | 0 | 0 | 0 | 0 | 1 |
| H433 | 12 | 13 | 30 | 21 | 10 | 10 | 12 | 16 | + | C | 0 | 1 | 0 | 0 | 0 | 0 | 0 | 0 | 0 | 2 | 0 | 0 | 0 | 2 | 0 | 0 | 0 | 5 |
| H434 | 12 | 13 | 29 | 23 | 11 | 10 | 12 | 13 | - | T | 0 | 1 | 0 | 0 | 0 | 0 | 0 | 0 | 0 | 0 | 0 | 0 | 0 | 0 | 0 | 0 | 0 | 1 |
| H435 | 12 | 14 | 30 | 23 | 11 | 10 | 12 | 13 | - | C | 0 | 1 | 0 | 0 | 0 | 0 | 0 | 0 | 0 | 0 | 0 | 0 | 0 | 0 | 0 | 0 | 0 | 1 |
| H436 | 12 | 13 | 30 | 24 | 10 | 11 | 12 | 14 | - | T | 0 | 1 | 0 | 0 | 0 | 0 | 0 | 0 | 0 | 0 | 0 | 0 | 0 | 0 | 0 | 0 | 0 | 1 |
| H437 | 12 | 12 | 29 | 21 | 10 | 11 | 13 | 15 | + | C | 0 | 1 | 0 | 0 | 0 | 0 | 0 | 0 | 0 | 0 | 0 | 0 | 0 | 0 | 0 | 0 | 0 | 1 |
| H438 | 12 | 13 | 29 | 23 | 12 | 10 | 12 | 13 | - | T | 0 | 1 | 0 | 0 | 0 | 0 | 0 | 0 | 0 | 0 | 0 | 0 | 0 | 0 | 0 | 0 | 0 | 1 |
| H439 | 12 | 13 | 30 | 21 | 10 | 12 | 13 | 16 | + | C | 0 | 0 | 1 | 0 | 0 | 0 | 0 | 0 | 0 | 0 | 0 | 0 | 0 | 0 | 0 | 0 | 0 | 1 |
| H440 | 12 | 14 | 31 | 21 | 10 | 10 | 13 | 16 | + | C | 0 | 0 | 1 | 0 | 0 | 0 | 0 | 0 | 0 | 0 | 0 | 0 | 0 | 0 | 0 | 0 | 0 | 1 |
| H441 | 12 | 12 | 31 | 21 | 10 | 10 | 13 | 16 | + | C | 0 | 0 | 1 | 0 | 0 | 0 | 0 | 0 | 0 | 0 | 0 | 0 | 0 | 0 | 0 | 0 | 0 | 1 |
| H442 | 13 | 13 | 31 | 21 | 10 | 10 | 12 | 14 | + | C | 0 | 0 | 5 | 0 | 0 | 0 | 0 | 0 | 0 | 0 | 0 | 0 | 0 | 0 | 0 | 0 | 0 | 5 |
| H443 | 12 | 13 | 32 | 24 | 10 | 10 | 12 | 14 | - | C | 0 | 0 | 1 | 0 | 0 | 0 | 0 | 0 | 0 | 0 | 0 | 0 | 0 | 0 | 0 | 0 | 0 | 1 |
| H444 | 12 | 13 | 30 | 22 | 10 | 10 | 14 | 15 | + | C | 0 | 0 | 1 | 0 | 0 | 0 | 0 | 0 | 0 | 0 | 0 | 0 | 0 | 0 | 0 | 0 | 0 | 1 |
| H445 | 13 | 13 | 32 | 21 | 10 | 10 | 12 | 13 | + | C | 0 | 0 | 2 | 0 | 0 | 0 | 0 | 0 | 0 | 0 | 0 | 0 | 0 | 0 | 0 | 0 | 0 | 2 |
| H446 | 12 | 13 | 30 | 21 | 10 | 10 | 13 | 15 | + | C | 0 | 0 | 6 | 0 | 0 | 0 | 0 | 0 | 0 | 3 | 0 | 0 | 0 | 3 | 0 | 0 | 0 | 12 |
| H447 | 12 | 13 | 31 | 21 | 10 | 10 | 13 | 16 | + | C | 0 | 0 | 2 | 0 | 0 | 0 | 0 | 0 | 0 | 0 | 0 | 0 | 0 | 1 | 0 | 0 | 0 | 3 |
| H448 | 12 | 14 | 31 | 21 | 10 | 10 | 13 | 14 | + | C | 0 | 0 | 1 | 0 | 0 | 0 | 0 | 0 | 0 | 0 | 0 | 0 | 0 | 1 | 0 | 0 | 0 | 2 |
| H449 | 12 | 13 | 30 | 21 | 10 | 10 | 14 | 14 | + | C | 0 | 0 | 2 | 0 | 0 | 0 | 0 | 0 | 0 | 1 | 0 | 0 | 0 | 3 | 0 | 0 | 0 | 6 |
| H450 | 12 | 14 | 32 | 21 | 10 | 10 | 14 | 15 | + | C | 0 | 0 | 2 | 0 | 0 | 0 | 0 | 0 | 0 | 0 | 0 | 0 | 0 | 0 | 0 | 0 | 0 | 2 |
| H451 | 12 | 13 | 30 | 21 | 11 | 10 | 13 | 15 | + | C | 0 | 0 | 2 | 0 | 0 | 0 | 0 | 0 | 0 | 0 | 0 | 0 | 0 | 0 | 0 | 0 | 0 | 2 |
| H452 | 12 | 13 | 30 | 22 | 10 | 10 | 14 | 14 | + | C | 0 | 0 | 1 | 0 | 0 | 0 | 0 | 0 | 0 | 0 | 0 | 0 | 0 | 0 | 0 | 0 | 0 | 1 |
| H453 | 12 | 13 | 31 | 21 | 9 | 10 | 13 | 14 | + | C | 0 | 0 | 1 | 0 | 0 | 0 | 0 | 0 | 0 | 0 | 0 | 0 | 0 | 0 | 0 | 0 | 0 | 1 |
| H454 | 12 | 12 | 30 | 21 | 10 | 10 | 12 | 14 | + | C | 0 | 0 | 3 | 0 | 0 | 0 | 0 | 0 | 0 | 0 | 0 | 0 | 0 | 0 | 0 | 0 | 0 | 3 |
| H455 | 12 | 13 | 30 | 21 | 11 | 10 | 13 | 14 | + | C | 0 | 0 | 1 | 0 | 0 | 0 | 0 | 0 | 0 | 0 | 0 | 0 | 0 | 1 | 0 | 0 | 0 | 2 |
| H456 | 12 | 13 | 31 | 21 | 10 | 10 | 12 | 15 | + | C | 0 | 0 | 4 | 0 | 0 | 0 | 0 | 0 | 0 | 0 | 0 | 0 | 0 | 2 | 0 | 0 | 0 | 6 |
| H457 | 12 | 13 | 30 | 21 | 10 | 10 | 15 | 15 | + | C | 0 | 0 | 3 | 0 | 0 | 0 | 0 | 0 | 0 | 0 | 0 | 0 | 0 | 1 | 0 | 0 | 0 | 4 |
| H458 | 12 | 13 | 30 | 21 | 10 | 10 | 14 | 16 | + | C | 0 | 0 | 1 | 0 | 0 | 0 | 0 | 0 | 0 | 1 | 0 | 0 | 0 | 2 | 0 | 0 | 0 | 4 |
| H459 | 12 | 11 | 28 | 21 | 10 | 10 | 12 | 14 | + | C | 0 | 0 | 1 | 0 | 0 | 0 | 0 | 0 | 0 | 0 | 0 | 0 | 0 | 0 | 0 | 0 | 0 | 1 |
| H460 | 12 | 11 | 28 | 22 | 10 | 10 | 11 | 15 | + | C | 0 | 0 | 1 | 0 | 0 | 0 | 0 | 0 | 0 | 0 | 0 | 0 | 0 | 0 | 0 | 0 | 0 | 1 |
| H461 | 12 | 14 | 31 | 21 | 10 | 10 | 13 | 15 | + | C | 0 | 0 | 1 | 0 | 0 | 0 | 0 | 0 | 0 | 0 | 0 | 0 | 0 | 0 | 0 | 0 | 0 | 1 |
| H462 | 12 | 14 | 32 | 21 | 10 | 10 | 12 | 14 | + | C | 0 | 0 | 2 | 0 | 0 | 0 | 0 | 0 | 0 | 0 | 0 | 0 | 0 | 0 | 0 | 0 | 0 | 2 |
| H463 | 12 | 13 | 32 | 21 | 10 | 10 | 15 | 15 | + | C | 0 | 0 | 1 | 0 | 0 | 0 | 0 | 0 | 0 | 0 | 0 | 0 | 0 | 0 | 0 | 0 | 0 | 1 |
| H464 | 12 | 13 | 31 | 21 | 10 | 10 | 13 | 14 | + | C | 0 | 0 | 1 | 0 | 0 | 0 | 0 | 0 | 0 | 2 | 0 | 0 | 0 | 1 | 0 | 0 | 0 | 4 |
| H465 | 12 | 13 | 30 | 21 | 9 | 10 | 13 | 14 | + | C | 0 | 0 | 1 | 0 | 0 | 0 | 0 | 0 | 0 | 0 | 0 | 0 | 0 | 0 | 0 | 0 | 0 | 1 |
| H466 | 12 | 12 | 28 | 22 | 10 | 12 | 12 | 15 | - | C | 0 | 0 | 1 | 0 | 0 | 0 | 0 | 0 | 0 | 0 | 0 | 0 | 0 | 0 | 0 | 0 | 0 | 1 |
| H467 | 12 | 12 | 28 | 25 | 10 | 10 | 12 | 15 | + | C | 0 | 0 | 1 | 0 | 0 | 0 | 0 | 0 | 0 | 0 | 0 | 0 | 0 | 0 | 0 | 0 | 0 | 1 |
| H468 | 12 | 14 | 30 | 25 | 11 | 10 | 12 | 14 | - | C | 0 | 0 | 2 | 0 | 0 | 0 | 0 | 0 | 0 | 0 | 0 | 0 | 0 | 0 | 0 | 0 | 0 | 2 |

| | | | | | | | | | | | | | | | | | | | | | | | | | | | | |
|---|---|---|---|---|---|---|---|---|---|---|---|---|---|---|---|---|---|---|---|---|---|---|---|---|---|---|---|---|
| H469 | 12 | 14 | 30 | 24 | 10 | 10 | 12 | 15 | - | C | 0 | 0 | 1 | 0 | 0 | 0 | 0 | 0 | 0 | 0 | 0 | 0 | 0 | 0 | 0 | 0 | 0 | 1 |
| H470 | 12 | 14 | 32 | 21 | 10 | 10 | 12 | 15 | + | C | 0 | 0 | 1 | 0 | 0 | 0 | 0 | 0 | 0 | 0 | 0 | 0 | 0 | 0 | 0 | 0 | 0 | 1 |
| H471 | 12 | 14 | 32 | 20 | 10 | 10 | 12 | 14 | + | C | 0 | 0 | 1 | 0 | 0 | 0 | 0 | 0 | 0 | 0 | 0 | 0 | 0 | 0 | 0 | 0 | 0 | 1 |
| H472 | 12 | 13 | 31 | 21 | 11 | 10 | 12 | 14 | + | C | 0 | 0 | 1 | 0 | 0 | 0 | 0 | 0 | 0 | 2 | 0 | 0 | 0 | 2 | 0 | 0 | 0 | 5 |
| H473 | 12 | 13 | 31 | 21 | 11 | 12 | 14 | 17 | + | C | 0 | 0 | 0 | 1 | 0 | 0 | 0 | 2 | 0 | 0 | 0 | 0 | 0 | 0 | 0 | 0 | 0 | 3 |
| H474 | 12 | 14 | 32 | 21 | 11 | 12 | 15 | 17 | + | C | 0 | 0 | 0 | 1 | 0 | 0 | 0 | 0 | 0 | 0 | 0 | 0 | 0 | 0 | 0 | 0 | 0 | 1 |
| H475 | 12 | 14 | 31 | 21 | 11 | 12 | 15 | 16 | + | C | 0 | 0 | 0 | 4 | 0 | 0 | 0 | 0 | 0 | 0 | 0 | 0 | 1 | 0 | 1 | 4 | 1 | 11 |
| H476 | 12 | 14 | 32 | 21 | 11 | 12 | 14 | 17 | + | C | 0 | 0 | 0 | 2 | 0 | 0 | 0 | 0 | 0 | 0 | 0 | 0 | 0 | 0 | 0 | 0 | 0 | 2 |
| H477 | 12 | 13 | 32 | 21 | 11 | 12 | 14 | 17 | + | C | 0 | 0 | 0 | 1 | 0 | 0 | 0 | 0 | 0 | 0 | 0 | 0 | 0 | 0 | 0 | 0 | 0 | 1 |
| H478 | 12 | 14 | 31 | 21 | 11 | 11 | 13 | 16 | + | C | 0 | 0 | 0 | 1 | 0 | 1 | 0 | 0 | 0 | 0 | 0 | 0 | 0 | 0 | 0 | 0 | 0 | 2 |
| H479 | 12 | 13 | 30 | 21 | 11 | 11 | 13 | 15 | + | C | 0 | 0 | 0 | 1 | 0 | 0 | 0 | 1 | 0 | 0 | 0 | 1 | 0 | 0 | 0 | 1 | 1 | 5 |
| H480 | 12 | 14 | 31 | 21 | 11 | 13 | 13 | 17 | + | C | 0 | 0 | 0 | 1 | 0 | 0 | 0 | 0 | 0 | 0 | 0 | 0 | 0 | 0 | 0 | 0 | 0 | 1 |
| H481 | 12 | 14 | 32 | 21 | 11 | 12 | 13 | 17 | + | C | 0 | 0 | 0 | 1 | 0 | 0 | 0 | 0 | 0 | 0 | 0 | 0 | 0 | 0 | 0 | 0 | 0 | 1 |
| H482 | 12 | 14 | 31 | 22 | 12 | 12 | 13 | 17 | + | C | 0 | 0 | 0 | 1 | 0 | 0 | 0 | 0 | 0 | 0 | 0 | 0 | 0 | 0 | 0 | 0 | 0 | 1 |
| H483 | 12 | 14 | 33 | 21 | 11 | 12 | 14 | 17 | + | C | 0 | 0 | 0 | 1 | 0 | 0 | 0 | 0 | 0 | 0 | 0 | 0 | 0 | 0 | 0 | 0 | 0 | 1 |
| H484 | 12 | 14 | 31 | 21 | 11 | 12 | 14 | 17 | - | C | 0 | 0 | 0 | 1 | 0 | 0 | 0 | 0 | 0 | 0 | 0 | 0 | 0 | 0 | 0 | 0 | 0 | 1 |
| H485 | 12 | 14 | 31 | 21 | 11 | 12 | 15 | 17 | + | C | 0 | 0 | 0 | 1 | 0 | 0 | 0 | 0 | 1 | 0 | 0 | 0 | 0 | 0 | 0 | 0 | 0 | 2 |
| H486 | 12 | 12 | 31 | 21 | 11 | 12 | 14 | 17 | + | C | 0 | 0 | 0 | 1 | 0 | 0 | 0 | 1 | 0 | 0 | 0 | 0 | 0 | 0 | 0 | 0 | 0 | 2 |
| H487 | 12 | 14 | 31 | 21 | 12 | 12 | 15 | 17 | + | C | 0 | 0 | 0 | 1 | 0 | 0 | 0 | 0 | 0 | 0 | 0 | 0 | 0 | 0 | 0 | 0 | 0 | 1 |
| H488 | 12 | 14 | 30 | 21 | 12 | 12 | 13 | 15 | + | C | 0 | 0 | 0 | 1 | 0 | 0 | 0 | 0 | 0 | 0 | 0 | 0 | 0 | 0 | 0 | 0 | 0 | 1 |
| H489 | 13 | 12 | 29 | 21 | 11 | 12 | 12 | 15 | + | C | 0 | 0 | 0 | 1 | 0 | 0 | 0 | 0 | 0 | 0 | 0 | 0 | 0 | 0 | 0 | 0 | 0 | 1 |
| H490 | 12 | 15 | 31 | 21 | 11 | 12 | 15 | 16 | + | C | 0 | 0 | 0 | 2 | 0 | 0 | 0 | 0 | 0 | 0 | 0 | 0 | 0 | 0 | 0 | 0 | 0 | 2 |
| H491 | 12 | 13 | 30 | 20 | 11 | 12 | 11 | 16 | + | C | 0 | 0 | 0 | 1 | 0 | 0 | 0 | 0 | 0 | 0 | 0 | 0 | 0 | 0 | 0 | 0 | 0 | 1 |
| H492 | 12 | 15 | 33 | 21 | 12 | 12 | 12 | 15 | + | C | 0 | 0 | 0 | 1 | 0 | 0 | 0 | 0 | 0 | 0 | 0 | 0 | 0 | 0 | 0 | 0 | 0 | 1 |
| H493 | 12 | 14 | 30 | 21 | 11 | 12 | 14 | 16 | + | C | 0 | 0 | 0 | 2 | 0 | 0 | 0 | 0 | 0 | 0 | 0 | 0 | 1 | 0 | 0 | 0 | 0 | 3 |
| H494 | 12 | 14 | 33 | 21 | 12 | 12 | 11 | 15 | + | C | 0 | 0 | 0 | 1 | 0 | 0 | 0 | 0 | 0 | 0 | 0 | 0 | 0 | 0 | 0 | 0 | 0 | 1 |
| H495 | 12 | 13 | 30 | 24 | 11 | 14 | 12 | 14 | - | T | 0 | 0 | 0 | 0 | 1 | 0 | 0 | 0 | 0 | 0 | 0 | 0 | 0 | 0 | 0 | 0 | 0 | 1 |
| H496 | 12 | 14 | 30 | 24 | 11 | 14 | 11 | 14 | - | T | 0 | 0 | 0 | 0 | 1 | 0 | 0 | 0 | 0 | 0 | 0 | 0 | 0 | 0 | 0 | 0 | 0 | 1 |
| H497 | 13 | 13 | 32 | 24 | 12 | 11 | 13 | 16 | - | T | 0 | 0 | 0 | 0 | 1 | 0 | 0 | 0 | 0 | 0 | 0 | 0 | 0 | 0 | 0 | 0 | 0 | 1 |
| H498 | 12 | 13 | 30 | 24 | 12 | 13 | 12 | 14 | - | T | 0 | 0 | 0 | 0 | 3 | 0 | 0 | 0 | 0 | 0 | 0 | 0 | 0 | 0 | 0 | 0 | 0 | 3 |
| H499 | 12 | 13 | 28 | 25 | 11 | 14 | 11 | 14 | - | T | 0 | 0 | 0 | 0 | 1 | 0 | 0 | 0 | 0 | 0 | 0 | 0 | 0 | 0 | 0 | 0 | 0 | 1 |
| H500 | 12 | 14 | 30 | 25 | 12 | 13 | 12 | 14 | - | T | 0 | 0 | 0 | 0 | 1 | 0 | 0 | 0 | 0 | 0 | 0 | 0 | 0 | 0 | 0 | 0 | 0 | 1 |
| H501 | 12 | 14 | 31 | 24 | 11 | 13 | 13 | 13 | - | T | 0 | 0 | 0 | 0 | 1 | 0 | 0 | 0 | 0 | 0 | 0 | 0 | 0 | 0 | 0 | 0 | 0 | 1 |
| H502 | 12 | 13 | 29 | 24 | 12 | 12 | 11 | 14 | - | C | 0 | 0 | 0 | 0 | 1 | 0 | 0 | 0 | 0 | 0 | 0 | 0 | 0 | 0 | 0 | 0 | 0 | 1 |
| H503 | 12 | 13 | 29 | 22 | 11 | 11 | 12 | 13 | - | T | 0 | 0 | 0 | 0 | 1 | 0 | 0 | 0 | 0 | 0 | 0 | 0 | 0 | 0 | 0 | 0 | 0 | 1 |
| H504 | 12 | 13 | 30 | 24 | 11 | 13 | 12 | 13 | - | T | 0 | 0 | 0 | 0 | 1 | 0 | 1 | 0 | 0 | 0 | 0 | 0 | 0 | 0 | 0 | 0 | 0 | 2 |
| H505 | 12 | 13 | 30 | 24 | 11 | 12 | 12 | 13 | - | T | 0 | 0 | 0 | 0 | 1 | 0 | 0 | 0 | 0 | 2 | 0 | 0 | 0 | 0 | 0 | 0 | 0 | 3 |
| H506 | 12 | 13 | 29 | 23 | 13 | 13 | 12 | 14 | - | C | 0 | 0 | 0 | 0 | 1 | 0 | 0 | 0 | 0 | 0 | 0 | 0 | 0 | 0 | 0 | 0 | 0 | 1 |
| H507 | 12 | 13 | 30 | 23 | 10 | 11 | 11 | 15 | - | T | 0 | 0 | 0 | 0 | 1 | 0 | 0 | 0 | 0 | 0 | 0 | 0 | 0 | 0 | 0 | 0 | 0 | 1 |
| H508 | 12 | 14 | 29 | 23 | 11 | 11 | 12 | 15 | - | T | 0 | 0 | 0 | 0 | 1 | 0 | 0 | 0 | 0 | 0 | 0 | 0 | 0 | 0 | 0 | 0 | 0 | 1 |
| H509 | 12 | 13 | 30 | 25 | 11 | 11 | 12 | 16 | - | T | 0 | 0 | 0 | 0 | 1 | 0 | 0 | 0 | 0 | 0 | 0 | 0 | 0 | 0 | 0 | 0 | 0 | 1 |
| H510 | 12 | 13 | 30 | 25 | 11 | 10 | 12 | 14 | - | T | 0 | 0 | 0 | 0 | 1 | 0 | 0 | 0 | 0 | 0 | 0 | 0 | 0 | 0 | 0 | 0 | 0 | 1 |
| H511 | 12 | 13 | 30 | 25 | 11 | 10 | 12 | 16 | - | T | 0 | 0 | 0 | 0 | 2 | 0 | 0 | 0 | 0 | 0 | 0 | 0 | 0 | 0 | 0 | 0 | 0 | 2 |
| H512 | 12 | 13 | 29 | 24 | 12 | 12 | 12 | 14 | - | C | 0 | 0 | 0 | 0 | 1 | 0 | 0 | 0 | 0 | 0 | 0 | 0 | 0 | 0 | 0 | 0 | 0 | 1 |
| H513 | 12 | 13 | 31 | 23 | 11 | 12 | 12 | 14 | - | C | 0 | 0 | 0 | 0 | 1 | 0 | 0 | 0 | 0 | 0 | 0 | 0 | 0 | 0 | 0 | 0 | 0 | 1 |
| H514 | 14 | 12 | 28 | 21 | 11 | 13 | 12 | 14 | - | T | 0 | 0 | 0 | 0 | 1 | 0 | 0 | 0 | 0 | 0 | 0 | 0 | 0 | 0 | 0 | 0 | 0 | 1 |
| H515 | 12 | 13 | 29 | 24 | 12 | 12 | 12 | 14 | - | T | 0 | 0 | 0 | 0 | 2 | 0 | 0 | 0 | 0 | 0 | 0 | 0 | 0 | 0 | 0 | 0 | 0 | 2 |
| H516 | 14 | 12 | 29 | 22 | 11 | 10 | 12 | 14 | - | C | 0 | 0 | 0 | 0 | 1 | 0 | 0 | 0 | 0 | 0 | 0 | 0 | 0 | 0 | 0 | 0 | 0 | 1 |
| H517 | 12 | 12 | 28 | 24 | 12 | 12 | 13 | 14 | - | C | 0 | 0 | 0 | 0 | 1 | 0 | 0 | 0 | 0 | 0 | 0 | 0 | 0 | 0 | 0 | 0 | 0 | 1 |
| H518 | 12 | 13 | 30 | 23 | 11 | 11 | 11 | 14 | - | C | 0 | 0 | 0 | 0 | 1 | 0 | 0 | 0 | 0 | 0 | 0 | 0 | 0 | 0 | 0 | 0 | 0 | 1 |
| H519 | 12 | 13 | 29 | 23 | 11 | 12 | 12 | 14 | - | T | 0 | 0 | 0 | 0 | 2 | 0 | 0 | 0 | 0 | 0 | 0 | 0 | 0 | 0 | 0 | 0 | 0 | 2 |
| H520 | 12 | 13 | 30 | 24 | 12 | 10 | 12 | 16 | - | T | 0 | 0 | 0 | 0 | 1 | 0 | 0 | 0 | 0 | 0 | 0 | 0 | 0 | 0 | 0 | 0 | 0 | 1 |
| H521 | 14 | 14 | 30 | 23 | 12 | 12 | 12 | 16 | - | T | 0 | 0 | 0 | 0 | 1 | 0 | 0 | 0 | 0 | 0 | 0 | 0 | 0 | 0 | 0 | 0 | 0 | 1 |
| H522 | 12 | 12 | 28 | 22 | 11 | 10 | 12 | 14 | - | T | 0 | 0 | 0 | 0 | 1 | 0 | 0 | 0 | 0 | 0 | 0 | 0 | 0 | 0 | 0 | 0 | 0 | 1 |
| H523 | 12 | 11 | 27 | 24 | 11 | 12 | 12 | 14 | - | T | 0 | 0 | 0 | 0 | 1 | 0 | 0 | 0 | 0 | 0 | 0 | 0 | 0 | 0 | 0 | 0 | 0 | 1 |
| H524 | 12 | 13 | 31 | 24 | 11 | 10 | 12 | 15 | - | C | 0 | 0 | 0 | 0 | 1 | 0 | 0 | 0 | 0 | 0 | 0 | 0 | 0 | 0 | 0 | 0 | 0 | 1 |
| H525 | 10 | 14 | 31 | 25 | 11 | 10 | 12 | 16 | - | T | 0 | 0 | 0 | 0 | 1 | 0 | 0 | 0 | 0 | 0 | 0 | 0 | 0 | 0 | 0 | 0 | 0 | 1 |
| H526 | 12 | 13 | 29 | 25 | 11 | 12 | 12 | 14 | - | T | 0 | 0 | 0 | 0 | 1 | 0 | 0 | 0 | 0 | 0 | 0 | 0 | 0 | 0 | 0 | 0 | 0 | 1 |
| H527 | 12 | 14 | 30 | 25 | 12 | 12 | 12 | 14 | - | C | 0 | 0 | 0 | 0 | 1 | 0 | 0 | 0 | 0 | 0 | 0 | 0 | 0 | 0 | 0 | 0 | 0 | 1 |

| | | | | | | | | | | | | | | | | | | | | | | | | | | | | |
|---|---|---|---|---|---|---|---|---|---|---|---|---|---|---|---|---|---|---|---|---|---|---|---|---|---|---|---|---|
| H528 | 12 | 13 | 29 | 25 | 12 | 12 | 12 | 15 | - | T | 0 | 0 | 0 | 0 | 1 | 0 | 0 | 0 | 0 | 0 | 0 | 0 | 0 | 0 | 0 | 0 | 0 | 1 |
| H529 | 15 | 12 | 28 | 24 | 11 | 11 | 11 | 15 | - | C | 0 | 0 | 0 | 0 | 1 | 0 | 0 | 0 | 0 | 0 | 0 | 0 | 0 | 0 | 0 | 0 | 0 | 1 |
| H530 | 12 | 13 | 29 | 26 | 12 | 13 | 12 | 14 | - | T | 0 | 0 | 0 | 0 | 1 | 0 | 0 | 0 | 0 | 0 | 0 | 0 | 0 | 0 | 0 | 0 | 0 | 1 |
| H531 | 12 | 13 | 29 | 25 | 11 | 13 | 12 | 14 | - | T | 0 | 0 | 0 | 0 | 1 | 0 | 0 | 0 | 0 | 0 | 0 | 0 | 0 | 0 | 0 | 0 | 0 | 1 |
| H532 | 12 | 13 | 30 | 24 | 11 | 10 | 12 | 13 | + | C | 0 | 0 | 0 | 0 | 1 | 0 | 0 | 0 | 0 | 0 | 0 | 0 | 0 | 1 | 0 | 0 | 0 | 2 |
| H533 | 12 | 13 | 30 | 25 | 10 | 10 | 13 | 13 | - | T | 0 | 0 | 0 | 0 | 1 | 0 | 0 | 0 | 0 | 0 | 0 | 0 | 0 | 0 | 0 | 0 | 0 | 1 |
| H534 | 12 | 13 | 29 | 25 | 12 | 13 | 12 | 14 | - | T | 0 | 0 | 0 | 0 | 2 | 0 | 0 | 0 | 0 | 0 | 0 | 0 | 0 | 0 | 0 | 0 | 0 | 2 |
| H535 | 13 | 13 | 31 | 22 | 12 | 12 | 13 | 15 | - | C | 0 | 0 | 0 | 0 | 1 | 0 | 0 | 0 | 0 | 0 | 0 | 0 | 0 | 0 | 0 | 0 | 0 | 1 |
| H536 | 12 | 13 | 30 | 24 | 11 | 11 | 12 | 13 | - | T | 0 | 0 | 0 | 0 | 1 | 0 | 0 | 0 | 0 | 0 | 0 | 0 | 0 | 0 | 0 | 0 | 0 | 1 |
| H537 | 12 | 13 | 29 | 23 | 11 | 13 | 12 | 14 | - | T | 0 | 0 | 0 | 0 | 2 | 0 | 0 | 0 | 0 | 0 | 0 | 0 | 0 | 0 | 0 | 0 | 0 | 2 |
| H538 | 12 | 12 | 28 | 22 | 11 | 13 | 13 | 15 | - | T | 0 | 0 | 0 | 0 | 1 | 0 | 0 | 0 | 0 | 0 | 0 | 0 | 0 | 0 | 0 | 0 | 0 | 1 |
| H539 | 12 | 13 | 30 | 25 | 12 | 13 | 11 | 14 | - | C | 0 | 0 | 0 | 0 | 1 | 0 | 0 | 0 | 0 | 0 | 0 | 0 | 0 | 0 | 0 | 0 | 0 | 1 |
| H540 | 12 | 13 | 29 | 24 | 12 | 13 | 12 | 14 | - | T | 0 | 0 | 0 | 0 | 1 | 0 | 0 | 0 | 0 | 0 | 0 | 0 | 0 | 0 | 0 | 0 | 0 | 1 |
| H541 | 12 | 14 | 30 | 23 | 12 | 14 | 12 | 14 | - | T | 0 | 0 | 0 | 0 | 1 | 0 | 0 | 0 | 0 | 0 | 0 | 0 | 0 | 0 | 0 | 0 | 0 | 1 |
| H542 | 12 | 13 | 30 | 26 | 11 | 11 | 12 | 16 | - | T | 0 | 0 | 0 | 0 | 1 | 0 | 0 | 0 | 0 | 0 | 0 | 0 | 0 | 0 | 0 | 0 | 0 | 1 |
| H543 | 12 | 13 | 29 | 24 | 12 | 14 | 12 | 14 | - | C | 0 | 0 | 0 | 0 | 1 | 0 | 0 | 0 | 0 | 0 | 0 | 0 | 0 | 0 | 0 | 0 | 0 | 1 |
| H544 | 12 | 13 | 31 | 24 | 11 | 12 | 12 | 13 | - | T | 0 | 0 | 0 | 0 | 1 | 0 | 0 | 0 | 0 | 0 | 0 | 0 | 0 | 0 | 0 | 0 | 0 | 1 |
| H545 | 12 | 13 | 29 | 23 | 11 | 13 | 12 | 16 | - | T | 0 | 0 | 0 | 0 | 1 | 0 | 0 | 0 | 0 | 0 | 0 | 0 | 0 | 0 | 0 | 0 | 0 | 1 |
| H546 | 12 | 14 | 30 | 24 | 12 | 13 | 12 | 15 | - | T | 0 | 0 | 0 | 0 | 1 | 0 | 0 | 0 | 0 | 0 | 0 | 0 | 0 | 0 | 0 | 0 | 0 | 1 |
| H547 | 12 | 13 | 31 | 25 | 10 | 11 | 13 | 13 | - | T | 0 | 0 | 0 | 0 | 1 | 0 | 0 | 0 | 0 | 0 | 0 | 0 | 0 | 0 | 0 | 0 | 0 | 1 |
| H548 | 12 | 13 | 31 | 23 | 11 | 11 | 11 | 14 | - | T | 0 | 0 | 0 | 0 | 2 | 0 | 0 | 0 | 0 | 0 | 0 | 0 | 0 | 0 | 0 | 0 | 0 | 2 |
| H549 | 12 | 12 | 28 | 23 | 12 | 13 | 12 | 14 | - | T | 0 | 0 | 0 | 0 | 1 | 0 | 0 | 0 | 0 | 0 | 0 | 0 | 0 | 0 | 0 | 0 | 0 | 1 |
| H550 | 12 | 13 | 30 | 25 | 11 | 13 | 12 | 14 | - | C | 0 | 0 | 0 | 0 | 1 | 0 | 0 | 0 | 0 | 0 | 0 | 0 | 0 | 0 | 0 | 0 | 0 | 1 |
| H551 | 12 | 12 | 28 | 25 | 13 | 13 | 12 | 14 | - | T | 0 | 0 | 0 | 0 | 1 | 0 | 0 | 0 | 0 | 0 | 0 | 0 | 0 | 0 | 0 | 0 | 0 | 1 |
| H552 | 15 | 12 | 28 | 22 | 11 | 11 | 12 | 14 | - | T | 0 | 0 | 0 | 0 | 1 | 0 | 0 | 0 | 0 | 0 | 0 | 0 | 0 | 0 | 0 | 0 | 0 | 1 |
| H553 | 12 | 14 | 31 | 23 | 11 | 11 | 12 | 15 | - | T | 0 | 0 | 0 | 0 | 1 | 0 | 0 | 0 | 0 | 0 | 0 | 0 | 0 | 0 | 0 | 0 | 0 | 1 |
| H554 | 12 | 13 | 29 | 23 | 12 | 13 | 12 | 14 | - | T | 0 | 0 | 0 | 0 | 1 | 0 | 0 | 0 | 0 | 0 | 0 | 0 | 0 | 0 | 0 | 0 | 0 | 1 |
| H555 | 12 | 14 | 31 | 23 | 11 | 12 | 11 | 14 | - | T | 0 | 0 | 0 | 0 | 1 | 0 | 0 | 0 | 0 | 0 | 0 | 0 | 0 | 0 | 0 | 0 | 0 | 1 |
| H556 | 12 | 13 | 29 | 24 | 12 | 13 | 12 | 15 | - | T | 0 | 0 | 0 | 0 | 1 | 0 | 0 | 0 | 0 | 0 | 0 | 0 | 0 | 0 | 0 | 0 | 0 | 1 |
| H557 | 12 | 14 | 31 | 24 | 11 | 13 | 12 | 15 | - | C | 0 | 0 | 0 | 0 | 1 | 0 | 0 | 0 | 0 | 0 | 0 | 0 | 0 | 0 | 0 | 0 | 0 | 1 |
| H558 | 12 | 13 | 30 | 24 | 11 | 11 | 11 | 14 | - | T | 0 | 0 | 0 | 0 | 1 | 0 | 0 | 0 | 0 | 0 | 0 | 0 | 0 | 0 | 0 | 0 | 0 | 1 |
| H559 | 13 | 13 | 29 | 23 | 12 | 13 | 12 | 14 | - | C | 0 | 0 | 0 | 0 | 1 | 0 | 0 | 0 | 0 | 0 | 0 | 0 | 0 | 0 | 0 | 0 | 0 | 1 |
| H560 | 13 | 14 | 31 | 24 | 12 | 12 | 12 | 13 | - | T | 0 | 0 | 0 | 0 | 1 | 0 | 0 | 0 | 0 | 0 | 0 | 0 | 0 | 0 | 0 | 0 | 0 | 1 |
| H561 | 12 | 14 | 30 | 23 | 12 | 13 | 12 | 14 | - | T | 0 | 0 | 0 | 0 | 1 | 0 | 0 | 0 | 0 | 0 | 0 | 0 | 0 | 0 | 0 | 0 | 0 | 1 |
| H562 | 15 | 14 | 30 | 25 | 11 | 11 | 11 | 14 | - | T | 0 | 0 | 0 | 0 | 1 | 0 | 0 | 0 | 0 | 0 | 0 | 0 | 0 | 0 | 0 | 0 | 0 | 1 |
| H563 | 12 | 13 | 30 | 24 | 12 | 12 | 12 | 16 | - | T | 0 | 0 | 0 | 0 | 1 | 0 | 0 | 0 | 0 | 0 | 0 | 0 | 0 | 0 | 0 | 0 | 0 | 1 |
| H564 | 14 | 12 | 28 | 22 | 11 | 11 | 12 | 15 | - | T | 0 | 0 | 0 | 0 | 1 | 0 | 0 | 0 | 0 | 0 | 0 | 0 | 0 | 0 | 0 | 0 | 0 | 1 |
| H565 | 13 | 14 | 29 | 23 | 11 | 13 | 13 | 17 | - | T | 0 | 0 | 0 | 0 | 1 | 0 | 0 | 0 | 0 | 0 | 0 | 0 | 0 | 0 | 0 | 0 | 0 | 1 |
| H566 | 12 | 13 | 30 | 21 | 11 | 14 | 12 | 14 | - | C | 0 | 0 | 0 | 0 | 1 | 0 | 0 | 0 | 0 | 0 | 0 | 0 | 0 | 0 | 0 | 0 | 0 | 1 |
| H567 | 12 | 13 | 29 | 25 | 11 | 11 | 12 | 15 | - | T | 0 | 0 | 0 | 0 | 1 | 0 | 0 | 0 | 0 | 0 | 0 | 0 | 0 | 0 | 0 | 0 | 0 | 1 |
| H568 | 15 | 13 | 30 | 23 | 11 | 11 | 11 | 14 | - | T | 0 | 0 | 0 | 0 | 1 | 0 | 0 | 0 | 0 | 0 | 0 | 0 | 0 | 0 | 0 | 0 | 0 | 1 |
| H569 | 13 | 13 | 29 | 23 | 11 | 12 | 13 | 16 | - | T | 0 | 0 | 0 | 0 | 1 | 0 | 0 | 0 | 0 | 0 | 0 | 0 | 0 | 0 | 0 | 0 | 0 | 1 |
| H570 | 12 | 13 | 29 | 24 | 12 | 13 | 13 | 14 | - | T | 0 | 0 | 0 | 0 | 1 | 0 | 0 | 0 | 0 | 0 | 0 | 0 | 0 | 0 | 0 | 0 | 0 | 1 |
| H571 | 13 | 14 | 29 | 22 | 12 | 12 | 13 | 16 | - | C | 0 | 0 | 0 | 0 | 0 | 1 | 0 | 0 | 0 | 0 | 0 | 0 | 0 | 0 | 0 | 0 | 0 | 1 |
| H572 | 12 | 13 | 30 | 21 | 11 | 13 | 14 | 16 | + | C | 0 | 0 | 0 | 0 | 0 | 2 | 0 | 0 | 0 | 0 | 0 | 0 | 0 | 0 | 0 | 0 | 0 | 2 |
| H573 | 12 | 13 | 31 | 21 | 11 | 12 | 12 | 16 | + | C | 0 | 0 | 0 | 0 | 0 | 3 | 0 | 0 | 0 | 0 | 0 | 0 | 0 | 0 | 0 | 0 | 2 | 5 |
| H574 | 12 | 13 | 31 | 21 | 11 | 12 | 11 | 15 | + | C | 0 | 0 | 0 | 0 | 0 | 2 | 0 | 0 | 0 | 0 | 0 | 0 | 0 | 0 | 0 | 0 | 1 | 3 |
| H575 | 12 | 13 | 30 | 21 | 10 | 11 | 13 | 15 | + | C | 0 | 0 | 0 | 0 | 0 | 1 | 0 | 1 | 0 | 0 | 0 | 0 | 0 | 1 | 0 | 0 | 0 | 3 |
| H576 | 12 | 14 | 31 | 21 | 11 | 12 | 13 | 17 | - | C | 0 | 0 | 0 | 0 | 0 | 1 | 0 | 0 | 0 | 0 | 0 | 0 | 0 | 0 | 0 | 0 | 0 | 1 |
| H577 | 12 | 14 | 31 | 21 | 11 | 11 | 12 | 17 | - | C | 0 | 0 | 0 | 0 | 0 | 1 | 0 | 0 | 0 | 0 | 0 | 0 | 0 | 0 | 0 | 0 | 0 | 1 |
| H578 | 12 | 13 | 31 | 21 | 12 | 11 | 14 | 16 | - | C | 0 | 0 | 0 | 0 | 0 | 1 | 0 | 0 | 0 | 0 | 0 | 0 | 0 | 0 | 0 | 0 | 0 | 1 |
| H579 | 12 | 13 | 31 | 21 | 11 | 11 | 12 | 14 | - | C | 0 | 0 | 0 | 0 | 0 | 1 | 0 | 0 | 0 | 0 | 0 | 0 | 0 | 0 | 0 | 0 | 0 | 1 |
| H580 | 12 | 13 | 30 | 20 | 11 | 11 | 14 | 14 | - | C | 0 | 0 | 0 | 0 | 0 | 1 | 0 | 0 | 0 | 0 | 0 | 0 | 0 | 0 | 0 | 0 | 0 | 1 |
| H581 | 12 | 14 | 31 | 21 | 11 | 11 | 14 | 15 | - | C | 0 | 0 | 0 | 0 | 0 | 1 | 0 | 0 | 0 | 0 | 0 | 0 | 0 | 0 | 0 | 0 | 0 | 1 |
| H582 | 12 | 13 | 31 | 22 | 11 | 12 | 14 | 16 | + | C | 0 | 0 | 0 | 0 | 0 | 1 | 0 | 0 | 0 | 0 | 0 | 0 | 0 | 0 | 0 | 0 | 0 | 1 |
| H583 | 12 | 14 | 31 | 21 | 11 | 12 | 12 | 17 | + | C | 0 | 0 | 0 | 0 | 0 | 1 | 0 | 0 | 0 | 0 | 0 | 0 | 0 | 0 | 0 | 0 | 0 | 1 |
| H584 | 12 | 13 | 31 | 21 | 12 | 12 | 14 | 17 | + | C | 0 | 0 | 0 | 0 | 0 | 1 | 0 | 0 | 0 | 0 | 0 | 0 | 0 | 0 | 0 | 0 | 0 | 1 |
| H585 | 12 | 13 | 29 | 21 | 11 | 12 | 12 | 17 | + | C | 0 | 0 | 0 | 0 | 0 | 1 | 0 | 0 | 0 | 0 | 0 | 0 | 0 | 0 | 0 | 0 | 0 | 1 |
| H586 | 12 | 13 | 30 | 21 | 11 | 11 | 15 | 17 | + | C | 0 | 0 | 0 | 0 | 0 | 1 | 0 | 0 | 0 | 0 | 0 | 0 | 0 | 0 | 0 | 0 | 0 | 1 |

| | | | | | | | | | | | | | | | | | | | | | | | | | | | | |
|---|---|---|---|---|---|---|---|---|---|---|---|---|---|---|---|---|---|---|---|---|---|---|---|---|---|---|---|---|
| H587 | 13 | 14 | 31 | 22 | 11 | 12 | 12 | 16 | + | C | 0 | 0 | 0 | 0 | 0 | 1 | 0 | 0 | 0 | 0 | 0 | 0 | 1 | 0 | 0 | 0 | 0 | 2 |
| H588 | 12 | 14 | 31 | 21 | 11 | 12 | 16 | 15 | + | C | 0 | 0 | 0 | 0 | 0 | 1 | 0 | 0 | 0 | 0 | 0 | 0 | 0 | 0 | 0 | 0 | 0 | 1 |
| H589 | 12 | 14 | 31 | 21 | 11 | 11 | 12 | 16 | + | C | 0 | 0 | 0 | 0 | 0 | 1 | 0 | 0 | 0 | 0 | 0 | 0 | 0 | 0 | 0 | 0 | 0 | 1 |
| H590 | 12 | 13 | 30 | 21 | 11 | 12 | 12 | 14 | + | C | 0 | 0 | 0 | 0 | 0 | 1 | 0 | 0 | 0 | 0 | 0 | 0 | 0 | 0 | 0 | 0 | 0 | 1 |
| H591 | 12 | 13 | 30 | 21 | 10 | 11 | 13 | 16 | + | C | 0 | 0 | 0 | 0 | 0 | 1 | 0 | 0 | 0 | 0 | 0 | 0 | 0 | 0 | 0 | 0 | 0 | 1 |
| H592 | 12 | 13 | 30 | 21 | 11 | 11 | 15 | 15 | + | C | 0 | 0 | 0 | 0 | 0 | 1 | 0 | 0 | 0 | 0 | 0 | 0 | 0 | 0 | 0 | 0 | 0 | 1 |
| H593 | 12 | 13 | 30 | 21 | 11 | 11 | 14 | 15 | + | C | 0 | 0 | 0 | 0 | 0 | 1 | 0 | 0 | 0 | 0 | 0 | 0 | 0 | 0 | 0 | 0 | 0 | 1 |
| H594 | 12 | 14 | 32 | 21 | 12 | 11 | 12 | 15 | + | C | 0 | 0 | 0 | 0 | 0 | 1 | 0 | 0 | 0 | 0 | 0 | 0 | 0 | 0 | 0 | 0 | 0 | 1 |
| H595 | 12 | 13 | 30 | 21 | 11 | 11 | 12 | 16 | + | C | 0 | 0 | 0 | 0 | 0 | 2 | 0 | 0 | 0 | 0 | 0 | 0 | 0 | 0 | 0 | 0 | 0 | 2 |
| H596 | 12 | 13 | 31 | 21 | 11 | 11 | 12 | 14 | + | C | 0 | 0 | 0 | 0 | 0 | 1 | 0 | 0 | 0 | 0 | 0 | 0 | 0 | 1 | 0 | 0 | 1 | 3 |
| H597 | 12 | 13 | 31 | 21 | 10 | 11 | 14 | 17 | + | C | 0 | 0 | 0 | 0 | 0 | 1 | 0 | 0 | 0 | 0 | 0 | 0 | 0 | 0 | 0 | 0 | 0 | 1 |
| H598 | 12 | 14 | 31 | 21 | 11 | 11 | 12 | 17 | + | C | 0 | 0 | 0 | 0 | 0 | 1 | 0 | 0 | 0 | 0 | 0 | 0 | 0 | 0 | 0 | 0 | 0 | 1 |
| H599 | 10 | 13 | 29 | 24 | 11 | 11 | 12 | 16 | - | C | 0 | 0 | 0 | 0 | 0 | 1 | 0 | 0 | 0 | 0 | 0 | 0 | 0 | 0 | 0 | 0 | 0 | 1 |
| H600 | 12 | 13 | 30 | 21 | 12 | 11 | 12 | 16 | + | C | 0 | 0 | 0 | 0 | 0 | 1 | 0 | 0 | 0 | 0 | 0 | 0 | 0 | 0 | 0 | 0 | 0 | 1 |
| H601 | 12 | 13 | 31 | 21 | 10 | 11 | 12 | 16 | + | C | 0 | 0 | 0 | 0 | 0 | 1 | 0 | 0 | 0 | 0 | 0 | 0 | 0 | 0 | 0 | 0 | 0 | 1 |
| H602 | 12 | 13 | 30 | 21 | 11 | 11 | 14 | 16 | + | C | 0 | 0 | 0 | 0 | 0 | 1 | 0 | 0 | 0 | 0 | 0 | 0 | 0 | 0 | 0 | 0 | 0 | 1 |
| H603 | 12 | 13 | 31 | 21 | 10 | 11 | 12 | 15 | + | C | 0 | 0 | 0 | 0 | 0 | 1 | 0 | 0 | 0 | 0 | 0 | 0 | 0 | 0 | 0 | 0 | 0 | 1 |
| H604 | 12 | 14 | 30 | 24 | 11 | 14 | 12 | 15 | - | T | 0 | 0 | 0 | 0 | 0 | 0 | 1 | 0 | 0 | 0 | 0 | 0 | 0 | 0 | 0 | 0 | 0 | 1 |
| H605 | 12 | 14 | 31 | 21 | 12 | 12 | 12 | 15 | + | C | 0 | 0 | 0 | 0 | 0 | 0 | 1 | 0 | 0 | 0 | 0 | 0 | 0 | 0 | 0 | 0 | 0 | 1 |
| H606 | 12 | 14 | 31 | 25 | 12 | 14 | 12 | 15 | - | T | 0 | 0 | 0 | 0 | 0 | 0 | 2 | 0 | 0 | 0 | 0 | 0 | 0 | 0 | 0 | 0 | 0 | 2 |
| H607 | 12 | 14 | 30 | 21 | 11 | 12 | 14 | 17 | + | C | 0 | 0 | 0 | 0 | 0 | 0 | 1 | 0 | 0 | 0 | 0 | 0 | 0 | 0 | 0 | 0 | 0 | 1 |
| H608 | 12 | 13 | 30 | 21 | 12 | 12 | 14 | 15 | + | C | 0 | 0 | 0 | 0 | 0 | 0 | 1 | 0 | 0 | 0 | 0 | 0 | 0 | 0 | 0 | 0 | 0 | 1 |
| H609 | 12 | 14 | 31 | 24 | 12 | 14 | 13 | 15 | - | C | 0 | 0 | 0 | 0 | 0 | 0 | 1 | 0 | 0 | 0 | 0 | 0 | 0 | 0 | 0 | 0 | 0 | 1 |
| H610 | 12 | 14 | 33 | 21 | 12 | 12 | 14 | 17 | - | T | 0 | 0 | 0 | 0 | 0 | 0 | 1 | 0 | 0 | 0 | 0 | 0 | 0 | 0 | 0 | 0 | 0 | 1 |
| H611 | 12 | 13 | 30 | 21 | 11 | 12 | 14 | 16 | - | T | 0 | 0 | 0 | 0 | 0 | 0 | 1 | 0 | 0 | 0 | 0 | 0 | 0 | 0 | 0 | 0 | 0 | 1 |
| H612 | 11 | 13 | 32 | 21 | 10 | 14 | 12 | 15 | - | C | 0 | 0 | 0 | 0 | 0 | 0 | 1 | 0 | 0 | 0 | 0 | 0 | 0 | 0 | 0 | 0 | 0 | 1 |
| H613 | 12 | 14 | 30 | 24 | 11 | 13 | 13 | 16 | - | T | 0 | 0 | 0 | 0 | 0 | 0 | 1 | 0 | 0 | 0 | 0 | 0 | 0 | 0 | 0 | 0 | 0 | 1 |
| H614 | 12 | 13 | 30 | 21 | 11 | 11 | 14 | 17 | + | C | 0 | 0 | 0 | 0 | 0 | 0 | 0 | 2 | 0 | 0 | 0 | 0 | 0 | 0 | 0 | 1 | 0 | 3 |
| H615 | 12 | 13 | 33 | 21 | 11 | 13 | 12 | 15 | + | C | 0 | 0 | 0 | 0 | 0 | 0 | 0 | 2 | 0 | 0 | 0 | 0 | 0 | 0 | 0 | 0 | 0 | 2 |
| H616 | 12 | 13 | 31 | 21 | 10 | 12 | 14 | 16 | + | C | 0 | 0 | 0 | 0 | 0 | 0 | 0 | 2 | 0 | 0 | 0 | 0 | 0 | 0 | 0 | 0 | 0 | 2 |
| H617 | 12 | 13 | 30 | 21 | 11 | 13 | 14 | 15 | + | C | 0 | 0 | 0 | 0 | 0 | 0 | 0 | 1 | 0 | 0 | 0 | 0 | 1 | 0 | 0 | 0 | 0 | 2 |
| H618 | 12 | 13 | 30 | 21 | 11 | 12 | 12 | 16 | + | C | 0 | 0 | 0 | 0 | 0 | 0 | 0 | 1 | 0 | 0 | 0 | 0 | 0 | 0 | 0 | 0 | 0 | 1 |
| H619 | 12 | 12 | 31 | 21 | 11 | 11 | 14 | 17 | + | C | 0 | 0 | 0 | 0 | 0 | 0 | 0 | 1 | 0 | 0 | 0 | 0 | 0 | 0 | 0 | 0 | 0 | 1 |
| H620 | 12 | 13 | 31 | 21 | 11 | 12 | 13 | 17 | + | C | 0 | 0 | 0 | 0 | 0 | 0 | 0 | 1 | 0 | 0 | 1 | 0 | 0 | 0 | 0 | 0 | 0 | 2 |
| H621 | 12 | 13 | 33 | 21 | 11 | 12 | 12 | 16 | + | C | 0 | 0 | 0 | 0 | 0 | 0 | 0 | 2 | 0 | 0 | 0 | 0 | 0 | 0 | 0 | 0 | 0 | 2 |
| H622 | 13 | 13 | 30 | 21 | 11 | 12 | 13 | 17 | + | C | 0 | 0 | 0 | 0 | 0 | 0 | 0 | 1 | 0 | 0 | 0 | 0 | 0 | 0 | 0 | 0 | 0 | 1 |
| H623 | 12 | 12 | 29 | 21 | 11 | 12 | 14 | 17 | + | C | 0 | 0 | 0 | 0 | 0 | 0 | 0 | 1 | 0 | 0 | 0 | 0 | 0 | 0 | 0 | 0 | 0 | 1 |
| H624 | 11 | 13 | 30 | 21 | 11 | 12 | 14 | 16 | + | C | 0 | 0 | 0 | 0 | 0 | 0 | 0 | 1 | 0 | 0 | 0 | 0 | 0 | 0 | 0 | 0 | 0 | 1 |
| H625 | 12 | 13 | 31 | 22 | 11 | 12 | 12 | 15 | + | C | 0 | 0 | 0 | 0 | 0 | 0 | 0 | 1 | 0 | 0 | 0 | 0 | 0 | 0 | 0 | 0 | 0 | 1 |
| H626 | 12 | 12 | 30 | 21 | 11 | 12 | 12 | 15 | + | C | 0 | 0 | 0 | 0 | 0 | 0 | 0 | 1 | 0 | 0 | 0 | 0 | 0 | 0 | 0 | 0 | 0 | 1 |
| H627 | 13 | 13 | 31 | 21 | 11 | 12 | 13 | 16 | + | C | 0 | 0 | 0 | 0 | 0 | 0 | 0 | 1 | 0 | 0 | 0 | 0 | 0 | 0 | 0 | 0 | 0 | 1 |
| H628 | 12 | 13 | 30 | 21 | 10 | 12 | 14 | 15 | + | C | 0 | 0 | 0 | 0 | 0 | 0 | 0 | 1 | 0 | 0 | 0 | 0 | 0 | 0 | 0 | 0 | 0 | 1 |
| H629 | 12 | 12 | 30 | 21 | 11 | 11 | 14 | 17 | + | C | 0 | 0 | 0 | 0 | 0 | 0 | 0 | 1 | 0 | 0 | 0 | 0 | 0 | 0 | 0 | 0 | 0 | 1 |
| H630 | 12 | 13 | 30 | 21 | 10 | 11 | 14 | 17 | + | C | 0 | 0 | 0 | 0 | 0 | 0 | 0 | 1 | 0 | 0 | 0 | 0 | 0 | 0 | 0 | 0 | 0 | 1 |
| H631 | 12 | 12 | 29 | 21 | 11 | 12 | 13 | 17 | + | C | 0 | 0 | 0 | 0 | 0 | 0 | 0 | 1 | 0 | 0 | 0 | 0 | 0 | 0 | 0 | 0 | 0 | 1 |
| H632 | 12 | 14 | 31 | 21 | 11 | 12 | 11 | 16 | + | C | 0 | 0 | 0 | 0 | 0 | 0 | 0 | 1 | 0 | 0 | 0 | 0 | 0 | 0 | 0 | 0 | 0 | 1 |
| H633 | 12 | 14 | 31 | 22 | 12 | 12 | 14 | 16 | + | C | 0 | 0 | 0 | 0 | 0 | 0 | 0 | 0 | 1 | 0 | 0 | 0 | 0 | 0 | 0 | 0 | 0 | 1 |
| H634 | 12 | 15 | 33 | 21 | 11 | 12 | 14 | 17 | + | C | 0 | 0 | 0 | 0 | 0 | 0 | 0 | 0 | 1 | 0 | 0 | 0 | 0 | 0 | 0 | 0 | 0 | 1 |
| H635 | 12 | 13 | 31 | 22 | 11 | 12 | 15 | 17 | + | C | 0 | 0 | 0 | 0 | 0 | 0 | 0 | 0 | 1 | 0 | 0 | 0 | 0 | 0 | 0 | 0 | 0 | 1 |
| H636 | 12 | 14 | 31 | 21 | 10 | 12 | 15 | 15 | + | C | 0 | 0 | 0 | 0 | 0 | 0 | 0 | 0 | 1 | 0 | 0 | 0 | 0 | 0 | 0 | 0 | 0 | 1 |
| H637 | 12 | 15 | 33 | 21 | 11 | 12 | 14 | 16 | + | C | 0 | 0 | 0 | 0 | 0 | 0 | 0 | 0 | 1 | 0 | 0 | 0 | 0 | 0 | 0 | 0 | 0 | 1 |
| H638 | 12 | 14 | 31 | 23 | 11 | 11 | 12 | 15 | + | C | 0 | 0 | 0 | 0 | 0 | 0 | 0 | 0 | 1 | 0 | 0 | 0 | 0 | 0 | 0 | 0 | 0 | 1 |
| H639 | 12 | 13 | 29 | 21 | 11 | 11 | 13 | 14 | + | C | 0 | 0 | 0 | 0 | 0 | 0 | 0 | 0 | 1 | 0 | 0 | 0 | 0 | 0 | 0 | 0 | 0 | 1 |
| H640 | 12 | 15 | 33 | 21 | 12 | 12 | 14 | 17 | + | C | 0 | 0 | 0 | 0 | 0 | 0 | 0 | 0 | 1 | 0 | 0 | 0 | 0 | 0 | 0 | 0 | 0 | 1 |
| H641 | 12 | 14 | 32 | 22 | 11 | 12 | 13 | 17 | + | C | 0 | 0 | 0 | 0 | 0 | 0 | 0 | 0 | 2 | 0 | 0 | 0 | 0 | 0 | 0 | 0 | 0 | 2 |
| H642 | 12 | 13 | 30 | 23 | 12 | 12 | 13 | 15 | + | C | 0 | 0 | 0 | 0 | 0 | 0 | 0 | 0 | 1 | 0 | 0 | 0 | 0 | 0 | 0 | 0 | 0 | 1 |
| H643 | 12 | 14 | 31 | 21 | 11 | 10 | 13 | 16 | + | C | 0 | 0 | 0 | 0 | 0 | 0 | 0 | 0 | 1 | 0 | 0 | 0 | 0 | 1 | 0 | 0 | 0 | 2 |
| H644 | 10 | 13 | 31 | 24 | 11 | 12 | 14 | 16 | - | C | 0 | 0 | 0 | 0 | 0 | 0 | 0 | 0 | 1 | 0 | 0 | 0 | 0 | 0 | 0 | 0 | 0 | 1 |
| H645 | 12 | 14 | 32 | 22 | 11 | 12 | 14 | 17 | + | C | 0 | 0 | 0 | 0 | 0 | 0 | 0 | 0 | 1 | 0 | 0 | 0 | 0 | 0 | 0 | 0 | 0 | 1 |

| | | | | | | | | | | | | | | | | | | | | | | | | | | | | |
|---|---|---|---|---|---|---|---|---|---|---|---|---|---|---|---|---|---|---|---|---|---|---|---|---|---|---|---|---|
| H646 | 12 | 13 | 29 | 22 | 11 | 12 | 11 | 13 | - | T | 0 | 0 | 0 | 0 | 0 | 0 | 0 | 0 | 0 | 1 | 0 | 0 | 0 | 0 | 0 | 0 | 0 | 1 |
| H647 | 12 | 13 | 29 | 22 | 11 | 10 | 12 | 14 | + | C | 0 | 0 | 0 | 0 | 0 | 0 | 0 | 0 | 0 | 1 | 0 | 0 | 0 | 0 | 0 | 0 | 0 | 1 |
| H648 | 12 | 13 | 31 | 20 | 11 | 10 | 14 | 16 | + | C | 0 | 0 | 0 | 0 | 0 | 0 | 0 | 0 | 0 | 1 | 0 | 0 | 0 | 0 | 0 | 0 | 0 | 1 |
| H649 | 13 | 14 | 32 | 22 | 11 | 10 | 12 | 17 | + | C | 0 | 0 | 0 | 0 | 0 | 0 | 0 | 0 | 0 | 1 | 0 | 0 | 0 | 0 | 0 | 0 | 0 | 1 |
| H650 | 12 | 14 | 30 | 21 | 10 | 10 | 14 | 15 | + | C | 0 | 0 | 0 | 0 | 0 | 0 | 0 | 0 | 0 | 1 | 0 | 0 | 0 | 0 | 0 | 0 | 0 | 1 |
| H651 | 12 | 13 | 30 | 24 | 10 | 12 | 11 | 14 | - | T | 0 | 0 | 0 | 0 | 0 | 0 | 0 | 0 | 0 | 1 | 0 | 0 | 0 | 0 | 0 | 0 | 0 | 1 |
| H652 | 12 | 14 | 31 | 21 | 9 | 10 | 12 | 14 | + | C | 0 | 0 | 0 | 0 | 0 | 0 | 0 | 0 | 0 | 1 | 0 | 0 | 0 | 0 | 0 | 0 | 0 | 1 |
| H653 | 12 | 13 | 30 | 22 | 10 | 10 | 13 | 14 | + | C | 0 | 0 | 0 | 0 | 0 | 0 | 0 | 0 | 0 | 4 | 0 | 0 | 0 | 2 | 0 | 0 | 0 | 6 |
| H654 | 14 | 12 | 28 | 23 | 10 | 10 | 12 | 13 | - | T | 0 | 0 | 0 | 0 | 0 | 0 | 0 | 0 | 0 | 1 | 0 | 0 | 0 | 0 | 0 | 0 | 0 | 1 |
| H655 | 12 | 13 | 29 | 24 | 11 | 13 | 12 | 13 | - | T | 0 | 0 | 0 | 0 | 0 | 0 | 0 | 0 | 0 | 1 | 0 | 0 | 0 | 0 | 0 | 0 | 0 | 1 |
| H656 | 12 | 13 | 31 | 22 | 10 | 10 | 12 | 14 | + | C | 0 | 0 | 0 | 0 | 0 | 0 | 0 | 0 | 0 | 1 | 0 | 0 | 0 | 0 | 0 | 0 | 0 | 1 |
| H657 | 12 | 13 | 30 | 21 | 10 | 10 | 12 | 14 | + | C | 0 | 0 | 0 | 0 | 0 | 0 | 0 | 0 | 0 | 1 | 0 | 0 | 0 | 7 | 0 | 0 | 0 | 8 |
| H658 | 12 | 12 | 30 | 22 | 10 | 10 | 13 | 14 | + | C | 0 | 0 | 0 | 0 | 0 | 0 | 0 | 0 | 0 | 1 | 0 | 0 | 0 | 0 | 0 | 0 | 0 | 1 |
| H659 | 12 | 13 | 29 | 24 | 11 | 12 | 12 | 14 | - | T | 0 | 0 | 0 | 0 | 0 | 0 | 0 | 0 | 0 | 1 | 0 | 0 | 0 | 2 | 0 | 0 | 0 | 3 |
| H660 | 12 | 13 | 30 | 25 | 10 | 12 | 12 | 14 | - | T | 0 | 0 | 0 | 0 | 0 | 0 | 0 | 0 | 0 | 1 | 0 | 0 | 0 | 0 | 0 | 0 | 0 | 1 |
| H661 | 12 | 11 | 27 | 24 | 10 | 12 | 12 | 14 | - | T | 0 | 0 | 0 | 0 | 0 | 0 | 0 | 0 | 0 | 1 | 0 | 0 | 0 | 0 | 0 | 0 | 0 | 1 |
| H662 | 12 | 13 | 30 | 21 | 9 | 10 | 14 | 15 | + | C | 0 | 0 | 0 | 0 | 0 | 0 | 0 | 0 | 0 | 1 | 0 | 0 | 0 | 0 | 0 | 0 | 0 | 1 |
| H663 | 12 | 13 | 29 | 25 | 10 | 13 | 12 | 13 | - | T | 0 | 0 | 0 | 0 | 0 | 0 | 0 | 0 | 0 | 1 | 0 | 0 | 0 | 0 | 0 | 0 | 0 | 1 |
| H664 | 14 | 12 | 28 | 24 | 11 | 10 | 12 | 13 | - | T | 0 | 0 | 0 | 0 | 0 | 0 | 0 | 0 | 0 | 1 | 0 | 0 | 0 | 0 | 0 | 0 | 0 | 1 |
| H665 | 13 | 14 | 30 | 23 | 10 | 10 | 14 | 15 | - | C | 0 | 0 | 0 | 0 | 0 | 0 | 0 | 0 | 0 | 1 | 0 | 0 | 0 | 0 | 0 | 0 | 0 | 1 |
| H666 | 12 | 13 | 29 | 24 | 10 | 12 | 12 | 13 | - | T | 0 | 0 | 0 | 0 | 0 | 0 | 0 | 0 | 0 | 1 | 0 | 0 | 0 | 2 | 0 | 0 | 0 | 3 |
| H667 | 12 | 13 | 29 | 25 | 10 | 12 | 14 | 14 | - | T | 0 | 0 | 0 | 0 | 0 | 0 | 0 | 0 | 0 | 1 | 0 | 0 | 0 | 0 | 0 | 0 | 0 | 1 |
| H668 | 12 | 13 | 30 | 20 | 10 | 10 | 12 | 14 | + | C | 0 | 0 | 0 | 0 | 0 | 0 | 0 | 0 | 0 | 1 | 0 | 0 | 0 | 0 | 0 | 0 | 0 | 1 |
| H669 | 13 | 14 | 32 | 23 | 10 | 11 | 13 | 14 | - | T | 0 | 0 | 0 | 0 | 0 | 0 | 0 | 0 | 0 | 1 | 0 | 0 | 0 | 0 | 0 | 0 | 0 | 1 |
| H670 | 12 | 14 | 30 | 24 | 11 | 12 | 12 | 13 | - | T | 0 | 0 | 0 | 0 | 0 | 0 | 0 | 0 | 0 | 1 | 0 | 0 | 0 | 1 | 0 | 0 | 0 | 2 |
| H671 | 12 | 13 | 29 | 21 | 10 | 10 | 14 | 17 | + | C | 0 | 0 | 0 | 0 | 0 | 0 | 0 | 0 | 0 | 1 | 0 | 0 | 0 | 0 | 0 | 0 | 0 | 1 |
| H672 | 12 | 14 | 31 | 21 | 12 | 10 | 12 | 14 | + | C | 0 | 0 | 0 | 0 | 0 | 0 | 0 | 0 | 0 | 1 | 0 | 0 | 0 | 0 | 0 | 0 | 0 | 1 |
| H673 | 16 | 13 | 30 | 23 | 9 | 10 | 11 | 14 | - | T | 0 | 0 | 0 | 0 | 0 | 0 | 0 | 0 | 0 | 1 | 0 | 0 | 0 | 0 | 0 | 0 | 0 | 1 |
| H674 | 12 | 12 | 28 | 24 | 10 | 12 | 12 | 13 | - | T | 0 | 0 | 0 | 0 | 0 | 0 | 0 | 0 | 0 | 1 | 0 | 0 | 0 | 0 | 0 | 0 | 0 | 1 |
| H675 | 12 | 13 | 31 | 20 | 11 | 10 | 13 | 15 | + | C | 0 | 0 | 0 | 0 | 0 | 0 | 0 | 0 | 0 | 1 | 0 | 0 | 0 | 0 | 0 | 0 | 0 | 1 |
| H676 | 12 | 12 | 29 | 23 | 11 | 13 | 12 | 15 | - | T | 0 | 0 | 0 | 0 | 0 | 0 | 0 | 0 | 0 | 1 | 0 | 0 | 0 | 0 | 0 | 0 | 0 | 1 |
| H677 | 14 | 13 | 31 | 21 | 10 | 10 | 12 | 14 | + | C | 0 | 0 | 0 | 0 | 0 | 0 | 0 | 0 | 0 | 1 | 0 | 0 | 0 | 0 | 0 | 0 | 0 | 1 |
| H678 | 12 | 13 | 30 | 21 | 12 | 10 | 12 | 14 | + | C | 0 | 0 | 0 | 0 | 0 | 0 | 0 | 0 | 0 | 1 | 0 | 0 | 0 | 0 | 0 | 0 | 0 | 1 |
| H679 | 12 | 13 | 32 | 21 | 10 | 10 | 12 | 14 | + | C | 0 | 0 | 0 | 0 | 0 | 0 | 0 | 0 | 0 | 1 | 0 | 0 | 0 | 1 | 0 | 0 | 0 | 2 |
| H680 | 13 | 12 | 28 | 23 | 10 | 10 | 13 | 14 | - | C | 0 | 0 | 0 | 0 | 0 | 0 | 0 | 0 | 0 | 1 | 0 | 0 | 0 | 0 | 0 | 0 | 0 | 1 |
| H681 | 12 | 13 | 29 | 21 | 10 | 10 | 13 | 15 | + | C | 0 | 0 | 0 | 0 | 0 | 0 | 0 | 0 | 0 | 1 | 0 | 0 | 0 | 0 | 0 | 0 | 0 | 1 |
| H682 | 12 | 14 | 30 | 23 | 10 | 10 | 12 | 14 | - | T | 0 | 0 | 0 | 0 | 0 | 0 | 0 | 0 | 0 | 1 | 0 | 0 | 0 | 0 | 0 | 0 | 0 | 1 |
| H683 | 10 | 12 | 28 | 23 | 10 | 13 | 11 | 14 | - | T | 0 | 0 | 0 | 0 | 0 | 0 | 0 | 0 | 0 | 1 | 0 | 0 | 0 | 0 | 0 | 0 | 0 | 1 |
| H684 | 12 | 13 | 30 | 20 | 10 | 11 | 14 | 14 | + | C | 0 | 0 | 0 | 0 | 0 | 0 | 0 | 0 | 0 | 1 | 0 | 0 | 0 | 0 | 0 | 0 | 0 | 1 |
| H685 | 13 | 14 | 31 | 21 | 10 | 10 | 14 | 16 | + | C | 0 | 0 | 0 | 0 | 0 | 0 | 0 | 0 | 0 | 1 | 0 | 0 | 0 | 0 | 0 | 0 | 0 | 1 |
| H686 | 12 | 15 | 32 | 21 | 10 | 10 | 13 | 15 | + | C | 0 | 0 | 0 | 0 | 0 | 0 | 0 | 0 | 0 | 1 | 0 | 0 | 0 | 0 | 0 | 0 | 0 | 1 |
| H687 | 12 | 13 | 28 | 23 | 9 | 12 | 11 | 13 | - | T | 0 | 0 | 0 | 0 | 0 | 0 | 0 | 0 | 0 | 1 | 0 | 0 | 0 | 0 | 0 | 0 | 0 | 1 |
| H688 | 13 | 13 | 30 | 22 | 11 | 12 | 12 | 17 | + | C | 0 | 0 | 0 | 0 | 0 | 0 | 0 | 0 | 0 | 0 | 1 | 0 | 1 | 0 | 0 | 0 | 0 | 2 |
| H689 | 13 | 14 | 31 | 22 | 11 | 12 | 11 | 16 | + | C | 0 | 0 | 0 | 0 | 0 | 0 | 0 | 0 | 0 | 0 | 1 | 0 | 0 | 0 | 0 | 0 | 0 | 1 |
| H690 | 14 | 13 | 30 | 22 | 11 | 12 | 12 | 17 | + | C | 0 | 0 | 0 | 0 | 0 | 0 | 0 | 0 | 0 | 0 | 1 | 0 | 0 | 0 | 0 | 0 | 0 | 1 |
| H691 | 12 | 14 | 31 | 21 | 10 | 12 | 14 | 15 | + | C | 0 | 0 | 0 | 0 | 0 | 0 | 0 | 0 | 0 | 0 | 1 | 0 | 0 | 0 | 0 | 0 | 0 | 1 |
| H692 | 13 | 13 | 30 | 21 | 11 | 12 | 13 | 15 | + | C | 0 | 0 | 0 | 0 | 0 | 0 | 0 | 0 | 0 | 0 | 1 | 0 | 0 | 0 | 0 | 0 | 0 | 1 |
| H693 | 12 | 13 | 30 | 21 | 9 | 12 | 13 | 15 | + | C | 0 | 0 | 0 | 0 | 0 | 0 | 0 | 0 | 0 | 0 | 1 | 0 | 0 | 0 | 0 | 0 | 0 | 1 |
| H694 | 13 | 14 | 31 | 22 | 11 | 12 | 12 | 17 | + | C | 0 | 0 | 0 | 0 | 0 | 0 | 0 | 0 | 0 | 0 | 1 | 0 | 1 | 0 | 1 | 0 | 0 | 3 |
| H695 | 12 | 13 | 31 | 21 | 12 | 12 | 12 | 15 | + | C | 0 | 0 | 0 | 0 | 0 | 0 | 0 | 0 | 0 | 0 | 1 | 0 | 0 | 0 | 0 | 0 | 0 | 1 |
| H696 | 13 | 14 | 32 | 22 | 11 | 12 | 13 | 17 | + | C | 0 | 0 | 0 | 0 | 0 | 0 | 0 | 0 | 0 | 0 | 1 | 0 | 0 | 0 | 0 | 0 | 0 | 1 |
| H697 | 13 | 13 | 31 | 23 | 11 | 12 | 12 | 17 | + | C | 0 | 0 | 0 | 0 | 0 | 0 | 0 | 0 | 0 | 0 | 1 | 0 | 0 | 0 | 0 | 0 | 0 | 1 |
| H698 | 12 | 13 | 31 | 21 | 11 | 11 | 12 | 16 | + | C | 0 | 0 | 0 | 0 | 0 | 0 | 0 | 0 | 0 | 0 | 1 | 0 | 0 | 0 | 0 | 0 | 0 | 1 |
| H699 | 11 | 14 | 31 | 22 | 11 | 12 | 12 | 16 | + | C | 0 | 0 | 0 | 0 | 0 | 0 | 0 | 0 | 0 | 0 | 1 | 0 | 0 | 0 | 0 | 0 | 0 | 1 |
| H700 | 12 | 13 | 30 | 21 | 10 | 11 | 13 | 17 | + | C | 0 | 0 | 0 | 0 | 0 | 0 | 0 | 0 | 0 | 0 | 1 | 0 | 0 | 0 | 0 | 0 | 0 | 1 |
| H701 | 14 | 14 | 31 | 21 | 11 | 12 | 12 | 15 | + | C | 0 | 0 | 0 | 0 | 0 | 0 | 0 | 0 | 0 | 0 | 0 | 1 | 0 | 0 | 0 | 0 | 0 | 1 |
| H702 | 14 | 14 | 31 | 21 | 12 | 12 | 12 | 15 | - | C | 0 | 0 | 0 | 0 | 0 | 0 | 0 | 0 | 0 | 0 | 0 | 2 | 0 | 0 | 0 | 0 | 0 | 2 |
| H703 | 12 | 13 | 31 | 20 | 11 | 12 | 14 | 16 | + | C | 0 | 0 | 0 | 0 | 0 | 0 | 0 | 0 | 0 | 0 | 0 | 1 | 0 | 0 | 0 | 0 | 0 | 1 |
| H704 | 12 | 13 | 32 | 21 | 12 | 12 | 12 | 15 | + | C | 0 | 0 | 0 | 0 | 0 | 0 | 0 | 0 | 0 | 0 | 0 | 1 | 0 | 0 | 0 | 0 | 0 | 1 |

| | | | | | | | | | | | | | | | | | | | | | | | | | | | | |
|---|---|---|---|---|---|---|---|---|---|---|---|---|---|---|---|---|---|---|---|---|---|---|---|---|---|---|---|---|
| H705 | 12 | 13 | 31 | 20 | 11 | 12 | 14 | 11 | + | C | 0 | 0 | 0 | 0 | 0 | 0 | 0 | 0 | 0 | 0 | 0 | 1 | 0 | 0 | 0 | 0 | 0 | 1 |
| H706 | 14 | 13 | 30 | 21 | 11 | 12 | 12 | 15 | + | C | 0 | 0 | 0 | 0 | 0 | 0 | 0 | 0 | 0 | 0 | 0 | 1 | 0 | 0 | 0 | 0 | 0 | 1 |
| H707 | 9 | 12 | 29 | 22 | 13 | 12 | 13 | 14 | - | C | 0 | 0 | 0 | 0 | 0 | 0 | 0 | 0 | 0 | 0 | 0 | 1 | 0 | 0 | 0 | 0 | 0 | 1 |
| H708 | 12 | 13 | 30 | 21 | 11 | 12 | 13 | 11 | + | C | 0 | 0 | 0 | 0 | 0 | 0 | 0 | 0 | 0 | 0 | 0 | 1 | 0 | 0 | 0 | 0 | 0 | 1 |
| H709 | 12 | 13 | 31 | 21 | 11 | 12 | 13 | 14 | + | C | 0 | 0 | 0 | 0 | 0 | 0 | 0 | 0 | 0 | 0 | 0 | 1 | 0 | 0 | 0 | 0 | 0 | 1 |
| H710 | 14 | 14 | 31 | 21 | 12 | 12 | 12 | 15 | + | C | 0 | 0 | 0 | 0 | 0 | 0 | 0 | 0 | 0 | 0 | 0 | 1 | 0 | 0 | 0 | 0 | 0 | 1 |
| H711 | 14 | 13 | 30 | 21 | 11 | 12 | 13 | 16 | + | C | 0 | 0 | 0 | 0 | 0 | 0 | 0 | 0 | 0 | 0 | 0 | 1 | 0 | 0 | 0 | 0 | 0 | 1 |
| H712 | 12 | 14 | 31 | 21 | 12 | 12 | 12 | 16 | + | C | 0 | 0 | 0 | 0 | 0 | 0 | 0 | 0 | 0 | 0 | 0 | 1 | 0 | 0 | 0 | 0 | 0 | 1 |
| H713 | 13 | 15 | 32 | 22 | 11 | 12 | 13 | 17 | + | C | 0 | 0 | 0 | 0 | 0 | 0 | 0 | 0 | 0 | 0 | 0 | 0 | 2 | 0 | 0 | 0 | 0 | 2 |
| H714 | 12 | 13 | 32 | 21 | 10 | 12 | 13 | 16 | + | C | 0 | 0 | 0 | 0 | 0 | 0 | 0 | 0 | 0 | 0 | 0 | 0 | 1 | 0 | 0 | 0 | 0 | 1 |
| H715 | 12 | 15 | 32 | 21 | 11 | 12 | 13 | 13 | + | C | 0 | 0 | 0 | 0 | 0 | 0 | 0 | 0 | 0 | 0 | 0 | 0 | 1 | 0 | 0 | 0 | 0 | 1 |
| H716 | 12 | 14 | 32 | 21 | 11 | 12 | 15 | 15 | + | C | 0 | 0 | 0 | 0 | 0 | 0 | 0 | 0 | 0 | 0 | 0 | 0 | 1 | 0 | 0 | 1 | 0 | 2 |
| H717 | 12 | 13 | 29 | 23 | 11 | 12 | 13 | 15 | - | T | 0 | 0 | 0 | 0 | 0 | 0 | 0 | 0 | 0 | 0 | 0 | 0 | 1 | 0 | 0 | 0 | 0 | 1 |
| H718 | 13 | 15 | 33 | 22 | 11 | 12 | 14 | 17 | + | C | 0 | 0 | 0 | 0 | 0 | 0 | 0 | 0 | 0 | 0 | 0 | 0 | 1 | 0 | 0 | 0 | 0 | 1 |
| H719 | 12 | 14 | 30 | 24 | 10 | 12 | 13 | 13 | + | C | 0 | 0 | 0 | 0 | 0 | 0 | 0 | 0 | 0 | 0 | 0 | 0 | 1 | 0 | 0 | 0 | 0 | 1 |
| H720 | 13 | 14 | 31 | 22 | 11 | 12 | 13 | 16 | + | C | 0 | 0 | 0 | 0 | 0 | 0 | 0 | 0 | 0 | 0 | 0 | 0 | 1 | 0 | 0 | 0 | 0 | 1 |
| H721 | 13 | 15 | 32 | 23 | 11 | 12 | 13 | 16 | + | C | 0 | 0 | 0 | 0 | 0 | 0 | 0 | 0 | 0 | 0 | 0 | 0 | 1 | 0 | 0 | 0 | 0 | 1 |
| H722 | 12 | 14 | 31 | 21 | 11 | 12 | 13 | 14 | + | C | 0 | 0 | 0 | 0 | 0 | 0 | 0 | 0 | 0 | 0 | 0 | 0 | 1 | 0 | 0 | 0 | 0 | 1 |
| H723 | 12 | 14 | 33 | 20 | 11 | 12 | 14 | 16 | + | C | 0 | 0 | 0 | 0 | 0 | 0 | 0 | 0 | 0 | 0 | 0 | 0 | 1 | 0 | 0 | 0 | 0 | 1 |
| H724 | 13 | 13 | 31 | 22 | 11 | 12 | 13 | 15 | + | C | 0 | 0 | 0 | 0 | 0 | 0 | 0 | 0 | 0 | 0 | 0 | 0 | 1 | 0 | 0 | 0 | 0 | 1 |
| H725 | 12 | 14 | 30 | 21 | 11 | 12 | 15 | 15 | + | C | 0 | 0 | 0 | 0 | 0 | 0 | 0 | 0 | 0 | 0 | 0 | 0 | 1 | 0 | 0 | 0 | 0 | 1 |
| H726 | 13 | 13 | 30 | 22 | 12 | 12 | 12 | 17 | + | C | 0 | 0 | 0 | 0 | 0 | 0 | 0 | 0 | 0 | 0 | 0 | 0 | 1 | 0 | 0 | 0 | 0 | 1 |
| H727 | 12 | 12 | 30 | 21 | 12 | 12 | 13 | 15 | + | C | 0 | 0 | 0 | 0 | 0 | 0 | 0 | 0 | 0 | 0 | 0 | 0 | 1 | 0 | 0 | 0 | 0 | 1 |
| H728 | 11 | 15 | 31 | 22 | 12 | 12 | 12 | 16 | + | C | 0 | 0 | 0 | 0 | 0 | 0 | 0 | 0 | 0 | 0 | 0 | 0 | 1 | 0 | 0 | 0 | 0 | 1 |
| H729 | 12 | 13 | 30 | 21 | 10 | 12 | 13 | 15 | + | C | 0 | 0 | 0 | 0 | 0 | 0 | 0 | 0 | 0 | 0 | 0 | 0 | 1 | 0 | 0 | 0 | 0 | 1 |
| H730 | 11 | 13 | 31 | 21 | 11 | 12 | 12 | 15 | + | C | 0 | 0 | 0 | 0 | 0 | 0 | 0 | 0 | 0 | 0 | 0 | 0 | 1 | 0 | 0 | 0 | 0 | 1 |
| H731 | 11 | 14 | 32 | 21 | 11 | 11 | 13 | 14 | + | C | 0 | 0 | 0 | 0 | 0 | 0 | 0 | 0 | 0 | 0 | 0 | 0 | 1 | 0 | 0 | 0 | 0 | 1 |
| H732 | 12 | 14 | 31 | 20 | 11 | 12 | 13 | 15 | + | C | 0 | 0 | 0 | 0 | 0 | 0 | 0 | 0 | 0 | 0 | 0 | 0 | 1 | 0 | 0 | 0 | 0 | 1 |
| H733 | 13 | 14 | 31 | 22 | 11 | 12 | 12 | 15 | + | C | 0 | 0 | 0 | 0 | 0 | 0 | 0 | 0 | 0 | 0 | 0 | 0 | 1 | 0 | 0 | 0 | 0 | 1 |
| H734 | 13 | 15 | 33 | 22 | 11 | 12 | 12 | 17 | + | C | 0 | 0 | 0 | 0 | 0 | 0 | 0 | 0 | 0 | 0 | 0 | 0 | 1 | 0 | 0 | 0 | 0 | 1 |
| H735 | 11 | 14 | 32 | 21 | 11 | 13 | 12 | 15 | + | C | 0 | 0 | 0 | 0 | 0 | 0 | 0 | 0 | 0 | 0 | 0 | 0 | 1 | 0 | 0 | 0 | 0 | 1 |
| H736 | 12 | 12 | 29 | 21 | 11 | 13 | 13 | 14 | + | C | 0 | 0 | 0 | 0 | 0 | 0 | 0 | 0 | 0 | 0 | 0 | 0 | 1 | 0 | 0 | 0 | 0 | 1 |
| H737 | 13 | 15 | 32 | 22 | 12 | 12 | 12 | 17 | + | C | 0 | 0 | 0 | 0 | 0 | 0 | 0 | 0 | 0 | 0 | 0 | 0 | 1 | 0 | 0 | 0 | 0 | 1 |
| H738 | 13 | 15 | 32 | 22 | 11 | 12 | 11 | 15 | + | C | 0 | 0 | 0 | 0 | 0 | 0 | 0 | 0 | 0 | 0 | 0 | 0 | 1 | 0 | 0 | 0 | 0 | 1 |
| H739 | 13 | 14 | 30 | 23 | 10 | 11 | 13 | 14 | - | T | 0 | 0 | 0 | 0 | 0 | 0 | 0 | 0 | 0 | 0 | 0 | 0 | 0 | 1 | 0 | 0 | 0 | 1 |
| H740 | 14 | 12 | 28 | 22 | 11 | 10 | 12 | 13 | - | T | 0 | 0 | 0 | 0 | 0 | 0 | 0 | 0 | 0 | 0 | 0 | 0 | 0 | 1 | 0 | 0 | 0 | 1 |
| H741 | 12 | 13 | 30 | 20 | 10 | 10 | 13 | 14 | + | C | 0 | 0 | 0 | 0 | 0 | 0 | 0 | 0 | 0 | 0 | 0 | 0 | 0 | 1 | 0 | 0 | 0 | 1 |
| H742 | 12 | 14 | 31 | 21 | 10 | 10 | 15 | 16 | + | C | 0 | 0 | 0 | 0 | 0 | 0 | 0 | 0 | 0 | 0 | 0 | 0 | 0 | 1 | 0 | 0 | 0 | 1 |
| H743 | 12 | 12 | 29 | 21 | 10 | 10 | 14 | 15 | + | C | 0 | 0 | 0 | 0 | 0 | 0 | 0 | 0 | 0 | 0 | 0 | 0 | 0 | 1 | 0 | 0 | 0 | 1 |
| H744 | 12 | 14 | 31 | 21 | 10 | 10 | 12 | 15 | + | C | 0 | 0 | 0 | 0 | 0 | 0 | 0 | 0 | 0 | 0 | 0 | 0 | 0 | 1 | 0 | 0 | 0 | 1 |
| H745 | 10 | 13 | 31 | 24 | 10 | 10 | 12 | 15 | - | C | 0 | 0 | 0 | 0 | 0 | 0 | 0 | 0 | 0 | 0 | 0 | 0 | 0 | 1 | 0 | 0 | 0 | 1 |
| H746 | 12 | 12 | 28 | 24 | 10 | 10 | 13 | 13 | - | T | 0 | 0 | 0 | 0 | 0 | 0 | 0 | 0 | 0 | 0 | 0 | 0 | 0 | 1 | 0 | 0 | 0 | 1 |
| H747 | 12 | 13 | 30 | 21 | 10 | 10 | 11 | 14 | + | C | 0 | 0 | 0 | 0 | 0 | 0 | 0 | 0 | 0 | 0 | 0 | 0 | 0 | 1 | 0 | 0 | 0 | 1 |
| H748 | 12 | 14 | 31 | 21 | 11 | 10 | 13 | 15 | + | C | 0 | 0 | 0 | 0 | 0 | 0 | 0 | 0 | 0 | 0 | 0 | 0 | 0 | 1 | 0 | 0 | 0 | 1 |
| H749 | 12 | 14 | 31 | 22 | 11 | 10 | 11 | 13 | - | T | 0 | 0 | 0 | 0 | 0 | 0 | 0 | 0 | 0 | 0 | 0 | 0 | 0 | 1 | 0 | 0 | 0 | 1 |
| H750 | 13 | 13 | 29 | 24 | 10 | 10 | 12 | 13 | + | C | 0 | 0 | 0 | 0 | 0 | 0 | 0 | 0 | 0 | 0 | 0 | 0 | 0 | 1 | 0 | 0 | 0 | 1 |
| H751 | 12 | 14 | 31 | 21 | 10 | 10 | 12 | 16 | + | C | 0 | 0 | 0 | 0 | 0 | 0 | 0 | 0 | 0 | 0 | 0 | 0 | 0 | 1 | 0 | 0 | 0 | 1 |
| H752 | 14 | 12 | 29 | 23 | 10 | 10 | 12 | 13 | - | T | 0 | 0 | 0 | 0 | 0 | 0 | 0 | 0 | 0 | 0 | 0 | 0 | 0 | 1 | 0 | 0 | 0 | 1 |
| H753 | 12 | 13 | 31 | 23 | 9 | 10 | 12 | 10 | + | C | 0 | 0 | 0 | 0 | 0 | 0 | 0 | 0 | 0 | 0 | 0 | 0 | 0 | 1 | 0 | 0 | 0 | 1 |
| H754 | 15 | 12 | 28 | 24 | 10 | 10 | 11 | 14 | - | T | 0 | 0 | 0 | 0 | 0 | 0 | 0 | 0 | 0 | 0 | 0 | 0 | 0 | 1 | 0 | 0 | 0 | 1 |
| H755 | 12 | 13 | 29 | 24 | 10 | 12 | 12 | 14 | - | T | 0 | 0 | 0 | 0 | 0 | 0 | 0 | 0 | 0 | 0 | 0 | 0 | 0 | 1 | 0 | 0 | 0 | 1 |
| H756 | 12 | 12 | 30 | 21 | 10 | 9 | 12 | 14 | + | C | 0 | 0 | 0 | 0 | 0 | 0 | 0 | 0 | 0 | 0 | 0 | 0 | 0 | 1 | 0 | 0 | 0 | 1 |
| H757 | 12 | 13 | 29 | 21 | 11 | 10 | 13 | 16 | + | C | 0 | 0 | 0 | 0 | 0 | 0 | 0 | 0 | 0 | 0 | 0 | 0 | 0 | 1 | 0 | 0 | 0 | 1 |
| H758 | 16 | 13 | 29 | 25 | 10 | 12 | 11 | 13 | - | T | 0 | 0 | 0 | 0 | 0 | 0 | 0 | 0 | 0 | 0 | 0 | 0 | 0 | 1 | 0 | 0 | 0 | 1 |
| H759 | 12 | 13 | 29 | 23 | 10 | 11 | 12 | 13 | - | T | 0 | 0 | 0 | 0 | 0 | 0 | 0 | 0 | 0 | 0 | 0 | 0 | 0 | 1 | 0 | 0 | 0 | 1 |
| H760 | 12 | 13 | 28 | 23 | 10 | 13 | 11 | 13 | - | T | 0 | 0 | 0 | 0 | 0 | 0 | 0 | 0 | 0 | 0 | 0 | 0 | 0 | 1 | 0 | 0 | 0 | 1 |
| H761 | 12 | 13 | 29 | 26 | 11 | 12 | 12 | 13 | - | T | 0 | 0 | 0 | 0 | 0 | 0 | 0 | 0 | 0 | 0 | 0 | 0 | 0 | 1 | 0 | 0 | 0 | 1 |
| H762 | 16 | 13 | 30 | 23 | 10 | 10 | 11 | 14 | - | T | 0 | 0 | 0 | 0 | 0 | 0 | 0 | 0 | 0 | 0 | 0 | 0 | 0 | 1 | 0 | 0 | 0 | 1 |
| H763 | 12 | 13 | 30 | 21 | 9 | 10 | 12 | 14 | + | C | 0 | 0 | 0 | 0 | 0 | 0 | 0 | 0 | 0 | 0 | 0 | 0 | 0 | 1 | 0 | 0 | 0 | 1 |

| | | | | | | | | | | | | | | | | | | | | | | | | | | | | |
|---|---|---|---|---|---|---|---|---|---|---|---|---|---|---|---|---|---|---|---|---|---|---|---|---|---|---|---|---|
| H764 | 13 | 12 | 28 | 22 | 10 | 11 | 15 | 13 | - | T | 0 | 0 | 0 | 0 | 0 | 0 | 0 | 0 | 0 | 0 | 0 | 0 | 0 | 1 | 0 | 0 | 0 | 1 |
| H765 | 12 | 12 | 28 | 24 | 11 | 10 | 12 | 13 | + | C | 0 | 0 | 0 | 0 | 0 | 0 | 0 | 0 | 0 | 0 | 0 | 0 | 0 | 1 | 0 | 0 | 0 | 1 |
| H766 | 12 | 14 | 31 | 21 | 10 | 11 | 13 | 14 | + | C | 0 | 0 | 0 | 0 | 0 | 0 | 0 | 0 | 0 | 0 | 0 | 0 | 0 | 1 | 0 | 0 | 0 | 1 |
| H767 | 12 | 14 | 29 | 21 | 10 | 10 | 12 | 14 | + | C | 0 | 0 | 0 | 0 | 0 | 0 | 0 | 0 | 0 | 0 | 0 | 0 | 0 | 1 | 0 | 0 | 0 | 1 |
| H768 | 13 | 13 | 31 | 23 | 10 | 13 | 12 | 16 | - | T | 0 | 0 | 0 | 0 | 0 | 0 | 0 | 0 | 0 | 0 | 0 | 0 | 0 | 1 | 0 | 0 | 0 | 1 |
| H769 | 12 | 13 | 29 | 21 | 10 | 10 | 13 | 16 | + | C | 0 | 0 | 0 | 0 | 0 | 0 | 0 | 0 | 0 | 0 | 0 | 0 | 0 | 1 | 0 | 0 | 0 | 1 |
| H770 | 12 | 13 | 30 | 21 | 10 | 11 | 14 | 15 | + | C | 0 | 0 | 0 | 0 | 0 | 0 | 0 | 0 | 0 | 0 | 0 | 0 | 0 | 2 | 0 | 0 | 0 | 2 |
| H771 | 12 | 13 | 30 | 21 | 11 | 10 | 12 | 16 | + | C | 0 | 0 | 0 | 0 | 0 | 0 | 0 | 0 | 0 | 0 | 0 | 0 | 0 | 1 | 0 | 0 | 0 | 1 |
| H772 | 12 | 14 | 31 | 21 | 10 | 10 | 12 | 14 | + | C | 0 | 0 | 0 | 0 | 0 | 0 | 0 | 0 | 0 | 0 | 0 | 0 | 0 | 1 | 0 | 0 | 0 | 1 |
| H773 | 12 | 12 | 27 | 24 | 11 | 12 | 12 | 13 | - | T | 0 | 0 | 0 | 0 | 0 | 0 | 0 | 0 | 0 | 0 | 0 | 0 | 0 | 1 | 0 | 0 | 0 | 1 |
| H774 | 12 | 14 | 30 | 21 | 10 | 10 | 13 | 15 | - | T | 0 | 0 | 0 | 0 | 0 | 0 | 0 | 0 | 0 | 0 | 0 | 0 | 0 | 1 | 0 | 0 | 0 | 1 |
| H775 | 12 | 14 | 30 | 24 | 11 | 12 | 12 | 14 | - | T | 0 | 0 | 0 | 0 | 0 | 0 | 0 | 0 | 0 | 0 | 0 | 0 | 0 | 1 | 0 | 0 | 0 | 1 |
| H776 | 13 | 14 | 31 | 23 | 9 | 12 | 14 | 14 | - | T | 0 | 0 | 0 | 0 | 0 | 0 | 0 | 0 | 0 | 0 | 0 | 0 | 0 | 1 | 0 | 0 | 0 | 1 |
| H777 | 12 | 12 | 29 | 21 | 10 | 10 | 13 | 15 | + | C | 0 | 0 | 0 | 0 | 0 | 0 | 0 | 0 | 0 | 0 | 0 | 0 | 0 | 1 | 0 | 0 | 0 | 1 |
| H778 | 13 | 13 | 29 | 22 | 10 | 11 | 12 | 15 | - | T | 0 | 0 | 0 | 0 | 0 | 0 | 0 | 0 | 0 | 0 | 0 | 0 | 0 | 1 | 0 | 0 | 0 | 1 |
| H779 | 12 | 13 | 30 | 24 | 10 | 10 | 12 | 12 | + | C | 0 | 0 | 0 | 0 | 0 | 0 | 0 | 0 | 0 | 0 | 0 | 0 | 0 | 1 | 0 | 0 | 0 | 1 |
| H780 | 12 | 12 | 29 | 21 | 10 | 11 | 14 | 15 | + | C | 0 | 0 | 0 | 0 | 0 | 0 | 0 | 0 | 0 | 0 | 0 | 0 | 0 | 1 | 0 | 0 | 0 | 1 |
| H781 | 16 | 13 | 29 | 23 | 9 | 10 | 11 | 14 | - | T | 0 | 0 | 0 | 0 | 0 | 0 | 0 | 0 | 0 | 0 | 0 | 0 | 0 | 1 | 0 | 0 | 0 | 1 |
| H782 | 15 | 14 | 30 | 23 | 10 | 10 | 11 | 13 | - | T | 0 | 0 | 0 | 0 | 0 | 0 | 0 | 0 | 0 | 0 | 0 | 0 | 0 | 1 | 0 | 0 | 0 | 1 |
| H783 | 12 | 13 | 29 | 20 | 10 | 11 | 13 | 14 | + | C | 0 | 0 | 0 | 0 | 0 | 0 | 0 | 0 | 0 | 0 | 0 | 0 | 0 | 1 | 0 | 0 | 0 | 1 |
| H784 | 12 | 13 | 30 | 21 | 10 | 10 | 15 | 14 | + | C | 0 | 0 | 0 | 0 | 0 | 0 | 0 | 0 | 0 | 0 | 0 | 0 | 0 | 1 | 0 | 0 | 0 | 1 |
| H785 | 12 | 14 | 30 | 24 | 9 | 10 | 12 | 12 | + | C | 0 | 0 | 0 | 0 | 0 | 0 | 0 | 0 | 0 | 0 | 0 | 0 | 0 | 1 | 0 | 0 | 0 | 1 |
| H786 | 12 | 12 | 29 | 23 | 10 | 12 | 12 | 13 | - | T | 0 | 0 | 0 | 0 | 0 | 0 | 0 | 0 | 0 | 0 | 0 | 0 | 0 | 1 | 0 | 0 | 0 | 1 |
| H787 | 12 | 12 | 28 | 25 | 10 | 10 | 12 | 14 | + | C | 0 | 0 | 0 | 0 | 0 | 0 | 0 | 0 | 0 | 0 | 0 | 0 | 0 | 1 | 0 | 0 | 0 | 1 |
| H788 | 12 | 14 | 30 | 25 | 11 | 10 | 12 | 14 | - | T | 0 | 0 | 0 | 0 | 0 | 0 | 0 | 0 | 0 | 0 | 0 | 0 | 0 | 1 | 0 | 0 | 0 | 1 |
| H789 | 13 | 14 | 31 | 22 | 11 | 12 | 13 | 17 | + | C | 0 | 0 | 0 | 0 | 0 | 0 | 0 | 0 | 0 | 0 | 0 | 0 | 0 | 0 | 2 | 0 | 0 | 2 |
| H790 | 12 | 12 | 30 | 21 | 11 | 12 | 14 | 16 | + | C | 0 | 0 | 0 | 0 | 0 | 0 | 0 | 0 | 0 | 0 | 0 | 0 | 0 | 0 | 1 | 0 | 0 | 1 |
| H791 | 12 | 12 | 31 | 21 | 11 | 12 | 14 | 16 | + | C | 0 | 0 | 0 | 0 | 0 | 0 | 0 | 0 | 0 | 0 | 0 | 0 | 0 | 0 | 1 | 0 | 0 | 1 |
| H792 | 13 | 13 | 30 | 22 | 11 | 12 | 14 | 17 | + | C | 0 | 0 | 0 | 0 | 0 | 0 | 0 | 0 | 0 | 0 | 0 | 0 | 0 | 0 | 1 | 0 | 0 | 1 |
| H793 | 12 | 12 | 29 | 21 | 11 | 12 | 14 | 16 | + | C | 0 | 0 | 0 | 0 | 0 | 0 | 0 | 0 | 0 | 0 | 0 | 0 | 0 | 0 | 1 | 0 | 0 | 1 |
| H794 | 14 | 13 | 30 | 22 | 12 | 12 | 13 | 15 | + | C | 0 | 0 | 0 | 0 | 0 | 0 | 0 | 0 | 0 | 0 | 0 | 0 | 0 | 0 | 1 | 0 | 0 | 1 |
| H795 | 13 | 15 | 32 | 22 | 11 | 12 | 14 | 17 | + | C | 0 | 0 | 0 | 0 | 0 | 0 | 0 | 0 | 0 | 0 | 0 | 0 | 0 | 0 | 1 | 0 | 0 | 1 |
| H796 | 12 | 12 | 29 | 21 | 11 | 11 | 14 | 15 | + | C | 0 | 0 | 0 | 0 | 0 | 0 | 0 | 0 | 0 | 0 | 0 | 0 | 0 | 0 | 1 | 0 | 0 | 1 |
| H797 | 12 | 15 | 31 | 21 | 12 | 12 | 13 | 15 | + | C | 0 | 0 | 0 | 0 | 0 | 0 | 0 | 0 | 0 | 0 | 0 | 0 | 0 | 0 | 1 | 0 | 0 | 1 |
| H798 | 12 | 14 | 29 | 21 | 11 | 12 | 14 | 15 | + | C | 0 | 0 | 0 | 0 | 0 | 0 | 0 | 0 | 0 | 0 | 0 | 0 | 0 | 0 | 1 | 0 | 0 | 1 |
| H799 | 12 | 13 | 32 | 21 | 11 | 12 | 13 | 16 | + | C | 0 | 0 | 0 | 0 | 0 | 0 | 0 | 0 | 0 | 0 | 0 | 0 | 0 | 0 | 1 | 0 | 0 | 1 |
| H800 | 12 | 13 | 30 | 22 | 11 | 12 | 13 | 15 | + | C | 0 | 0 | 0 | 0 | 0 | 0 | 0 | 0 | 0 | 0 | 0 | 0 | 0 | 0 | 1 | 0 | 0 | 1 |
| H801 | 12 | 15 | 33 | 21 | 11 | 12 | 15 | 15 | + | C | 0 | 0 | 0 | 0 | 0 | 0 | 0 | 0 | 0 | 0 | 0 | 0 | 0 | 0 | 1 | 0 | 0 | 1 |
| H802 | 12 | 13 | 32 | 21 | 11 | 12 | 14 | 16 | + | C | 0 | 0 | 0 | 0 | 0 | 0 | 0 | 0 | 0 | 0 | 0 | 0 | 0 | 0 | 1 | 0 | 0 | 1 |
| H803 | 13 | 14 | 30 | 22 | 11 | 12 | 12 | 17 | + | C | 0 | 0 | 0 | 0 | 0 | 0 | 0 | 0 | 0 | 0 | 0 | 0 | 0 | 0 | 1 | 0 | 0 | 1 |
| H804 | 12 | 14 | 31 | 21 | 11 | 11 | 14 | 16 | + | C | 0 | 0 | 0 | 0 | 0 | 0 | 0 | 0 | 0 | 0 | 0 | 0 | 0 | 0 | 1 | 0 | 0 | 1 |
| H805 | 12 | 14 | 31 | 22 | 11 | 12 | 13 | 15 | + | C | 0 | 0 | 0 | 0 | 0 | 0 | 0 | 0 | 0 | 0 | 0 | 0 | 0 | 0 | 1 | 0 | 0 | 1 |
| H806 | 12 | 15 | 31 | 21 | 11 | 12 | 13 | 17 | + | C | 0 | 0 | 0 | 0 | 0 | 0 | 0 | 0 | 0 | 0 | 0 | 0 | 0 | 0 | 1 | 0 | 0 | 1 |
| H807 | 13 | 13 | 31 | 21 | 11 | 12 | 14 | 15 | + | C | 0 | 0 | 0 | 0 | 0 | 0 | 0 | 0 | 0 | 0 | 0 | 0 | 0 | 0 | 1 | 0 | 0 | 1 |
| H808 | 12 | 11 | 28 | 21 | 11 | 12 | 14 | 15 | + | C | 0 | 0 | 0 | 0 | 0 | 0 | 0 | 0 | 0 | 0 | 0 | 0 | 0 | 0 | 1 | 0 | 0 | 1 |
| H809 | 12 | 13 | 32 | 21 | 11 | 12 | 14 | 15 | + | C | 0 | 0 | 0 | 0 | 0 | 0 | 0 | 0 | 0 | 0 | 0 | 0 | 0 | 0 | 1 | 0 | 0 | 1 |
| H810 | 12 | 14 | 33 | 21 | 11 | 12 | 15 | 17 | + | C | 0 | 0 | 0 | 0 | 0 | 0 | 0 | 0 | 0 | 0 | 0 | 0 | 0 | 0 | 0 | 1 | 1 | 2 |
| H811 | 12 | 15 | 32 | 21 | 11 | 12 | 14 | 17 | + | C | 0 | 0 | 0 | 0 | 0 | 0 | 0 | 0 | 0 | 0 | 0 | 0 | 0 | 0 | 0 | 1 | 0 | 1 |
| H812 | 12 | 13 | 30 | 21 | 11 | 12 | 15 | 15 | + | C | 0 | 0 | 0 | 0 | 0 | 0 | 0 | 0 | 0 | 0 | 0 | 0 | 0 | 0 | 0 | 1 | 0 | 1 |
| H813 | 12 | 14 | 32 | 21 | 11 | 11 | 12 | 15 | + | C | 0 | 0 | 0 | 0 | 0 | 0 | 0 | 0 | 0 | 0 | 0 | 0 | 0 | 0 | 0 | 1 | 0 | 1 |
| H814 | 12 | 14 | 33 | 21 | 11 | 12 | 13 | 15 | + | C | 0 | 0 | 0 | 0 | 0 | 0 | 0 | 0 | 0 | 0 | 0 | 0 | 0 | 0 | 0 | 1 | 0 | 1 |
| H815 | 12 | 14 | 30 | 21 | 11 | 13 | 13 | 14 | + | C | 0 | 0 | 0 | 0 | 0 | 0 | 0 | 0 | 0 | 0 | 0 | 0 | 0 | 0 | 0 | 1 | 0 | 1 |
| H816 | 12 | 13 | 30 | 21 | 11 | 12 | 15 | 16 | + | C | 0 | 0 | 0 | 0 | 0 | 0 | 0 | 0 | 0 | 0 | 0 | 0 | 0 | 0 | 0 | 1 | 0 | 1 |
| H817 | 12 | 13 | 33 | 21 | 11 | 12 | 13 | 15 | + | C | 0 | 0 | 0 | 0 | 0 | 0 | 0 | 0 | 0 | 0 | 0 | 0 | 0 | 0 | 0 | 1 | 0 | 1 |
| H818 | 12 | 13 | 31 | 21 | 12 | 11 | 13 | 17 | + | C | 0 | 0 | 0 | 0 | 0 | 0 | 0 | 0 | 0 | 0 | 0 | 0 | 0 | 0 | 0 | 1 | 0 | 1 |
| H819 | 12 | 13 | 32 | 21 | 11 | 12 | 12 | 16 | + | C | 0 | 0 | 0 | 0 | 0 | 0 | 0 | 0 | 0 | 0 | 0 | 0 | 0 | 0 | 0 | 1 | 0 | 1 |
| H820 | 12 | 13 | 30 | 21 | 11 | 12 | 13 | 14 | + | C | 0 | 0 | 0 | 0 | 0 | 0 | 0 | 0 | 0 | 0 | 0 | 0 | 0 | 0 | 0 | 0 | 1 | 1 |
| H821 | 12 | 13 | 31 | 21 | 12 | 12 | 13 | 16 | + | C | 0 | 0 | 0 | 0 | 0 | 0 | 0 | 0 | 0 | 0 | 0 | 0 | 0 | 0 | 0 | 0 | 1 | 1 |
| H822 | 12 | 13 | 31 | 21 | 11 | 12 | 12 | 17 | + | C | 0 | 0 | 0 | 0 | 0 | 0 | 0 | 0 | 0 | 0 | 0 | 0 | 0 | 0 | 0 | 0 | 1 | 1 |

| | | | | | | | | | | | | | | | | | | | | | | | | | | | | |
|---|---|---|---|---|---|---|---|---|---|---|---|---|---|---|---|---|---|---|---|---|---|---|---|---|---|---|---|---|
| H823 | 12 | 12 | 29 | 25 | 12 | 12 | 13 | 14 | + | C | 0 | 0 | 0 | 0 | 0 | 0 | 0 | 0 | 0 | 0 | 0 | 0 | 0 | 0 | 0 | 0 | 1 | 1 |
| H824 | 13 | 13 | 30 | 21 | 11 | 12 | 14 | 17 | + | C | 0 | 0 | 0 | 0 | 0 | 0 | 0 | 0 | 0 | 0 | 0 | 0 | 0 | 0 | 0 | 0 | 1 | 1 |
| H825 | 12 | 12 | 30 | 21 | 11 | 12 | 14 | 17 | + | C | 0 | 0 | 0 | 0 | 0 | 0 | 0 | 0 | 0 | 0 | 0 | 0 | 0 | 0 | 0 | 0 | 3 | 3 |
| H826 | 12 | 12 | 29 | 21 | 11 | 12 | 12 | 16 | + | C | 0 | 0 | 0 | 0 | 0 | 0 | 0 | 0 | 0 | 0 | 0 | 0 | 0 | 0 | 0 | 0 | 1 | 1 |
| H827 | 12 | 12 | 30 | 21 | 11 | 12 | 12 | 17 | + | C | 0 | 0 | 0 | 0 | 0 | 0 | 0 | 0 | 0 | 0 | 0 | 0 | 0 | 0 | 0 | 0 | 1 | 1 |
| H828 | 12 | 13 | 31 | 21 | 11 | 11 | 12 | 17 | + | C | 0 | 0 | 0 | 0 | 0 | 0 | 0 | 0 | 0 | 0 | 0 | 0 | 0 | 0 | 0 | 0 | 1 | 1 |
| H829 | 13 | 11 | 28 | 22 | 12 | 12 | 13 | 13 | + | C | 0 | 0 | 0 | 0 | 0 | 0 | 0 | 0 | 0 | 0 | 0 | 0 | 0 | 0 | 0 | 0 | 1 | 1 |
| H830 | 13 | 14 | 31 | 21 | 11 | 12 | 15 | 17 | + | C | 0 | 0 | 0 | 0 | 0 | 0 | 0 | 0 | 0 | 0 | 0 | 0 | 0 | 0 | 0 | 0 | 1 | 1 |
| H831 | 12 | 12 | 30 | 22 | 11 | 13 | 11 | 17 | + | C | 0 | 0 | 0 | 0 | 0 | 0 | 0 | 0 | 0 | 0 | 0 | 0 | 0 | 0 | 0 | 0 | 1 | 1 |
| H832 | 12 | 13 | 29 | 21 | 10 | 11 | 14 | 16 | + | C | 0 | 0 | 0 | 0 | 0 | 0 | 0 | 0 | 0 | 0 | 0 | 0 | 0 | 0 | 0 | 0 | 1 | 1 |
| H833 | 12 | 14 | 31 | 21 | 11 | 11 | 15 | 17 | + | C | 0 | 0 | 0 | 0 | 0 | 0 | 0 | 0 | 0 | 0 | 0 | 0 | 0 | 0 | 0 | 0 | 1 | 1 |
| H834 | 12 | 13 | 31 | 21 | 11 | 12 | 15 | 15 | + | C | 0 | 0 | 0 | 0 | 0 | 0 | 0 | 0 | 0 | 0 | 0 | 0 | 0 | 0 | 0 | 0 | 1 | 1 |
| H835 | 12 | 15 | 32 | 21 | 11 | 12 | 13 | 15 | + | C | 0 | 0 | 0 | 0 | 0 | 0 | 0 | 0 | 0 | 0 | 0 | 0 | 0 | 0 | 0 | 0 | 1 | 1 |
